# COVID-19 risk mitigation in reopening mass cultural events: population-based observational study for the UK Events Research Programme in Liverpool City Region

**DOI:** 10.1177/01410768231182389

**Published:** 2023-06-23

**Authors:** Girvan Burnside, Christopher P Cheyne, Gary Leeming, Michael Humann, Alistair Darby, Mark A Green, Alexander Crozier, Simon Maskell, Kay O’Halloran, Elena Musi, Elinor Carmi, Naila Khan, Debra Fisher, Rhiannon Corcoran, Jake Dunning, W John Edmunds, Kukatharmini Tharmaratnam, David M Hughes, Liora Malki-Epshtein, Malcolm Cook, Ben M Roberts, Eileen Gallagher, Kate Howell, Meera Chand, Robin Kemp, Matthew Boulter, Tom Fowler, Malcolm G Semple, Emer Coffey, Matt Ashton, The COVID-19 Genomics UK (COG-UK) Consortium, Marta García-Fiñana, Iain E Buchan

**Affiliations:** 1 Deparment of Health Data Science, Institute of Population Health, University of Liverpool, Liverpool L69 3GL, UK; 2 Department of Psychology, University of Liverpool, Liverpool L69 7ZA, UK; 3 Department of Infection Biology and Microbiomes, Institute of Infection, Veterinary and Ecological Sciences, University of Liverpool, Liverpool L69 3BX, UK; 4 Department of Geography and Planning, University of Liverpool L69 3BX, Liverpool, UK; 5 Division of Biosciences, University College London, London WC1E 6BT, UK; 6 Department of Electrical Engineering and Electronics, University of Liverpool, Liverpool L69 3BX, UK; 7 Department of Communication and Media, University of Liverpool, Liverpool L69 7ZG, UK; 8 Department of Sociology and Criminology, City University, London EC1V 0HB, UK; 9 Department of Primary Care & Mental Health, Institute of Population Health, University of Liverpool, Liverpool L69 3BX, UK; 10 Pandemic Sciences Institute, University of Oxford, Oxford OX3 7DQ, UK; 11 Centre for Mathematical Modelling of Infectious Diseases and Department of Infectious Disease Epidemiology, London School of Hygiene and Tropical Medicine, London WC1E 7HT, UK; 12 Department of Civil, Environmental and Geomatic Engineering, University College London, London WC1E 6BT, UK; 13 Building Energy Research Group, School of Architecture, Building and Civil Engineering, Loughborough University, Loughborough LE11 3TU, UK; 14 Clinical and Public Health Group, UK Health Security Agency, London SW1P 3JR, UK; 15 William Harvey Research Institute, Queen Mary University of London, London EC1M 6BQ, UK; 16 Liverpool City Council, Liverpool L3 1AH, UK; 17 Department of Clinical Infection, Microbiology & Immunology, Institute of Infection, Veterinary and Ecological Sciences, University of Liverpool, Liverpool L69 3BX, UK; 18 Department of Public Health, Policy and Systems, Institute of Population Health, University of Liverpool, Liverpool L69 3GB, UK; *Shared senior authorship.

**Keywords:** COVID-19, mass gatherings, cultural events, SARS-CoV-2 transmission, respiratory virus risk mitigation

## Abstract

**Objectives:**

To understand severe acute respiratory syndrome coronavirus 2 (SARS-CoV-2) transmission risks, perceived risks and the feasibility of risk mitigations from experimental mass cultural events before coronavirus disease 2019 (COVID-19) restrictions were lifted.

**Design:**

Prospective, population-wide observational study.

**Setting:**

Four events (two nightclubs, an outdoor music festival and a business conference) open to Liverpool City Region UK residents, requiring a negative lateral flow test (LFT) within the 36 h before the event, but not requiring social distancing or face-coverings.

**Participants:**

A total of 12,256 individuals attending one or more events between 28 April and 2 May 2021.

**Main outcome measures:**

SARS-CoV-2 infections detected using audience self-swabbed (5–7 days post-event) polymerase chain reaction (PCR) tests, with viral genomic analysis of cases, plus linked National Health Service COVID-19 testing data. Audience experiences were gathered via questionnaires, focus groups and social media. Indoor CO_2_ concentrations were monitored.

**Results:**

A total of 12 PCR-positive cases (likely 4 index, 8 primary or secondary), 10 from the nightclubs. Two further cases had positive LFTs but no PCR. A total of 11,896 (97.1%) participants with scanned tickets were matched to a negative pre-event LFT: 4972 (40.6%) returned a PCR within a week. CO_2_ concentrations showed areas for improving ventilation at the nightclubs. Population infection rates were low, yet with a concurrent outbreak of >50 linked cases around a local swimming pool without equivalent risk mitigations. Audience anxiety was low and enjoyment high.

**Conclusions:**

We observed minor SARS-CoV-2 transmission and low perceived risks around events when prevalence was low and risk mitigations prominent. Partnership between audiences, event organisers and public health services, supported by information systems with real-time linked data, can improve health security for mass cultural events.

## Introduction

Governments worldwide restricted mass gatherings in response to the coronavirus disease 2019 (COVID-19) pandemic to reduce severe acute respiratory syndrome coronavirus 2 (SARS-CoV-2) transmission.^1^ Events such as music festivals, business conferences and nightclubs are characterised by mixing in close proximity, often in poorly ventilated spaces over long periods. These characteristics have been linked to ‘super-spreading’.^2,3^ Limiting the size of gatherings or cancelling events reduced infections.^4,5^ Such measures, however, come at a cost to public wellbeing and the economy.

More than a year of cancelled events during 2020–2021 damaged industries that require mass gatherings, with many people losing their livelihoods.^6^ In addition, the support of social fabric and mental wellbeing from cultural events was lost. This disproportionally affected younger people, who were last to be vaccinated and hit hardest hit by job losses and restricted social mixing.^7^ As such, some countries experimented with reopening mass events. One randomised controlled trial of attendance at an indoor nightclub in Barcelona with 473 attendees showed no transmission among participants,^8^ although levels of risk mitigation included compulsory N95 mask wearing and maximised ventilation, which do not reflect how events can run sustainably. At the Dutch FieldLab experiment (music festival for ∼1500 participants in March 2021), the subgroup assigned to mask wearing tended to take their masks off in the dance tent.^9^ Other COVID-19 testing protocols researched at events included regular reverse-transcription polymerase chain reaction (PCR) and rapid antigen testing on the PGA European golf tour,^10,11^ which were unaffordable and impractical for many events.

From Summer 2021, the events sector reopened around the world, with temporary returns to lockdowns in some countries and regions. The World Health Organization issued ‘Strategy considerations for SARS-CoV-2 and other respiratory viruses in the WHO European Region during autumn and winter 2022/23: protecting the vulnerable with agility, efficiency, and trust’.^12^ This marked a shift from reduction of transmission en masse to protecting the vulnerable, following evidence of net harms from blanket control measures.^13,14^

We present findings from the UK’s Events Research Programme (ERP) response to the COVID-19 pandemic, relevant to future respiratory virus pandemic preparedness and mass cultural events. The ERP was developed to generate evidence on the reopening of events, assessing the risk of SARS-CoV-2 transmission, and to pilot risk-mitigation measures in line with the UK Government’s Roadmap for ‘reopening’ society.^15^ The first phase of the ERP included nine pilot events with various measures to prevent and contain SARS-CoV-2 transmission.^16^ Four of these events took place in Liverpool between 28 April and 2 May 2021, including a nightclub (on two consecutive nights), an outdoor music festival with a tented dance area and a business conference. Audiences were invited from residents of Liverpool City Region only, enabling a population-based study of transmission. Attendees required a negative rapid antigen lateral flow test (LFT) at an asymptomatic testing site within 36 h prior to the event and were encouraged not to attend if they had symptoms. Social distancing and face coverings were not required, thus reflecting how the events sector could reopen sustainably. This study aimed to evaluate SARS-CoV-2 transmission, public and audience experiences and public health operational requirements for running COVID-19 risk-mitigated events.

## Methods

### Study design

Adult residents (18+ years) of Liverpool City Region were invited to express interest in attending one or more test events – via usual advertising and general media communications. Individuals were invited to consent to participate in the ERP and complete a pre-event questionnaire online. Those who consented and completed the questionnaire could purchase a ticket and were directed to take a rapid SARS-CoV-2 antigen LFT^17^ at a supervised asymptomatic testing centre within the 36 h before the event, and not to attend if they had any symptoms listed on Government/National Health Service (NHS) websites. Positive test results were reviewed by the local public health team, who then contacted individuals to inform them to self-isolate and not attend. Close contacts of test-positive individuals were traced and asked to test and not to attend any test events. Tickets were cancelled and refunded for those testing positive. Participants were given two swabs at the pre-event, asymptomatic testing centre, to return for PCR testing: one on the day of the event, and one 5 days post-event. After the event, participants were asked to complete another questionnaire. Consent was obtained to link participant details to routinely collected NHS data to identify any PCRs or LFTs taken by participants pre- or post-event. All attendees of the events were included in the study.

Data were collected in pre-event questionnaires on attitudes to the test events. Individuals were asked their age, address, sex and ethnicity and if they were concerned about catching and/or transmitting COVID-19 at the event. Post-event questionnaires captured attitudes towards COVID-19 certification, including vaccine passports, as a requirement for attending future events.

### Data linkage

Participants gave consent for linkage of their questionnaire responses and ticket data to NHS and administrative records. The residential address was linked to the Index of Multiple Deprivation (2019) at Lower-Layer Super Output Area.^18^ COVID-19 testing and vaccination data were linked via the NHS Combined Intelligence for Population Health Action (CIPHA) system.^19^ CIPHA provided near real-time (updated every 30 min) NHS Test & Trace results and vaccination status, and has supported COVID-19 responses and national studies previously.^17,20^ We linked participants’ consent records, survey data and ticket information to NHS data within CIPHA using fuzzy matching based on name, postcode and date of birth to look up NHS number for test result matching. We used this system to validate tickets (as holder test-negative) and gather study data including age, sex, address, COVID-19 LFT and PCR test results (including previous positive results in 2021), genomic analysis of positive cases and vaccination status.

The University of Liverpool Research Ethics Committee approved the study (Approval 8486, 25 Nov 2020: amended 31 Mar 2021) before commencement.

### Classification of cases

Attendees were classified as potential index cases if they had a positive PCR swab in the 24 h before, or up to 72 h after, the start of the event, using home-test kits handed out at pre-event testing centres. Those with positive PCR swab results between 4 and 7 days post-event were classified as possible primary (infected by index case at the event) or secondary cases (infected by primary case after the event). A probabilistic classification tool was also used, adjudicated by experts in relevant viral dynamics (supplementary Appendix 2, P1).

### Statistical analyses

Analyses were carried out on pseudonymised data; those undertaking analyses did not have access to person-identifiable information.

All participants who attended any event were included in the study cohort. Descriptive statistics on attendees of each event, and overall, were generated. Multiple logistic regression was used to identify factors associated with the likelihood of returning a PCR test within 7 days after event. Models were fitted per event and overall. Statistical analyses were carried out in R (version 3.6.1 or later). Details in supplementary Appendix 2, P1.

### Additional data collection and analysis

The ERP at Liverpool incorporated a wide range of quantitative and qualitative research methods, data collection and analyses. Genomic analysis was performed using civet 3.0 (Cluster Investigation and Virus Epidemiology Tool https://github.com/artic-network/civet) with CLIMB background genomic data for the relevant time periods generated by the COG-UK consortium. Indoor venue air CO_2_ concentrations were measured as a proxy for exposure to exhaled breath at two venues (nightclub and conference centre) (supplementary Appendix 2, P2). Eight focus groups were run with attendees (supplementary Appendix 2, P2) and media reports and social media posts were examined (supplementary Appendix 2, P3–5). Public health intelligence systems were used to examine COVID-19 outbreaks within 2 weeks before/after the ERP events.

### Role of the funding source

This evaluation was commissioned via the UK Government’s Department for Digital, Culture, Media and Sport (DCMS) as part of ERP and used the UK Government’s Department of Health & Social Care Test & Trace infrastructure. The University of Liverpool independently analysed the study data and reported the findings to DCMS.

## Results

**Figure 1.**
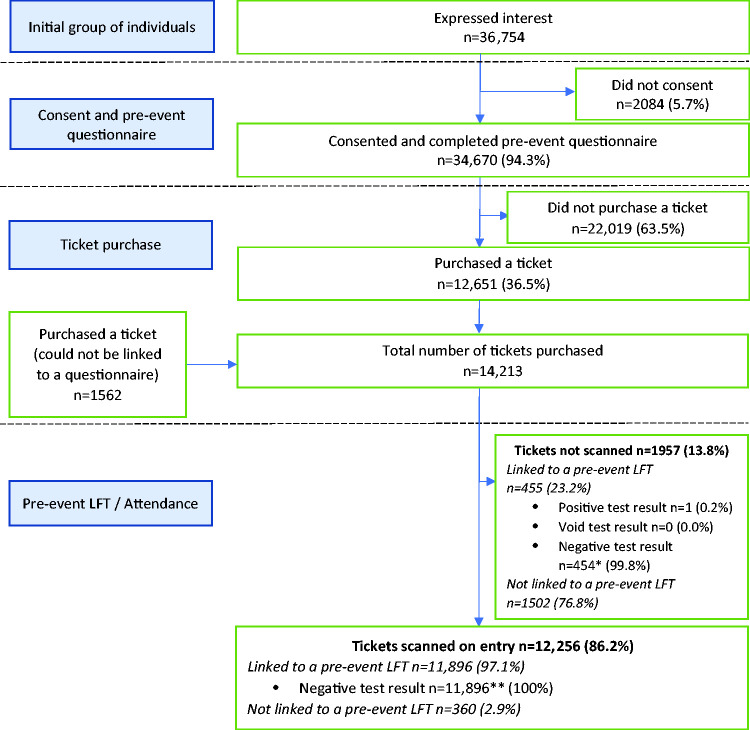
Pre-event participant flow diagram for all events combined. *Three of these 454 were preceded by a positive LFT (one of which also had a void LFT) and one was preceded by a void LFT. **One of these 11,896 was preceded by a positive LFT and 20 were preceded by a void LFT.LFT: lateral flow test.

**Figure 2.**
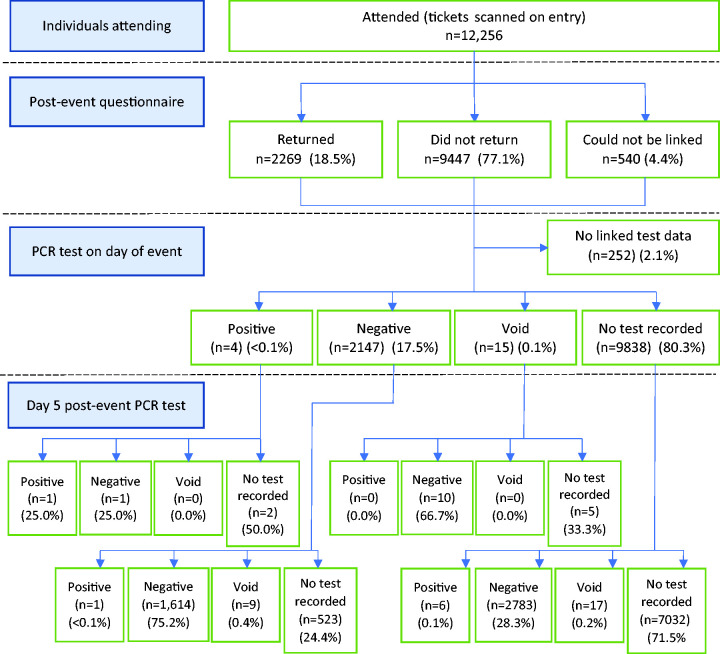
Post-event participant flow diagram for all events combined. Note: One positive case is not included, as they were not scanned on entry to the event but reported that they did attend.PCR: polymerase chain reaction.

A total of 36,754 individuals expressed interest in attending the events. A total of 34,670 (94.3%) consented to take part and completed a pre-event questionnaire, of which 12,651 (36.5%) purchased a ticket. A total of 1562 tickets could not be linked to a pre-event questionnaire, so were acquired outside the main booking system. A total of 12,256 individuals (86%; 12,256/14,213) were recorded as entering one or more events, with a total of 13,262 attendances. Multiple events were attended by 8% of people. Overall flow diagrams pre- and post-event are shown in Figures 1 and 2, with diagrams for individual events in supplementary Appendix 1.

The demographic characteristics of attendees are shown in Table 1. Participants were largely young (with older, more likely vaccinated attendees at the business conference) and predominantly from white ethnic backgrounds and deprived areas. Attendees of the nightclub and music festival resided in areas with younger and student populations (supplementary Appendix 3, P1). Vaccination rates among attendees were low, as most younger people had not yet been offered a vaccine.

**Table 1.** Descriptive statistics of the characteristics of people who attended the Liverpool events.

Characteristic	All events*n* = 12 256	Nightclub ×2*n* = 6802	Music festival*n* = 6101	Conference*n* = 149
Age (Median, IQR)	21 (19,25)	20 (19, 23)	22 (20, 27)	44 (33, 51)
*n* missing	79	39	40	0
Sex				
Female	5982 (50.0%)	3232 (48.7%)	3159 (52.8%)	77 (52.7%)
Male	5991 (50.0%)	3410 (51.3%)	2822 (47.2%)	69 (47.3%)
Missing data	283	160	120	3
Ethnicity				
White	10,701 (89.3%)	5857 (88.1%)	5455 (91.1%)	107 (73.3%)
Another ethnic group	32 (0.3%)	21 (0.3%)	12 (0.2%)	1 (0.7%)
Asian or Asian British	228 (1.9%)	156 (2.3%)	93 (1.6%)	3 (2.1%)
Black or Black British	73 (0.6%)	56 (0.8%)	15 (0.3%)	2 (1.4%)
Mixed ethnicity	296 (2.5%)	191 (2.9%)	126 (2.1%)	3 (2.1%)
Prefer not to say	654 (5.5%)	368 (5.5%)	284 (4.7%)	30 (20.5%)
Missing data	272	153	116	3
IMD quintile^a^				
1 (Most deprived)	4234 (36.4%)	2280 (35.3%)	2088 (35.8%)	44 (31.9%)
2	2828 (24.3%)	1682 (26.0%)	1345 (23.0%)	24 (17.4%)
3	2702 (23.2%)	1525 (23.6%)	1465 (25.1%)	26 (18.9%)
4	1202 (10.3%)	575 (8.9%)	645 (11.0%)	30 (21.7%)
5 (Least deprived)	681 (5.8%)	400 (6.2%)	297 (5.1%)	14 (10.1%)
Missing data	609	340	261	11
Vaccinated				
No	9002 (73.7%)	5346 (78.9%)	4174 (68.5%)	50 (33.8%)
Yes	3215 (26.3%)	1427 (21.1%)	1918 (31.5%)	98 (66.2%)
Missing data	39	29	9	1
Had SARS-CoV-2 in 2021				
No	11,193 (95.0%)	6267 (95.5%)	5579 (94.9%)	133 (96.4%)
Yes	590 (5.0%)	296 (4.5%)	299 (5.1%)	5 (3.6%)
Missing data	473	239	223	11
Returned PCR within 7 days				
No	7032 (58.6%)	4506 (67.7%)	2925 (48.8%)	64 (43.8%)
Yes	4972 (41.4%)	2149 (32.3%)	3074 (51.2%)	82 (56.2%)
Missing data	252	147	102	3
Concern at infecting others				
Some concern	5786 (51.0%)	2941 (48.3%)	3142 (53.7%)	85 (57.4%)
Not at all concerned	5568 (49.0%)	3145 (51.7%)	2706 (46.3%)	63 (42.6%)
Missing data	902	716	253	1
Vaccination passport				
Opposed	329 (16.1%)	160 (20.2%)	172 (13.5%)	4 (11.8%)
Indifferent or in favour	1712 (83.9%)	631 (79.8%)	1106 (86.5%)	30 (88.2%)
Missing data	10,215	6011	4823	115

a Index of Multiple Deprivation (IMD) quintiles are based on national reference.

Full descriptive statistics of questionnaire responses are shown in supplementary Appendix 6.

Of the 12,256 attendees with tickets scanned, 11,896 (97%) could be matched to a pre-event LFT, all of which were negative. For 360 attendees (3%), no pre-event LFT data could be linked (supplementary Appendix 1).

Table 2 describes attendees who had a positive PCR test in the pre- or post-event windows. Of 12,256 attendees, 2151 (18%) were matched to a non-void PCR test result in the first window (days 0 to 3). Four of these PCRs were positive and regarded as potential index cases (0.2%). A total of 4416 (36%) attendances could be matched to a non-void PCR test result in the second window (days 4 to 7). Eight of these tests were positive (0.2%). Of these, one had already tested positive in the first window. The remaining seven were regarded as potential primary or secondary cases. One additional positive test in the second window was found from a ticketholder who did not have their ticket scanned, but reported attendance, giving a total of eight likely primary or secondary cases. In both time windows, 1617 (13%) attendances had non-void PCR test results.

**Table 2.** Potential index cases (days 0–3) and likely primary or secondary cases (days 4–7) identified from positive PCR tests.

Event	Case number	Estimated day of swab (day of event = day 0)	Ct value	Variant (C = confirmed, P = probable)^a^	Notes	Likely classification using Ct values and contact-tracing info^b^
Potential index cases from PCR tests days 0–3						
Music festival	1	Day 3	32	Alpha (P)	Also tested positive 18 days before event	Low-index
Nightclub (Friday)	2	Day 0	33	Alpha (P)	Also tested negative on day 1	Unrelated
Nightclub (Friday)	3	Day 0	21	Alpha (P)		High-index
Nightclub (Saturday)	4	Day 2	22	Alpha (C)	Further positive tests on days 7, 9 and 16	Low-index
Likely primary or secondary cases from PCR tests days 4–7						
Music festival	5	Day 6	33	Alpha (P)		High-index or secondary
Nightclub (Friday)	6	Day 5	26	Delta (C)		Primary
Nightclub (Friday)	7	Day 5	32	Alpha (P)		High-index or secondary
Nightclub (Friday)	8	Day 5	20	Delta (C)		Primary
Nightclub (Saturday)	9	Day 5	24	Alpha (C)	Further positive test on day 7	Secondary or unrelated
Nightclub (Saturday)	10	Day 7	18	Alpha (C)	Further positive test on day 9	Primary
Nightclub (Saturday)	11	Day 5	13	Alpha (C)		Primary
Nightclub (Saturday)	12	Day 7	15	Alpha (C)		Primary
Additional potential index cases from positive LFTs						
Music festival	13	Day −1	N/A	Unknown	Both positive and negative LFTs the day before the event	High-index
Music festival	14	Day 1	N/A	Unknown	Positive LFT, no PCRs matched	High-index
Additional potential index cases from PCR test outside testing window						
Nightclub (Saturday)	15	Day −8	13	Alpha (C)	Negative LFT the day before the event	Low-index

Ct: cycle threshold; LFT: lateral flow test; PCR: polymerase chain reaction.

a C = confirmed variant from genomic sequencing, P = probable variant based on whether S-gene target was detected.

b Index cases would arrive at the event already infected (sub-categorised into high and low viral load), primary cases would be infected at the event by an index case, secondary cases would be infected by a primary case after the event, unrelated cases would be infected by someone not at the event.

Of the 12 cases described above, one potential index case (number 4 in Table 2), and two likely secondary cases (numbers 8 and 10) were identified from symptomatic PCR tests carried out at NHS testing centres. The remaining nine cases came from return of research PCR swabs issued pre-event. Although the Delta variant was starting to spread in the population from which the audience was drawn, where virus genome data were available, three of five cases from the Friday nightclub, all six cases from the Saturday nightclub and two cases from the music festival showed S-gene target-failure, indicating infection with the Alpha variant that was most prevalent in the community.

Further examination of LFT and PCR results taken outside the pre- and post-event windows identified three additional potential index cases (cases 13–15).

One inclusion criterion was that attendees should not have received a positive PCR result in the 30 days prior to the event. We did not cross-check this with NHS records prior to admission. A total of 10 ticketholders with positive PCR tests in the prior 30 days attended events (supplementary Appendix 3, P4, all showed a negative pre-event LFT). One tested positive 8 days before attending (case 15) and the remaining nine tested positive more than 2 weeks before the event.

Two ticketholders with positive LFTs prior to the event were subsequently scanned into the event (supplementary Appendix 3, P5). One had a positive LFT 3 days pre-event but received negative results from both a PCR and a second LFT prior to attending. The other received a positive result, then went to a different test centre later the same day for a second test, which was negative (case 13).

Eight attendees had a positive LFT result within a week after attending events (supplementary Appendix 3, P5), of whom two (cases 8 and 10) had a concordant positive PCR (either from ERP-issued tests or NHS symptomatic testing sites), five had discordant PCR within 7 days and one had no PCR test recorded (case 14).

A combination of contact-tracing information, PCR (including cycle threshold: Ct) and LFT results and symptoms were used to make more detailed estimates of whether participants were likely to be infectious at the event, have become infected at the events or have become infected later due to further contact with attendees. The results are shown in the final column of Table 2, with more details in supplementary Appendix 3, P2–3.

Viral genomic sequencing was available for eight attendees with positive PCR tests. Two Friday nightclub attendees (who attended together) had confirmed delta variant (cases 6 and 8) with similar genetic lineage to cases detected from UK surveillance sequencing in the Merseyside area in the same week. One of the two reported symptoms the next day, with family members having been symptomatic pre-event. All six Saturday nightclub attendees with positive PCRs had alpha variant confirmed. Phylogenetic tree analysis grouped five of these together with similar lineage, including a friendship group of four confirmed by contact tracing (cases 4, 9, 10 and 12), and a fifth from outside Merseyside (case 11). The closest UK surveillance cases on the tree to these were all in Merseyside, suggesting linked community transmission. Further analysis of these five cases showed two distinct genomic groupings, cases 4 and 9, and cases 10, 11 and 12. The sixth case from Saturday was case 15, who had tested positive 8 days pre-event. This attendee was in a distinct catchment from the others, suggesting they were unlikely to be a linked index case. Tree diagrams are shown in supplementary Appendix 3, P10–12.

Further positive tests were matched for 67 attendees after post-event follow-up (supplementary Appendix 3, P6–7), with final data extract taken on 10 June, corresponding to day 43 for the first event (conference) and day 39 for the last (outdoor music festival). All participants testing positive after the post-event window were S-gene target positive, indicating infection with the Delta variant, and not the Alpha variant that was dominant during the events.

### Factors associated with PCR test return

Exploratory analysis of factors associated with PCR return indicated that male individuals, younger people, attendees of Black or Black British ethnicity, those who were not fully vaccinated, those who had tested positive for SARS-Cov2 in 2021 and those who expressed no concern about infecting others at the event had lower comparative odds of returning a PCR swab. Individuals attending the music festival, which offered an incentive to return PCR tests, had higher odds. Details in supplementary Appendix 3, P8.

### Indoor venue air CO_2_ analysis

Analysis of indoor venue CO_2_ concentrations showed acceptable or good ventilation at the business event, but high variation at the nightclub events, indicating localised areas of poor ventilation and crowding associated with high occupancy close to the stage. Details in supplementary Appendix 4, P1–2.

### Focus groups

Some apprehension was expressed prior to the events over fear of transmitting SARS-CoV-2 to other people. Some participants expressed initial uncertainty and anxiety about ticket issuing linked to a negative test result. The transition away from social distancing was received very well:

*And I was quite anxious before going to the event that I would find it very uncomfortable to be in an environment with so many people. It’s gone from nothing to all, if you like, in the space of half an hour. But amazingly, I felt completely safe.*

Others expressed that abandoning social distancing measures and not wearing masks felt strange at first, although once inside the venue, behaviour reverted rapidly to non-socially distanced interactions. Despite initial feelings of anxiety for some, all participants quickly reverted to natural pre-COVID socialising.

*I did think originally that I might keep my mask on but then when I got in there I thought, ‘No, take the mask off, I don’t feel that I need this.’ I should add, I have had my first jab because I'm a lot older than most of the people probably there, so I had had one jab which also made me feel a bit more comfortable, but I felt safe.*

At the conference, an area had been set aside for those wanting to socially distance, but this was not used.

Social distancing was reported as impossible at the egress from the music festival due to large numbers preferring to keep in groups, despite guidance not to.

Most participants felt safe at events, and this was clearly associated with the requirement to have a negative test prior to attendance. In addition, vaccinated participants reported feeling safe due to immunisation. Some anxiety about unvaccinated people attending future events was expressed.

### Digital and social media analysis

A total of 367 media articles from 15 April to 15 June 2021 were examined. Computational sentiment and qualitative analyses showed that the Liverpool ERP was endorsed and promoted through official channels. Sentiment scores were positive and high, with content focused on entertainment aspects. However, an analysis of 4282 comments posted in response to the media articles showed public reactions were polarised, which was also reflected in the sentiment scores ranging from extremely positive to negative, averaging as a neutral score. Analysis of 2144 public Tweets (including 831 retweets) showed a diverse range of views over the events or associated publicity, and the average sentiment score was positive.

Discussions about falsifying LFTs were found in a small number of Tweets (38), with a negative sentiment indicating disapproval of this behaviour. Public comments (1320) condemned six TikTok videos over practising with test kits to fake negative results, especially regarding wastage of kits. By contrast, 2500 comments on 50 TikTok videos showing how to fake positive results ranged from amusement to condemnation, again focusing concern on waste of kits.

Further detail is in supplementary Appendix 4.

### Concurrent outbreaks and clusters of cases

The 7-day rolling rate of new cases in Liverpool on the first day of the events was 13.6 per 100,000 population.^21^ Data on outbreaks and clusters in Liverpool City Region concurrent with events identified several foci of linked cases, including one super-spreading event associated with a swimming pool with more than 50 linked cases, which did not have the ERP risk-mitigations.

## Discussion

We present the first population-based evidence of actual and perceived risks of SARS-CoV-2 transmission around the early reopening of mass events before COVID-19 restrictions were lifted. To our knowledge, this is the only evidence of its kind internationally.

The people of Liverpool City Region were invited to attend four experimental events in April and May 2021 as part of the UK’s ERP. Of the 12,256 individuals attending one or more events over 5 days, there were 15 linked cases detected through research, public health and clinical testing using population-wide linked data. Half of the cases were likely primary or secondary, reflecting transmission no higher than the background rate, in contrast to a concurrent outbreak of more than 50 linked cases associated with a local swimming pool.

Audiences were free to mix without face-coverings, at a time when mass gatherings were banned, and face-coverings were required at smaller gatherings. Risk mitigations included: requirement to test negative for SARS-CoV-2 antigen in the 36 h pre-event; prompt contact tracing including real-time linked ticketing and testing data; and repeated communications asking audiences to minimise contacts in the week before/after the event, to take usual precautions in travelling to/from the event and not to attend if experiencing any official COVID-19 symptoms.

Participant concerns over SARS-CoV-2 transmission risks declined during and after events, and enjoyment levels were high. There was relatively little (16%) opposition to the potential introduction of ‘vaccine passports’ for future mass gatherings, although the response rate was low (17%), and non-responders may have been more opposed. This contrasted with some social media posts opposing any certification, especially vaccine passports. Before the events, some organisers and researchers received threats citing opposition to COVID-19 certification, with one prospective event pulling out. Public sentiments on digital and social media were polarised between strong support for reopening of events and concern over it being ‘too early’. Tweeted sentiments were largely positive, as was media coverage.

Incentivisation and good communication may have led to participation in optional post-event testing being higher than at most other ERP events.^16^ An event (music festival) offering incentives, outside national ERP protocol, showed higher test returns, although other event-specific factors may have influenced this.

The key strengths of this study are its population-wide design and the realistic way the events were run. Liverpool was the first city in the world to introduce voluntary open-access asymptomatic testing, and has used real-time linked data systems to study patterns of SARS-CoV-2 transmission and coordinate public health responses since November 2020.^20,22^ Liverpool’s NHS and public health intelligence system (www.cipha.nhs.uk) was extended to incorporate ticketing and questionnaire data with consent. A mixed-methods approach enabled consideration of a broad range of demographic, behavioural and attitudinal factors affecting participants’ experiences.

Over one-third of Liverpool’s economy is linked to events, visitors and hospitality,^23^ and strong existing relationships between event organisers, local authority events and public health teams enabled venues and operations to be stood up quickly and realistically. Mask wearing has been identified as unsustainable by the UK Department of Health & Social Care Project Encore, which became the ERP.

There were some limitations. Postal return of PCR swabs was low, although the linked data systems captured all NHS and public health service COVID-19 test (symptomatic and asymptomatic) results in the study population, with over 98% of participants being matched to NHS number, ensuring identification of registered test results. Some cases could have been missed, particularly if infected participants were asymptomatic, did not return a research PCR or were symptomatic and did not seek an NHS test. This means that our data are likely to underestimate transmission risk at the events. We did not aim accurately to quantify SARS-CoV-2 transmission, but to use all available data to detect any major outbreaks. These data were sufficient to detect a concurrent unrelated outbreak in the study population. This suggests that any significant outbreak linked to the events would have been detected in our data. Data linkage between tickets and test results was incomplete, but very high (98%) relative to other ERP events,^16^ and available before the start of the events enabling preventive outreach to ticketholders and their contacts. Participant demographics were associated with likelihood of returning tests, indicating that population characteristics should be considered when planning events with similar mitigations.

Missing data potentially limited our analyses. Linkage across NHS, public health and participant questionnaire records worked well in most cases, but failed in some, for example with misspelt personal details in questionnaire registration and ticket booking. Sensitivity analyses of the logistic models of variables associated with returning PCR swabs, using multiple imputation, show no substantive differences from the complete case analysis. This analysis assumes that data are missing at random. This assumption may not hold, as it is plausible that missingness may be explained by unmeasured variables. However, this analysis combined with the relatively small levels of missingness in the included variables (all had less than 7.5% missingness, with most under 5%) offers reassurance that our presented analysis does not lead to biased estimates.

Operationally, automatic cancellation of tickets upon linkage of a positive test result was challenging. To deploy this nationally would require a standard protocol for linking ticketholder identity to test results, and for this to be adopted between ticketing and public health agencies. Withdrawal of tickets for positive test results needs to consider not only the most recent result but all positive LFT and PCR results within a reasonable window. We found some evidence of ‘gaming’, whereby a recipient of a positive test result would seek a negative result through further testing. Two of these cases were identified, one of whom attended an event. This was an important practical lesson that eagerness to attend an event may override social responsibility to self-isolate. This could be addressed through app-based tickets that become cancelled immutably on any positive test. Although most non-scanned tickets are likely to be from ticketholders who did not attend, we found evidence that a small number of individuals entered events without tickets being scanned, including one person who later tested positive, identified through contact tracing. Although this research used testing centres, we found social media posts encouraging eventgoers to report negative home LFT results without taking the test to have a ‘certificate’ to gain entry. Other ERP events relied on self-reported test results. Developers of testing systems around events should consider further checks, such as AI reading of uploaded, single-use QR coded lateral flow device images.

Few studies have been published investigating SARS-CoV-2 transmission at and around mass cultural events. A randomised trial at a Barcelona^8^ nightclub showed low levels of transmission with concerted risk mitigation, such as Liverpool ERP; however, both studies were conducted at times of low COVID-19 prevalence. The Barcelona study required mask wearing, which is not sustainable, as shown in a similar intervention in the Netherlands.^9^

We found some evidence that people with symptoms may have attended events, including among likely index cases, with one person reporting symptoms the day after the event, with members of their household already symptomatic pre-event. Communications advising people to stay away from events if they had COVID-19 symptoms should have advised them not to attend if feeling unwell for *any* reason, given changing case definitions^24^ and variable perception of relevant symptoms.

We found that 49% of participants were not concerned with infecting others at events, having recently had a negative LFT result. These data were supported by focus groups revealing how people felt at ease following a negative LFT result. Event organisers and public health teams faced balancing reassurance to support event attendance and reinforcement of risk-reducing behaviours. The rapid sale of tickets, questionnaire responses and focus groups indicated general eagerness to attend events, but with inconsistent perceptions of risks and risk-mitigation responsibilities.

Pandemic management around mass gatherings may benefit from building risk communication and prevention information into booking and attendance preparation processes. Pre-event supervised testing is an opportunity to inform eventgoers about risks and mitigations, including post-test probabilities of infectiousness despite a negative test. Assessment of ventilation at venues using CO_2_ monitors may also improve risk-mitigations.

Close partnership between audiences, event organisers, public health services, including real-time, accessible information systems, are key to infection prevention and control around mass gatherings. In pandemics with prolonged restrictions on mass gatherings, as experienced with COVID-19, the economic and social harms from restrictions must be balanced with the benefits of reduced pathogen amplification and acquisition. Optimal risk mitigation needs closer attention to communication and audience-driven processes alongside the time-sensitive nature of pre-event tests and enhanced environmental measures at venues. These lessons apply not only to the COVID-19 pandemic and each variant wave, but also to wider respiratory virus risk mitigation at mass events in an increasingly connected world, where such mitigations are becoming easier to deploy.

## Supplemental Material

sj-pdf-1-jrs-10.1177_01410768231182389 - Supplemental material for COVID-19 risk mitigation in reopening mass cultural events: population-based observational study for the UK Events Research Programme in Liverpool City Region Supplemental material, sj-pdf-1-jrs-10.1177_01410768231182389 for COVID-19 risk mitigation in reopening mass cultural events: population-based observational study for the UK Events Research Programme in Liverpool City Region by Girvan Burnside, Christopher P Cheyne, Gary Leeming, Michael Humann, Alistair Darby, Mark A Green, Alexander Crozier, Simon Maskell, Kay O’Halloran, Elena Musi, Elinor Carmi, Naila Khan, Debra Fisher, Rhiannon Corcoran, Jake Dunning, W John Edmunds, Kukatharmini Tharmaratnam, David M Hughes, Liora Malki-Epshtein, Malcolm Cook, Ben M Roberts, Eileen Gallagher, Kate Howell, Meera Chand, Robin Kemp, Matthew Boulter, Tom Fowler, Malcolm G Semple, Emer Coffey, Matt Ashton, The COVID-19 Genomics UK (COG-UK) Consortium, Marta García-Fiñana and Iain E Buchan in Journal of the Royal Society of Medicine

sj-pdf-2-jrs-10.1177_01410768231182389 - Supplemental material for COVID-19 risk mitigation in reopening mass cultural events: population-based observational study for the UK Events Research Programme in Liverpool City Region Supplemental material, sj-pdf-2-jrs-10.1177_01410768231182389 for COVID-19 risk mitigation in reopening mass cultural events: population-based observational study for the UK Events Research Programme in Liverpool City Region by Girvan Burnside, Christopher P Cheyne, Gary Leeming, Michael Humann, Alistair Darby, Mark A Green, Alexander Crozier, Simon Maskell, Kay O’Halloran, Elena Musi, Elinor Carmi, Naila Khan, Debra Fisher, Rhiannon Corcoran, Jake Dunning, W John Edmunds, Kukatharmini Tharmaratnam, David M Hughes, Liora Malki-Epshtein, Malcolm Cook, Ben M Roberts, Eileen Gallagher, Kate Howell, Meera Chand, Robin Kemp, Matthew Boulter, Tom Fowler, Malcolm G Semple, Emer Coffey, Matt Ashton, The COVID-19 Genomics UK (COG-UK) Consortium, Marta García-Fiñana and Iain E Buchan in Journal of the Royal Society of Medicine

sj-pdf-3-jrs-10.1177_01410768231182389 - Supplemental material for COVID-19 risk mitigation in reopening mass cultural events: population-based observational study for the UK Events Research Programme in Liverpool City Region Supplemental material, sj-pdf-3-jrs-10.1177_01410768231182389 for COVID-19 risk mitigation in reopening mass cultural events: population-based observational study for the UK Events Research Programme in Liverpool City Region by Girvan Burnside, Christopher P Cheyne, Gary Leeming, Michael Humann, Alistair Darby, Mark A Green, Alexander Crozier, Simon Maskell, Kay O’Halloran, Elena Musi, Elinor Carmi, Naila Khan, Debra Fisher, Rhiannon Corcoran, Jake Dunning, W John Edmunds, Kukatharmini Tharmaratnam, David M Hughes, Liora Malki-Epshtein, Malcolm Cook, Ben M Roberts, Eileen Gallagher, Kate Howell, Meera Chand, Robin Kemp, Matthew Boulter, Tom Fowler, Malcolm G Semple, Emer Coffey, Matt Ashton, The COVID-19 Genomics UK (COG-UK) Consortium, Marta García-Fiñana and Iain E Buchan in Journal of the Royal Society of Medicine

sj-pdf-4-jrs-10.1177_01410768231182389 - Supplemental material for COVID-19 risk mitigation in reopening mass cultural events: population-based observational study for the UK Events Research Programme in Liverpool City Region Supplemental material, sj-pdf-4-jrs-10.1177_01410768231182389 for COVID-19 risk mitigation in reopening mass cultural events: population-based observational study for the UK Events Research Programme in Liverpool City Region by Girvan Burnside, Christopher P Cheyne, Gary Leeming, Michael Humann, Alistair Darby, Mark A Green, Alexander Crozier, Simon Maskell, Kay O’Halloran, Elena Musi, Elinor Carmi, Naila Khan, Debra Fisher, Rhiannon Corcoran, Jake Dunning, W John Edmunds, Kukatharmini Tharmaratnam, David M Hughes, Liora Malki-Epshtein, Malcolm Cook, Ben M Roberts, Eileen Gallagher, Kate Howell, Meera Chand, Robin Kemp, Matthew Boulter, Tom Fowler, Malcolm G Semple, Emer Coffey, Matt Ashton, The COVID-19 Genomics UK (COG-UK) Consortium, Marta García-Fiñana and Iain E Buchan in Journal of the Royal Society of Medicine

sj-pdf-5-jrs-10.1177_01410768231182389 - Supplemental material for COVID-19 risk mitigation in reopening mass cultural events: population-based observational study for the UK Events Research Programme in Liverpool City Region Supplemental material, sj-pdf-5-jrs-10.1177_01410768231182389 for COVID-19 risk mitigation in reopening mass cultural events: population-based observational study for the UK Events Research Programme in Liverpool City Region by Girvan Burnside, Christopher P Cheyne, Gary Leeming, Michael Humann, Alistair Darby, Mark A Green, Alexander Crozier, Simon Maskell, Kay O’Halloran, Elena Musi, Elinor Carmi, Naila Khan, Debra Fisher, Rhiannon Corcoran, Jake Dunning, W John Edmunds, Kukatharmini Tharmaratnam, David M Hughes, Liora Malki-Epshtein, Malcolm Cook, Ben M Roberts, Eileen Gallagher, Kate Howell, Meera Chand, Robin Kemp, Matthew Boulter, Tom Fowler, Malcolm G Semple, Emer Coffey, Matt Ashton, The COVID-19 Genomics UK (COG-UK) Consortium, Marta García-Fiñana and Iain E Buchan in Journal of the Royal Society of Medicine

sj-pdf-6-jrs-10.1177_01410768231182389 - Supplemental material for COVID-19 risk mitigation in reopening mass cultural events: population-based observational study for the UK Events Research Programme in Liverpool City Region Supplemental material, sj-pdf-6-jrs-10.1177_01410768231182389 for COVID-19 risk mitigation in reopening mass cultural events: population-based observational study for the UK Events Research Programme in Liverpool City Region by Girvan Burnside, Christopher P Cheyne, Gary Leeming, Michael Humann, Alistair Darby, Mark A Green, Alexander Crozier, Simon Maskell, Kay O’Halloran, Elena Musi, Elinor Carmi, Naila Khan, Debra Fisher, Rhiannon Corcoran, Jake Dunning, W John Edmunds, Kukatharmini Tharmaratnam, David M Hughes, Liora Malki-Epshtein, Malcolm Cook, Ben M Roberts, Eileen Gallagher, Kate Howell, Meera Chand, Robin Kemp, Matthew Boulter, Tom Fowler, Malcolm G Semple, Emer Coffey, Matt Ashton, The COVID-19 Genomics UK (COG-UK) ConsortiumMarta García-Fiñana and Iain E Buchan in Journal of the Royal Society of Medicine
