# Supplementary material for: COVID-19 risk mitigation in reopening mass cultural events: population-based observational study for the UK Events Research Programme in Liverpool City Region
Source: J R Soc Med. 2023 Jun 23;117(1):11–23. doi: 10.1177/01410768231182389 (PMC10858718; doi:10.1177/01410768231182389)

Figure S1: Pre-event flow diagram for the business festival

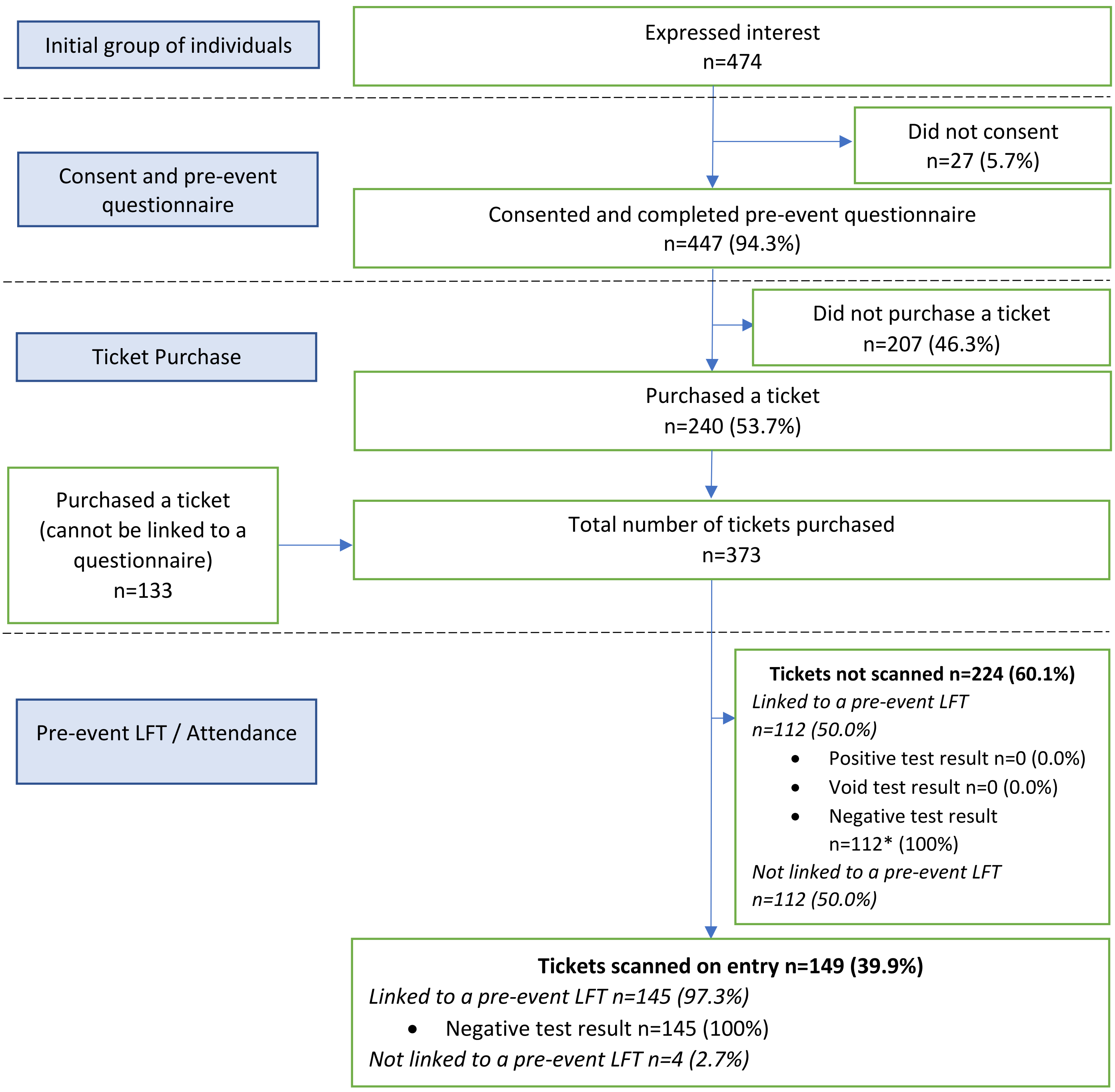

* 1 of these 112 was preceded by a void LFT.

Figure S2: Post-event flow diagram for the business festival

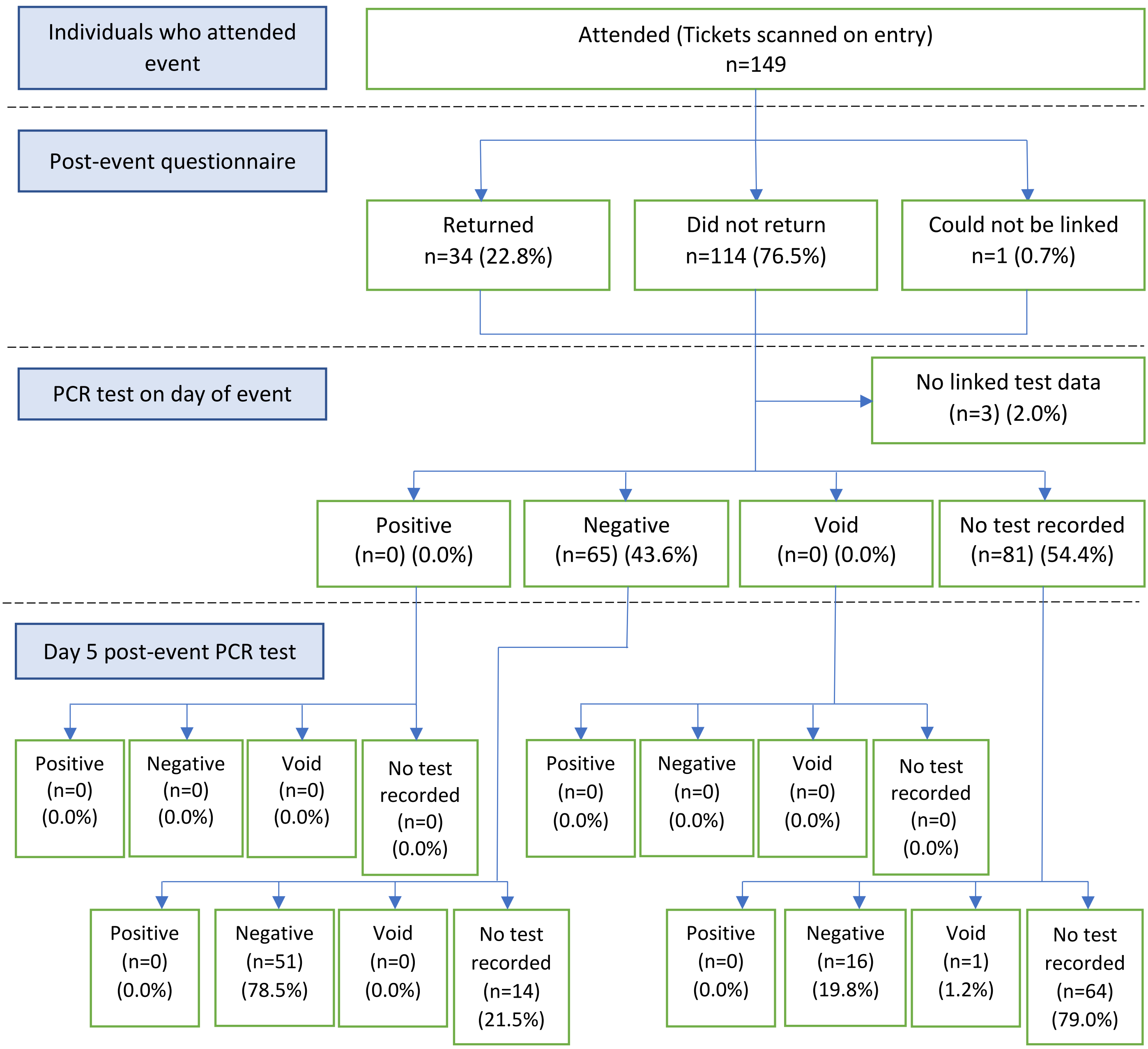

Figure S3: Pre-event flow diagram for the nightclub events.

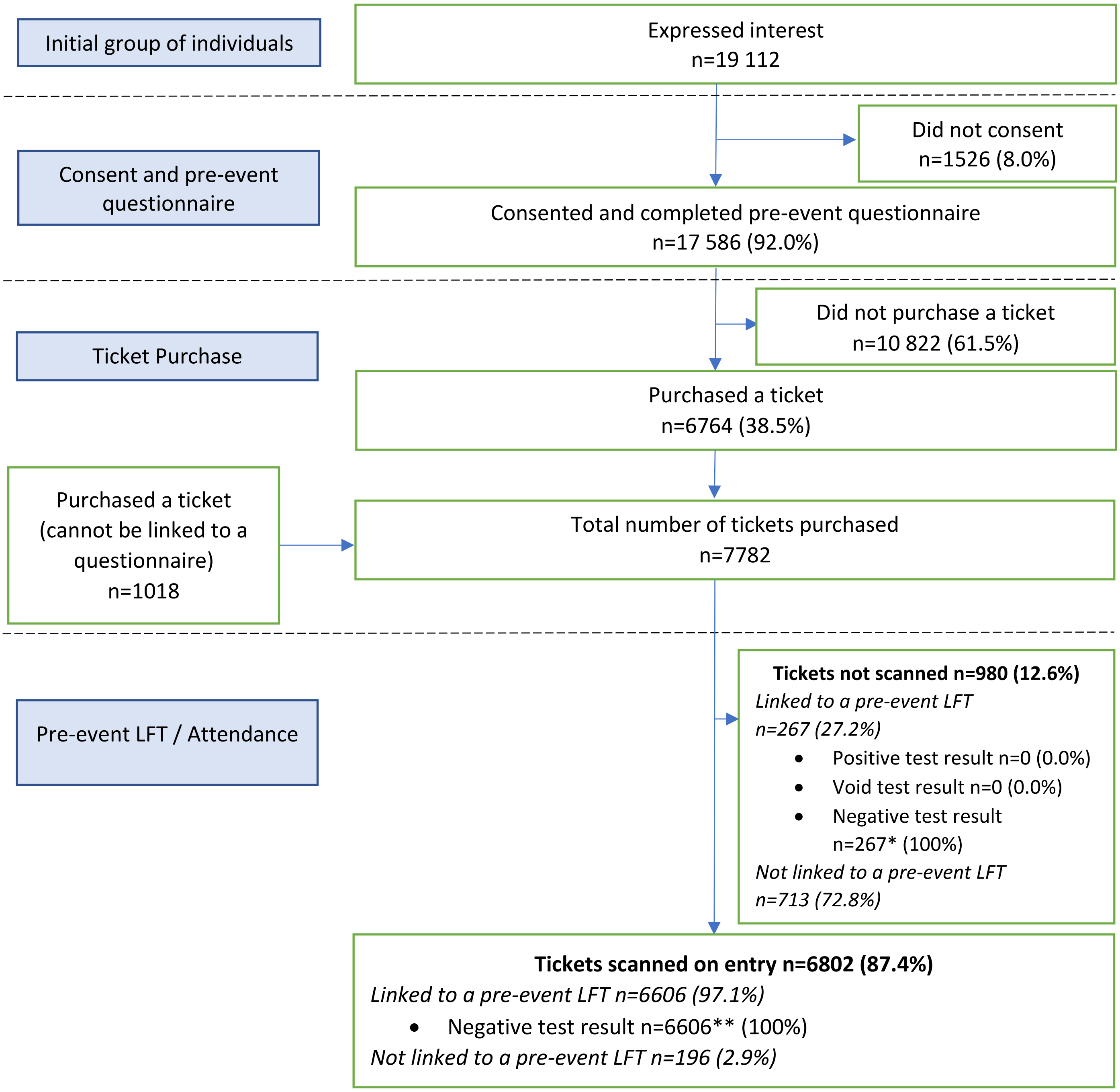

*1 of these 267 was preceded by a positive LFT.
** 15 of these 6606 were preceded by a void LFT.

Figure S4: Post-event flow diagram for the nightclub events.

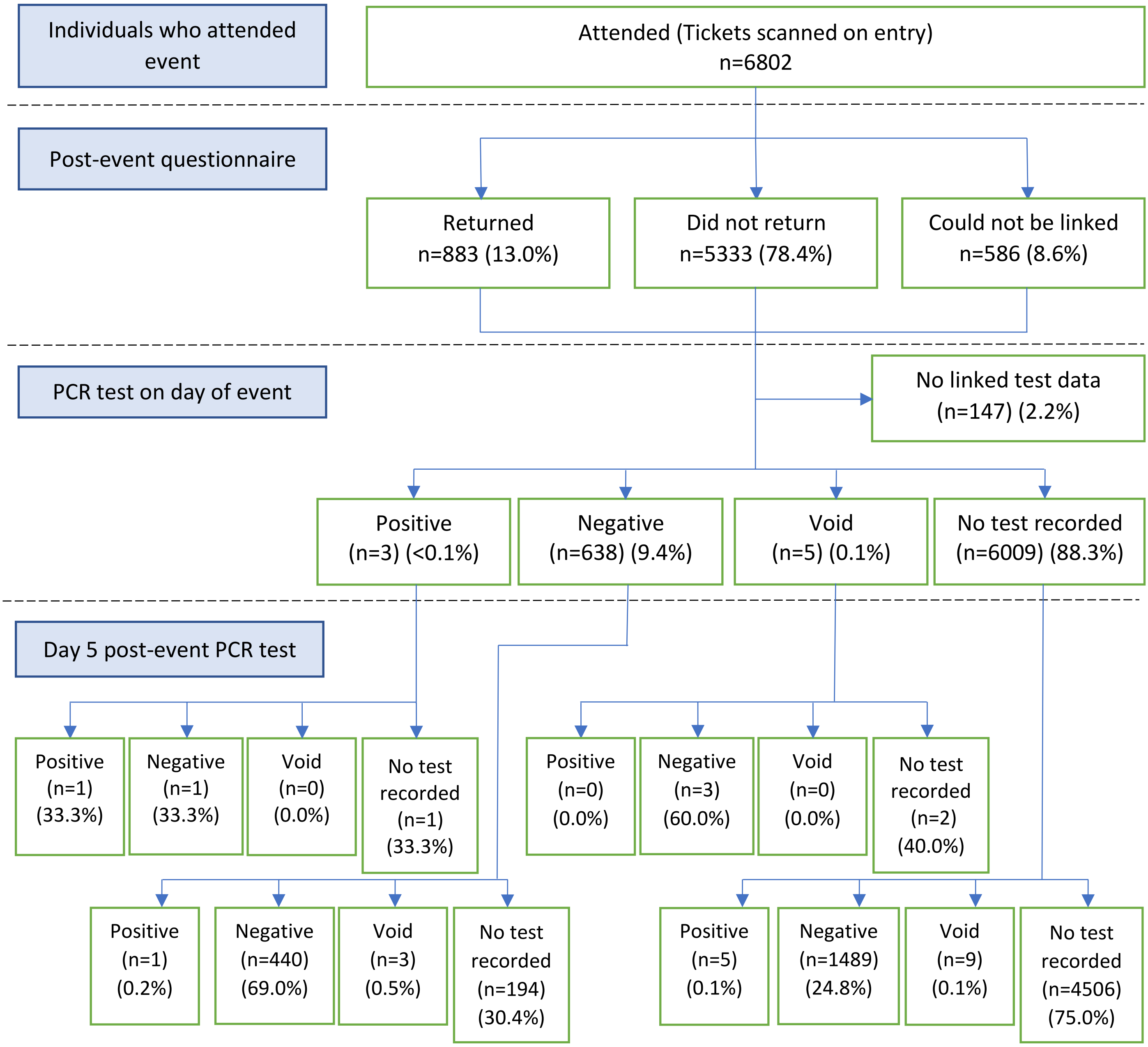

Figure S5: Pre-event flow diagram for the music festival

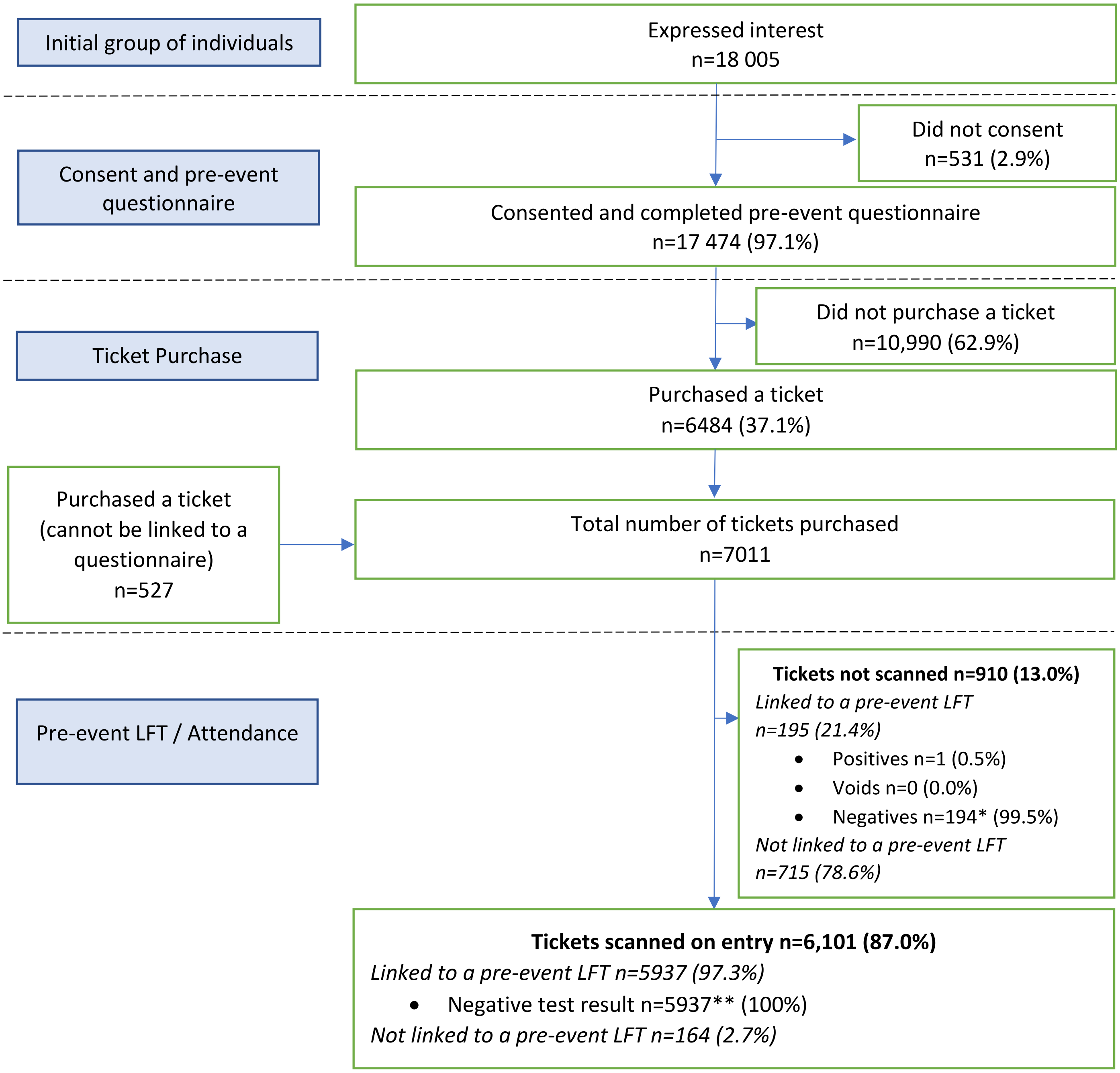

*2 of these 194 were preceded by a positive LFT (one of which also had a void LFT).
**1 of these 5937was preceded by a positive LFT and 9 were preceded by a void LFT.

Figure S6: Post-event flow diagram for the music festival

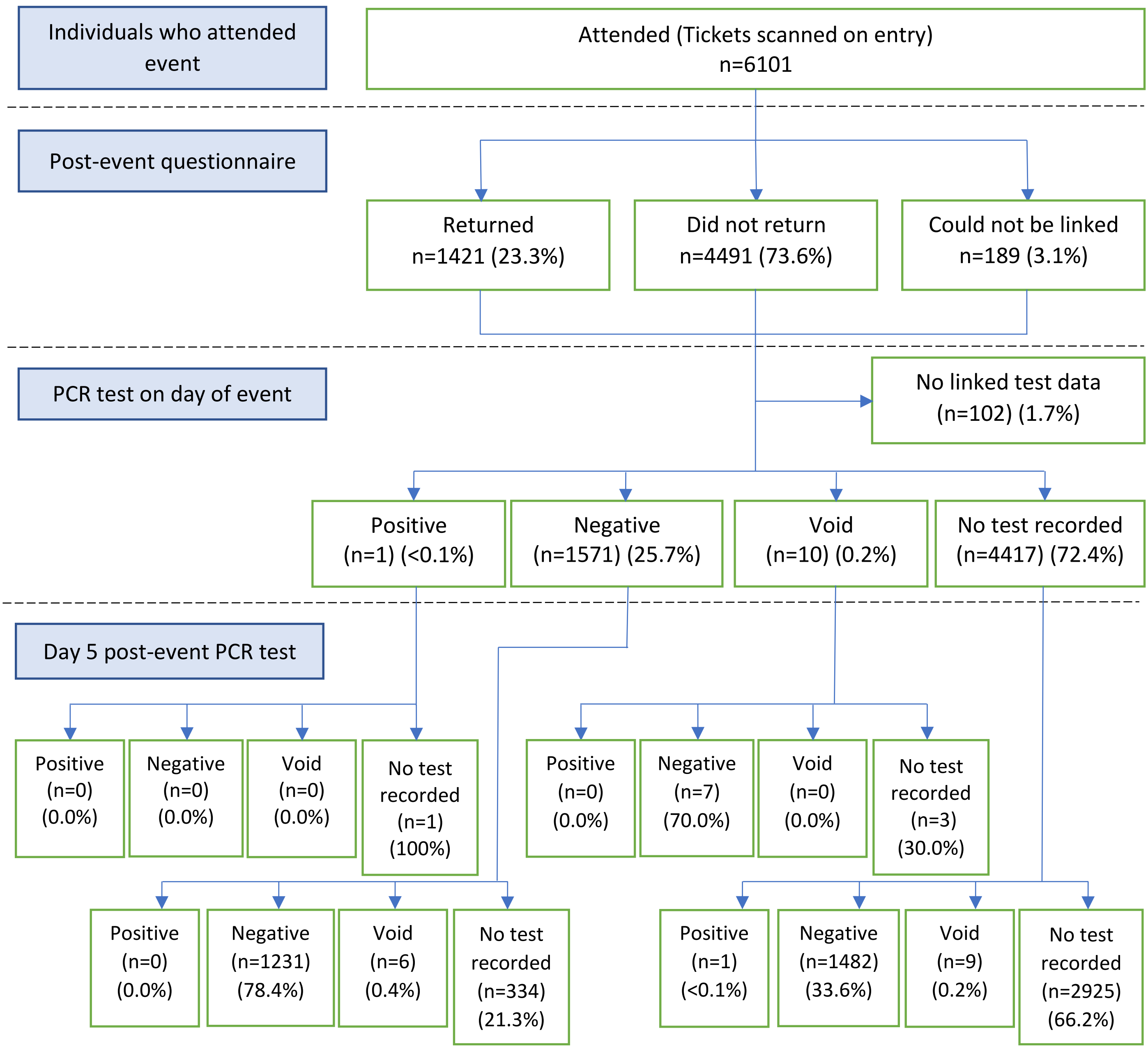

Supplement: sj-pdf-1-jrs-10.1177_01410768231182389 - Supplemental material for COVID-19 risk mitigation in reopening mass cultural events: population-based observational study for the UK Events Research Programme in Liverpool City Region [file sj-pdf-1-jrs-10.1177_01410768231182389.pdf]
