# Supplementary material for: COVID-19 risk mitigation in reopening mass cultural events: population-based observational study for the UK Events Research Programme in Liverpool City Region
Source: J R Soc Med. 2023 Jun 23;117(1):11–23. doi: 10.1177/01410768231182389 (PMC10858718; doi:10.1177/01410768231182389)
Supplement: sj-pdf-2-jrs-10.1177_01410768231182389 - Supplemental material for COVID-19 risk mitigation in reopening mass cultural events: population-based observational study for the UK Events Research Programme in Liverpool City Region [file sj-pdf-2-jrs-10.1177_01410768231182389.pdf]

Supplementary Methods

Detailed investigation of case type

Case classification (index, primary, secondary, unrelated) was determined by consensus between three independent experts. Recognising that individuals can remain RT-PCR positive for some time after the infectious period (positivity often lasts for a median of 22-23 days while the infectious period in non-immunocompromised individuals is normally 5-10 days[13 14]) and that viral load, which correlates with infectiousness, normally peaks in the days surrounding symptom onset, we sought to piece together this information to gauge whether RT-PCR positive cases were infectious at the time of the event or not.

We estimated, with differing degrees of confidence, whether cases were infectious at the time of the event (index case), infected at the event (primary case), infected by someone who became infected related to the event (secondary case), or were infected prior to the event but no longer infectious/were infected completely unrelatedly to the event (unrelated). Information used for this was LFT test status, CT values of the RT-PCR test at each time-point (an indicator of viral load), symptom status, and the published literature on the average incubation and infectious period for people infected with SARS-CoV-2.

Logistic regression methods

Multiple logistic regression was used to identify factors associated with the likelihood of returning a PCR test within 7 days after event. The date of PCR return was assumed to be the day before the specimen was processed. The purpose of the logistic models were to explore the associations of a range of variables with the outcome (and not the causal effect of a primary exposure of interest). Covariates included in the model were age (in years), sex, ethnicity, index of multiple deprivation quintile, vaccination status (none, first, second), previous COVID infection in 2021 (based on recorded positive tests) and concern about infecting others. Models were fitted separately for each event, and overall in combined models, adding event as an additional variable. Participants who attended multiple events were only included once in the combined model. Backward variable selection was carried out using the Akaike method. A sensitivity analysis was conducted exploring the effect of missing data (under the missing at random assumption) based on a model with multiple imputed values by chained equations (using 10 imputations and 50 iterations). Statistical analyses were carried out in R (version 3.6.1 or later) and reproduced by a second statistician.

Analysis of CO_2 levels

The concentration of carbon dioxide (CO_2) in indoor air was measured using sensors affixed to walls in the monitored venues. CO_2 is exhaled in breath, and although not a direct quantitative indicator of airborne disease transmission risk, it is an effective proxy for occupancy relative to the amount of ventilation which can indicate the risk of long-range aerosol transmission indoors[1]. Following the recommendations of the Scientific Advisory Group for Emergencies-Environmental Modelling Group (SAGE-EMG) and the Independent Scientific Pandemic Insights Group on Behaviours (SPI-B), the UK Government has made a series of recommendations for target CO_2 concentrations indoors[2,3]. To reduce the transmission risk of SARS-CoV-2, indoor spaces with CO_2 concentrations regularly exceeding 1500 ppm indicate poor ventilation and are a priority for improvement. CO_2 concentrations consistently below 800 ppm during occupancy indicate that the space is well ventilated. Spaces where aerosol generating activities occur (e.g. singing, aerobic activity, or dancing) should ensure sufficient ventilation to maintain CO_2 concentration below 800 ppm.

Monitoring of indoor carbon dioxide (CO_2) concentration took place in two of the Liverpool City Region pilot events: The Good Business Festival (one day) using 51 CO_2 sensors and Circus Nightclub (two consecutive days) using 33 CO_2 sensors. The sensors were distributed throughout the space in order to detect the detailed distribution of air quality around the space and to assess ventilation effectiveness and crowding throughout.

Focus groups

In total, 366 event attendees were contacted with a view to seeking their involvement in post-event focus groups. Those contacted had indicated a willingness to be approached again by the research team and were selected based on demographic spread of each event attendee list. Eight focus groups, held between 1 and 3 weeks post-event were carried out. Two groups were conducted with conference attendees, two with nightclub attendees, and four with the outdoor music concert attendees. These comprised a total of 31 participants (11 male, 20 female, ages 18 to 71 years). The aim was to gather lived experience data of the perceived risks associated with attendance.

---

[1] EMG and SPI-B: Application of CO2 monitoring as an approach to managing ventilation to mitigate SARS-CoV-2 transmission, 27 May 2021 - GOV.UK (www.gov.uk)

[2] Application of CO2 monitoring as an approach to managing ventilation to mitigate SARS-CoV-2 transmission, 27 May 2021 - GOV.UK (www.gov.uk)

[3] EMG: Role of ventilation in controlling SARS-CoV-2 transmission, 30 September 2020 https://www.gov.uk/government/publications/emg-role-of-ventilation-in-controlling-sars-cov-2-transmission-30- september-2020

Digital and social media analysis

Online media reports and social media posts from 15 April to 15 June 2021 were collected and analysed to understand the ways in which the live events programme was communicated via different agencies and media sources, and the public reactions to the programme as revealed through public comments to news media articles, and social media. The data sources include: (1) UK Government and Liverpool City Council webpages[i]; (2) online news media (local and national)[ii]; (3) public comments to online articles and TikTok videos[iii]; and (4) public tweets[iv] and Liverpool City Council tweets[v]. Various search criteria were used to collect and analyse the online news articles[vi] and Twitter data[vii] using the Multimodal Analysis Platform (MAP) (O'Halloran Pal and Jin 2021). Natural Language Processing (NLP)[viii] were used to identify the topics which were discussed, key terms, and the perceived risks and concerns. The sentiment analysis was conducted using Google's BERT model[ix] (Hoang, Bihorac and Roucesthrough 2019). In addition, qualitative analysis was undertaken for the public tweets and Circus event articles to investigate the concerns in more detail.

---

[i] Sources: https://liverpool.gov.uk; https://www.cdc.gov/coronavirus/2019ncov/index.html; https://www.ecdc.europa.eu; https://www.gov.uk/coronavirus; https://www.gov.uk/government/organisations/scientific-advisory-group-for-emergencies; https://www.independentsage.org; https://www.nhs.uk/conditions/coronavirus-covid-19; https://www.who.int/emergencies/diseases/novel-coronavirus-2019

[ii] UK Newspapers: The Guardian, The Independent, The Evening Standard, The Metro, and The Sun and BBC (from 6 June 2021); Local media: Liverpool Echo; Online news media articles about the Circus events provided by the Liverpool City Council.

[iii] Public comments to Circus events and TicTok videos about gaming test results were analysed.

[iv] Public tweets were collected using Twitter API and web scraping.

[v] Tweets posted by Liverpool City Council, Culture Liverpool and Visit Liverpool.

[vi] Newspaper filters:

```
|dedup text | eval Summary=lower(Summary)|where

(!like(Summary,"%prince philip%") and !like(Summary,"%airport%") and
!like(Summary,"%pilotage%"))

and

((like(Summary,"%circus%") and like(Summary,"%liverpool%") and like(Summary,"%pilot%"))

or

(like(Summary,"%liverpool%") and like(Summary,"%pilot%") and like(Summary,"%event%"))
```

or

(like(Summary,"%liverpool%") and like(Summary,"%gig%") and like(Summary,"%pilot%"))

or

(like(Summary,"%ERP%") and like(Summary,"%pilot%"))

or

(like(Summary,"%postcovid%") and like(Summary,"%concert%"))

or

(like(Summary,"%circus%") and like(Summary,"%nighclub%") and like(Summary,"%pilot%"))

or

(like(Summary,"%liverpool%") and like(Summary,"%trial%") and like(Summary,"%gigs%"))

or

(like(Summary,"%liverpool%") and like(Summary,"%test%") and like(Summary,"%event%") and like(Summary,"%research%"))

or

(like(Summary,"%liverpool%") and like(Summary,"%gig%") and like(Summary,"%research%"))

or

(like(Summary,"%sefton park%") and like(Summary,"%festival%")))| table *

[vii] Twitter filters:
|eval text=lower(text)|where

(!like(Summary,"%prince philip%") and !like(Summary,"%airport%") and !like(Summary,"%pilotage%"))

and

((like(text,"%sefton%") and (like(text,"%music%") OR like(text,"%live%") OR like(text,"%trial%") OR like(text,"%research%") OR like(text,"%gig%") OR like(text,"%thousands%") OR like(text,"%maskfree%") OR like(text,"%event%")))

OR

(like(text,"%liverpool%") and like(text,"%music%") and (like(text,"%live%") OR like(text,"%trial%") OR like(text,"%research%") OR like(text,"%gig%") OR like(text,"%thousands%") OR like(text,"%maskfree%") OR like(text,"%event%")))

OR

(like(text,"%circus%") and like(text,"%liverpool%"))

or

(like(text,"%liverpool%") and like(text,"%trial%"))

or

(like(text,"%liverpool%") and like(text,"%pilot%"))

or

(like(text,"%liverpool%") and like(text,"%gig%"))

and

(like(Summary,"%circus%") and like(Summary,"%liverpool%") and like(Summary,"%pilot%"))

or

(like(Summary,"%liverpool%") and like(Summary,"%pilot%") and like(Summary,"%event%"))

or

(like(Summary,"%liverpool%") and like(Summary,"%gig%") and like(Summary,"%pilot%"))

or

(like(Summary,"%ERP%") and like(Summary,"%pilot%"))

or

(like(Summary,"%postcovid%") and like(Summary,"%concert%"))

or

(like(Summary,"%circus%") and like(Summary,"%nighclub%") and like(Summary,"%pilot%"))

or

(like(Summary,"%liverpool%") and like(Summary,"%trial%") and like(Summary,"%gigs%"))

or

(like(Summary,"%liverpool%") and like(Summary,"%test%") and like(Summary,"%event%") and like(Summary,"%research%"))

or

(like(Summary,"%liverpool%") and like(Summary,"%gig%") and like(Summary,"%research%"))

or

(like(Summary,"%sefton park%") and like(Summary,"%festival%"))

)

|dedup text, screen_name

[viii] Python Natural Language Toolkit (NLTK): https://realpython.com/nltk-nlp-python/

[ix] BERT sentiment analysis: https://github.com/google-research/bert
