# Supplementary material for: COVID-19 risk mitigation in reopening mass cultural events: population-based observational study for the UK Events Research Programme in Liverpool City Region
Source: J R Soc Med. 2023 Jun 23;117(1):11–23. doi: 10.1177/01410768231182389 (PMC10858718; doi:10.1177/01410768231182389)

Figure S3.1. Liverpool map showing the areas the attendees came from for each event, with attendance clustered in student areas and areas surrounding the Universities/city centre. Risk of transmission and SARS-CoV-2 cases

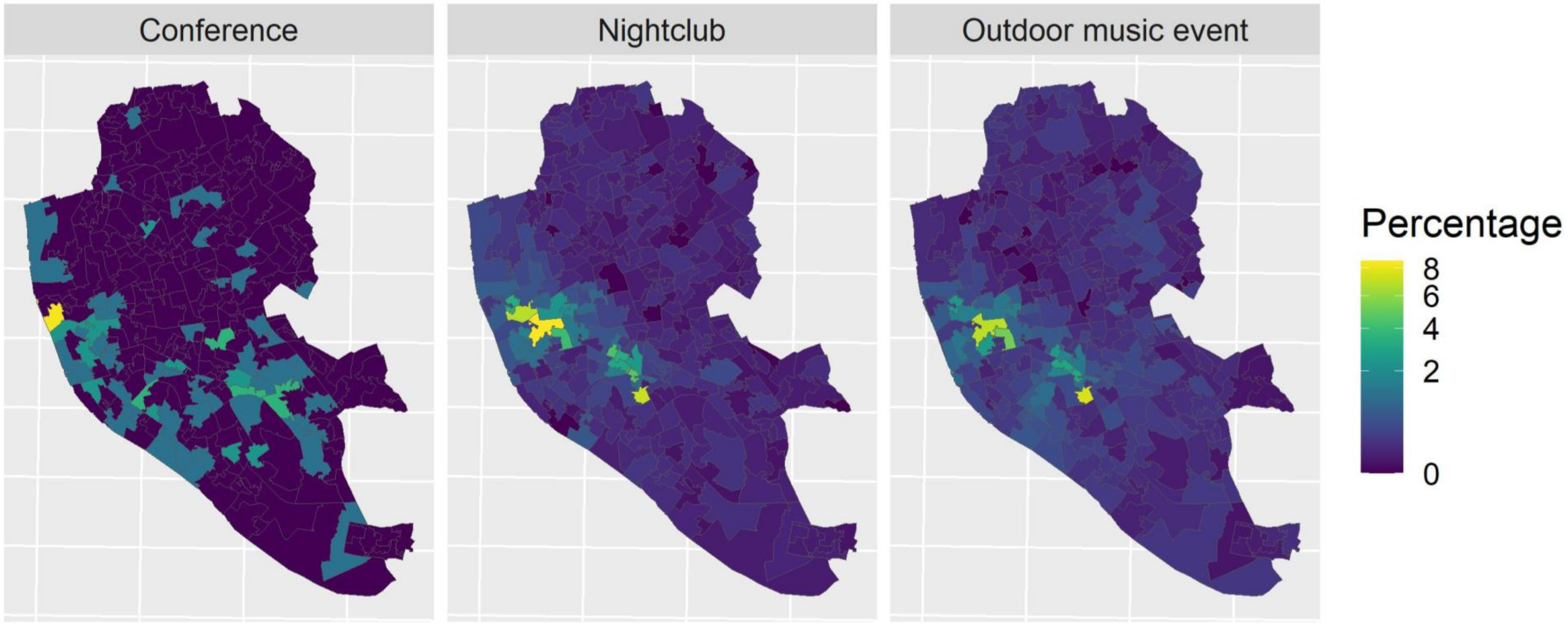

Table S3.1: **Positive tests identified from the attendees. Cases are classified as a *low-index case* (attendee who arrived at the event with a relatively low viral load and was somewhat likely infectious and may have infected others), *high-index case* (attendee who arrived at the event with a high viral load (eg a CT value of less than 25), was likely infectious and may have infected others), p*rimary case* (infected by index case at the event), *secondary case* (infected by primary case after the event), and *unrelated case* (attended the event and previously/now positive, but (likely) not infected at the event or by anyone who attended the event). Where the case type is ambiguous confidence is articulated as (H)igh, (M)edium and (L)ow. Groups of friends identified in a cluster of size 2 (orange) and s cluster of size 4 (green). Day of PCR estimated as day before specimen processing date.**

**Event**	**Case number (as in Table 2 in paper)**	**LFT Pre-event**	**PCR day 0-4**						**PCR post-event day 4-7**						Case type
			**result**	**time**	**Ct**	**ORf1ab**	**N**	**S**	**result**	**time**	**Ct**	**ORf1ab**	**N**	**S**	
Sefton Park music festival (2nd May)	1	-ve	+ve	-18	25	25	25	-							Low-Index (H)
			+ve	3	32	33	31	-							
	5	-ve							+ve	6	33	-	33	-	High-Index (L) or secondary (M)[1]
	13	+/-ve	-	-	-	-	-	-	-	-	-	-	-	-	High-Index (H)[2]
	14	-ve	-	-	-	-	-	-	-	-	-	-	-	-	High-Index (H)[3]
Circus Nightclub night 1 (30th April)	2	-ve	+ve	0	33	-	33	-	-ve	5					Unrelated(H)[4]
			-ve	1											
	3	-ve	+ve	0	21	21	21	-							High-Index (H)
	6	-ve	-ve	0					+ve	5	26	25	27	26	Primary (H)
	7	-ve							+ve	5	32	31	34	-	High-Index (L) or secondary (M)[1]
	8	NA							+ve	5	20	19	20	20	Primary (H)[5]

Circus nightclub night 2 (1st May)	4	-ve	+ve	2	22	21	22	-	+ve	7	29	29	29	-	Low-Index (H)
									+ve	9[6]	NA	NA	NA	NA	
									+ve	16[6]	NA	NA	NA	NA	
	9	-ve							+ve	5	24	24	25	-	Secondary (M) or unrelated (M)[7]
									+ve	7	19	19	19	-	
	10	-ve							+ve	7	18	18	18	-	Primary (H)
									+ve	9	15	15	15	-	
	11	-ve							+ve	5	13	13	13	-	Primary (H)
	12	-ve							+ve	7	15	15	15	-	Primary (H)
	15	-ve	-	-	-	-	-	-	-	-	-	-	-	-	Low-Index (M)[8]

[1] Were viral load falling at the time of the PCR test (such that the individual was an index case), the LFT would have been likely to be positive.
[2] Ticketholder had a positive LFT pre-event followed by a negative LFT, both taken the day before the event
[3] Ticketholder had a positive LFT one day post-event (not confirmed by PCR)
[4] The negative LFT after the event implies that the viral load was likely to be falling at the time of the event and the individual was unlikely to be infectious at that time.
[5] No matching LFT found, this attendee was not scanned as attending, but reported that they did attend. Peak viral load approximately 5 days after event implies that the individual was a primary case.
[6] Day 9 and day 16 tests were Pillar 1 matched from PHE data, with no Ct values available.
[7] Difficult to distinguish between a secondary and unrelated case without information about the contact network.
[8] Ticketholder had a positive PCR 8 days before the event, followed by a negative LFT pre-event and no PCR return afterwards

Table S3.2: PCRs from attendees testing positive from 30 days pre-event[1]

Event	Row ID	Pre-event LFT	PCR result	Time PCR (days pre-event)	Ct (mean)	ORf1ab	N gene	S gene
Sefton	1	-ve	+ve	18	23	23	23	-
	2	-ve	+ve	16	16	16	16	-
	3[2]	-ve	+ve	18	25	25	25	-
Circus night 1	4	-ve	+ve	28	32	31	32	-
	5	-ve	+ve	19	18	18	18	-
Circus night 2	6	-ve	+ve	30	32	32	32	-
	7	-ve	+ve	28	21	21	21	-
	8	-ve	+ve	16	25	25	26	-
	9	-ve	+ve	16	29	29	29	-
	10	-ve	+ve	8	13	13	14	-

[1] Day of PCR estimated as day before specimen processing date

[2] This attendee also tested positive in the pre-event window and is included in Table 3 (row 1).

Table S3.3: Positive LFTs pre-event from attendees (<1 week prior to event)

Event	Row #	Day of LFT (days before event)	Time of LFT (hours before event)	Confirmatory PCR	Day of PCR (days before event) [1]	Ct (mean)	ORf1 ab	N gene	S gene	Status	Scanned into event
Sefton Park	1	3	82	-ve	2	NA				False LFT positive	Yes
	2	1	32	NA (a second LFT was negative)		NA				Unverified LFT positive	Yes
	3	1	31	-ve	1	NA				False LFT positive	No
	4	1	31 and 27[2]	+ve	0	21	21	22	NA	True LFT positive	No
	5	1	30	NA (a second LFT was negative)		NA				Unverified LFT positive	No
Circus night 1	6	1	28	+ve	0	19	20	19	NA	True LFT positive	No

[1] Day of PCR estimated as day before specimen processing date
[2] Ticketholder had two positive LFTs the day before the event

Table S3.4: Attendees with positive LFTs within a week after attending the event

Event	ID	Day of positive LFT result	Day of PCR results	PCR result	Status
Sefton Park	1	7	6 and 13	-ve	False positive
	2	5	7	-ve	False positive
	3	3	6	-ve	False positive
	4	1	NA	None	Unverified LFT positive
Circus night 1	5 (row 8 in table 3)	5	5	+ve	True positive
Circus night 2	6	4	7	-ve	False positive
	7	2	4	-ve	False positive
	8 (row 10 in table 3)	6	5 and 7	+ve	True Positive

Table S3.5: Attendees with a first positive PCR test recorded after 1 week post-event

Event	Row ID	Pre-event LFT	PCR d-1 to d3	PCR d4 to d7	PCR d8 to d16	Day of +ve LFT	Day of +ve PCR	Mean Ct
Good Business	1	Negative	None	None	None	.	35	17
Circus Night 1	2	Negative	None	None	None	27	26	18
	3	Negative	None	None	None	39		
	4	Negative	None	Negative	None	39		
	5	Negative	None	None	Negative	40	40	13
	6	Negative	None	None	Negative	37	36	17
	7	Negative	None	None	Negative	39	40	21
	8	Negative	None	None	None	41		
	9	Negative	None	Negative	None	.	39	Not available
	10	Negative	None	Negative	None	40		
	11	Negative	None	Negative	None	40		
	12	Negative	None	Negative	None	40		
	13	Negative	None	None	None	36	36	16
	14	Negative	None	None	None	.	39	19
	15	Negative	None	None	None	.	35	32
						.	38	19
						.	39	32
Circus Night 2	16	Negative	None	Negative	None	30	31	15
	17	Negative	None	None	None	.	31	34
	18	Negative	None	None	None	.	36	25
	19	Negative	None	None	None	.	33	19
	20	Negative	None	Negative	None	.	33	15
	21	Negative	None	Negative	None	.	36	16
	22	Negative	None	Negative	None	38	38	15
	23	Negative	None	None	None	34	33	Not available
	24	Negative	None	Negative	None	.	39	20
	25	Negative	None	Negative	None	36	37	18
	26	Negative	None	None	None	.	32	Not available
	27	Negative	None	Negative	None	36		
	28	Negative	None	Negative	None	40		
	29	Negative	None	None	None	.	33	31
	30	Negative	None	None	None	.	39	23
	31	Negative	None	None	None	38		
	32	Negative	None	None	Negative	36	35	17
	33	Negative	Negative	Negative	None	.	36	15
	34	Negative	None	None	None	39	38	14
	35	Negative	None	None	None	.	25	Not available
	36	Negative	None	None	None	35	35	16
	37	Negative	None	None	None	37		
Sefton Park		Negative	None	Negative	None	33	33	Not available
	39	Negative	None	None	None	.	37	23
	40	Negative	Negative	None	Negative	32	33	16
	41	Negative	None	None	None	.	35	17

Event	Row ID		Pre-event LFT	PCR d-1 to d3	PCR d4 to d7	PCR d8 to d16	Day of +ve LFT	Day of +ve PCR	Mean Ct
	42		Negative	Negative	Negative	None	.	30	14
	43		Negative	Negative	Negative	None	39		
	44		Negative	Negative	Negative	None	32	32	16
	45		Negative	Negative	Negative	None	36		
	46		Negative	None	Negative	None	35		
	47		Negative	Negative	Negative	None	.	31	17
	48		Negative	None	None	Negative	.	37	23
	49		Negative	None	Negative	None	35	35	13
	50		Negative	None	Negative	None	.	32	15
	51		Negative	None	None	None	33		
	52		Negative	None	None	None	31		
	53		Negative	None	Negative	None	34	35	16
	54		Negative	None	None	None	31		
	55		Negative	None	None	Negative	.	37	17
	56		Negative	Negative	Negative	None	35	35	17
	57		Negative	Negative	Negative	None	30	33	13
	58		Negative	None	Negative	None	38		
	59		Negative	Negative	None	Negative	26	26	17
	60		Negative	None	None	None	.	36	Not available
	61		Negative	None	Negative	None	.	36	20
	62		Negative	Negative	None	None	18		
	63		Negative	None	None	None	.	38	18
	64		Negative	None	Negative	None	35	34	17
	65		Negative	None	Negative	None	.	30	16
	66		Negative	Negative	None	None	32	34	Not available
							33	34	Not available
	67		Negative	Negative	None	None	36	36	19

Table S3.5 Results of the logistic regression model predicting the likelihood of returning a PCR test 7 days post event

Characteristic	Combined n=10 796 (1/0: 4679/6117)			Nightclub n=5787, (1/0: 1956/3831)			Music festival n=5597, (1/0: 2953/2644)			Business festival n=138, (1/0: 80/58)		
	OR	95% CI	p-value	OR	95% CI	p-value	OR	95% CI	p-value	OR	95% CI	p-value
Age (years)												
	**1·05**	**1·04,1·06**	**<0·001**	**1·04**	**1·03,1·06**	**<0·001**	**1·05**	**1·04, 1·06**	**<0·001**	1·01	0·97, 1·05	0·745
Sex												
Female	—	—		—	—		—	—		—	—	
Male	**0·68**	**0·63,0·74**	**<0·001**	**0·65**	**0·58,0·73**	**<0·001**	**0·70**	**0·63, 0·78**	**<0·001**	0·57	0·27, 1·21	0·144
Ethnicity												
White	—	—		—	—		—	—		—	—	
Asian or Asian British	0·85	0·63,1·15	0·284	0·82	0·56,1·19	0·291	0·90	0·58, 1·40	0·654	—	—	
Black or Black British	**0·42**	**0·23,0·77**	**0·005**	**0·49**	**0·25,0·98**	**0·043**	0·28	0·08, 1·02	0·054	—	—	
Mixed ethnicity	0·89	0·69,1·15	0·391	0·90	0·65,1·25	0·547	0·89	0·62, 1·29	0·550	—	—	
Other ethnic group	1·56	0·75,3·24	0·233	1·74	0·73,4·15	0·213	0·80	0·24, 2·61	0·710			
Prefer not to say	**0·70**	**0·58,0·84**	**<0·001**	**0·69**	**0·53,0·90**	**0·006**	**0·70**	**0·54, 0·90**	**0·006**	—	—	
Deprivation quintile												
1 (Most deprived)	0·89	0·72,1·09	0·259	1·04	0·78,1·39	0·805	0·80	0·60,1·08	0·142	0·33	0·08,1·35	0·122
2	0·94	0·82,1·08	0·392	1·04	0·83,1·26	0·823	0·97	0·80,1·17	0·738	**0·33**	**0·11,0·99**	**0·049**
3	0·95	0·82,1·11	0·541	1·04	0·84,1·30	0·696	0·97	0·79,1·18	0·739	**0·19**	**0·06,0·66**	**0·008**
4	0·89	0·76,1·03	0·119	1·01	0·81,1·26	0·907	0·83	0·68,1·02	0·071	0·73	0·22,2·50	0·620
5 (Least deprived)	—	—		—	—		—	—		—	—	
Vaccinated												
No	—	—		—	—		—	—		—	—	
Yes	**1·35**	**1·23,1·48**	**<0·001**	**1·51**	**1·33,1·72**	**<0·001**	**1·26**	**1·12, 1·42**	**<0·001**	2·24	0·87, 5·72	0·093
Had COVID-19 in 2021												
No	—	—		—	—		—	—		—	—	
Yes	**0·82**	**0·68,0·99**	**0·035**	0·90	0·69,1·17	0·426	**0·75**	**0·58, 0·96**	**0·021**	0·64	0·08, 4·89	0·668
Concern at infecting others												
Not at all	—	—		—	—		—	—		—	—	
Some	**1·32**	**1·22,1·43**	**<0·001**	**1·20**	**1·07,1·34**	**0·002**	**1·43**	**1·29, 1·60**	**<0·001**	1·29	0·61, 2·69	0·506
Event												
Nightclub (reference)	—	—										
Business festival	1·00	0·67,1·48	0·995									
Music festival	**1·94**	**1·79, 2·11**	**<0·001**									

OR = Odds Ratio, CI = Confidence Interval

Figure S3.2: Phylogenetic tree including two attendees at Friday nightclub event

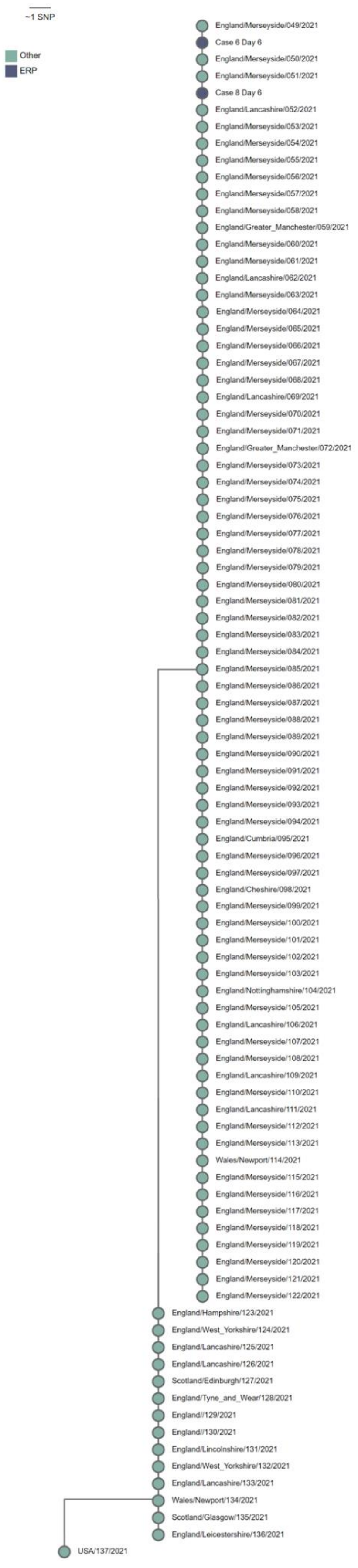

Figure S3.3: Phylogenetic tree including five attendees at Saturday nightclub event

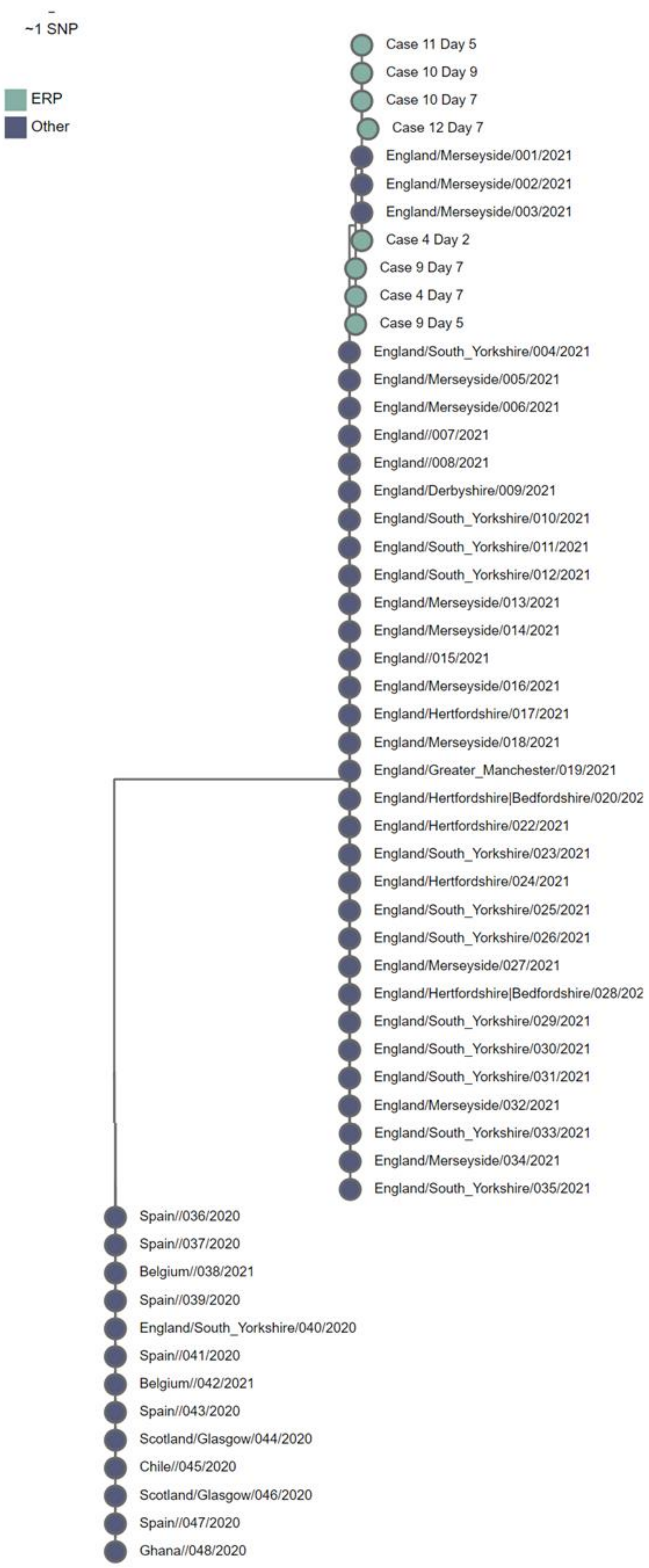

Figure S3.4: Phylogenetic tree including attendee positive 8 days before attending Saturday nightclub event

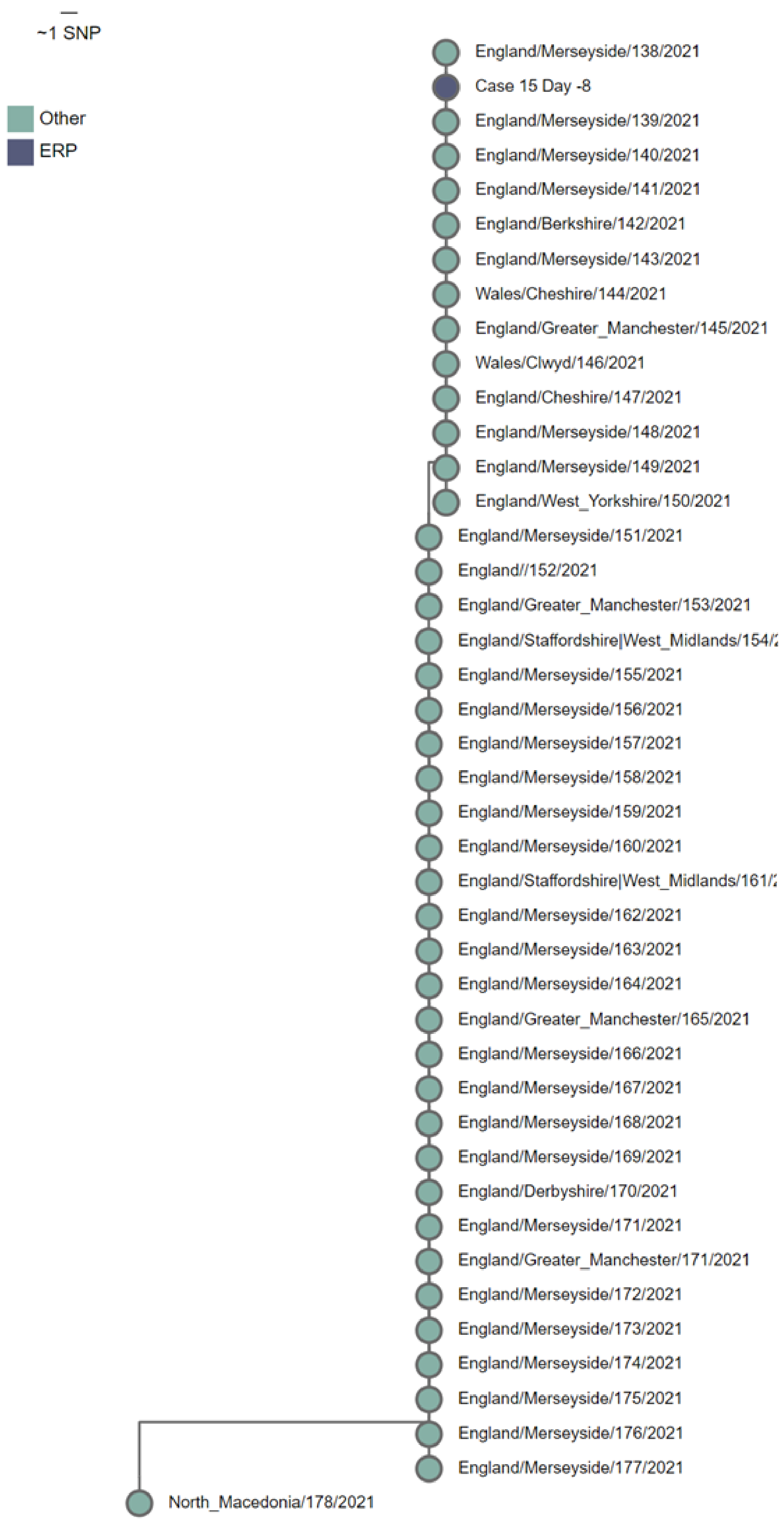

Supplement: sj-pdf-3-jrs-10.1177_01410768231182389 - Supplemental material for COVID-19 risk mitigation in reopening mass cultural events: population-based observational study for the UK Events Research Programme in Liverpool City Region [file sj-pdf-3-jrs-10.1177_01410768231182389.pdf]
