# Supplementary material for: COVID-19 risk mitigation in reopening mass cultural events: population-based observational study for the UK Events Research Programme in Liverpool City Region
Source: J R Soc Med. 2023 Jun 23;117(1):11–23. doi: 10.1177/01410768231182389 (PMC10858718; doi:10.1177/01410768231182389)
Supplement: sj-pdf-4-jrs-10.1177_01410768231182389 - Supplemental material for COVID-19 risk mitigation in reopening mass cultural events: population-based observational study for the UK Events Research Programme in Liverpool City Region [file sj-pdf-4-jrs-10.1177_01410768231182389.pdf]

Analysis of CO_2, crowding and ventilation

The Good Business Festival event was held on 28 April from 14:30 until 18:30. It was hosted in the foyer and in two adjoining auditoria at ACC Liverpool. The building was mechanically ventilated with a design air flow rate of 12 l/s per person for both the foyer and auditoria. Event attendees moved between the foyer and two auditoria at fixed times throughout the event as the customer journey moved from registration (foyer), main talks (main auditorium, capacity 850), drinks receptions (foyer) and a comedy event (drum auditorium, capacity 250). Thus, emission of exhaled CO_2 happened in three main spaces at different times, and so there were periods when the ventilation system was extracting indoor air in unoccupied spaces. Measured CO_2 levels were generally low throughout the event, with a maximum of 779 parts per million (ppm), indicating that at these occupancies the ventilation was sufficient to remove exhaled breath and the associated CO_2 (Figure S4.1).

The Circus Nightclub event was held on two separate days 30 May (Event 1) and 1 June (Event 2) from 14:00 to 23:00. It was hosted in a 34,000 m^3 Victorian-era warehouse at Bramley-Moore Dock. At one end of the warehouse was a bar area. At the other end was a dancefloor and stage. The building was naturally ventilated. There were six very large openings (each 49 m^2) positioned along the southern façade of the building at regular intervals. Three openings were located in the half of the building that contained the bar. These were left fully open throughout the event. But the three openings at the dancefloor end were restricted by lowering shutters to cover half the opening and hanging plastic "butchers screens" to cover the remaining open portion. These screens were intended to prevent noise egress from the venue but had an unintended consequence of reducing the ventilation rate through the openings. The restricted ventilation was combined with overcrowding close to the stage area, resulting in CO_2 levels of over 2000 ppm recorded on both event days in the dancefloor zone close to the stage (Figure S4.2). The CO_2 concentrations in parts of the dancefloor area remained consistently high (i.e. > 1500 ppm) for long periods during the events (5.6 hours during Event 1 and 5.3 hours during Event 2) indicating an increased risk of long-range aerosol transmission.

However, CO_2 concentrations did not reach such high levels further back from the stage and were considerably lower near the bar area where ventilation openings were larger and crowd densities were lower. For example, on the second event day CO_2 remained consistently below 800 ppm in the bar area, indicating a lower risk of long-range aerosol transmission. This is reflected in the greater difference in mean[1] and maximum CO_2 concentrations observed at Circus Nightclub (Figure S4.2)

[1] The mean and maximum values were calculated from all sensors in the nightclub venue.

that is not apparent at the Good Business Festival (Figure S4.1). There are observable similarities between the mean and maximum CO_2 concentrations over both nightclub events, as a result of similar ventilation strategies, occupancies, and crowd densities.

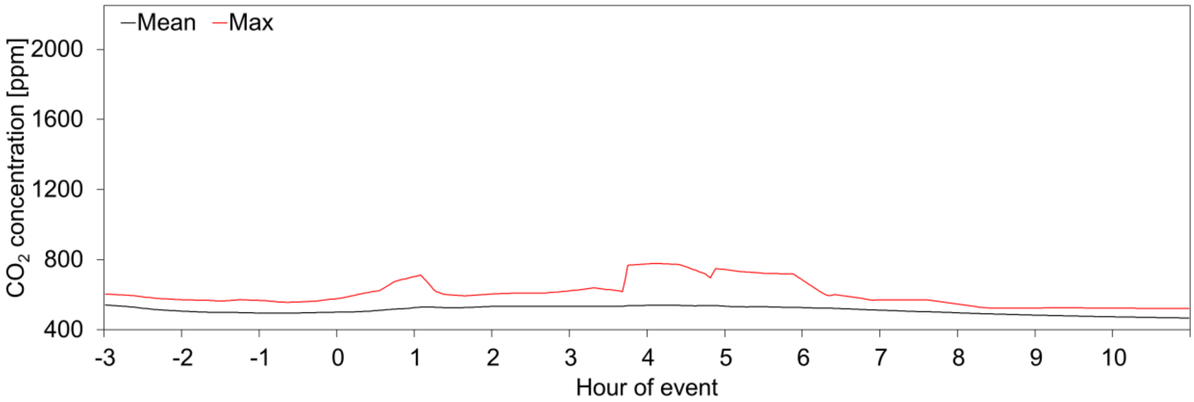

Figure S4.1: Mean and maximum CO_2 concentration measured before, during, and after the ACC Business event. Time "0" is when the conference opening address began.

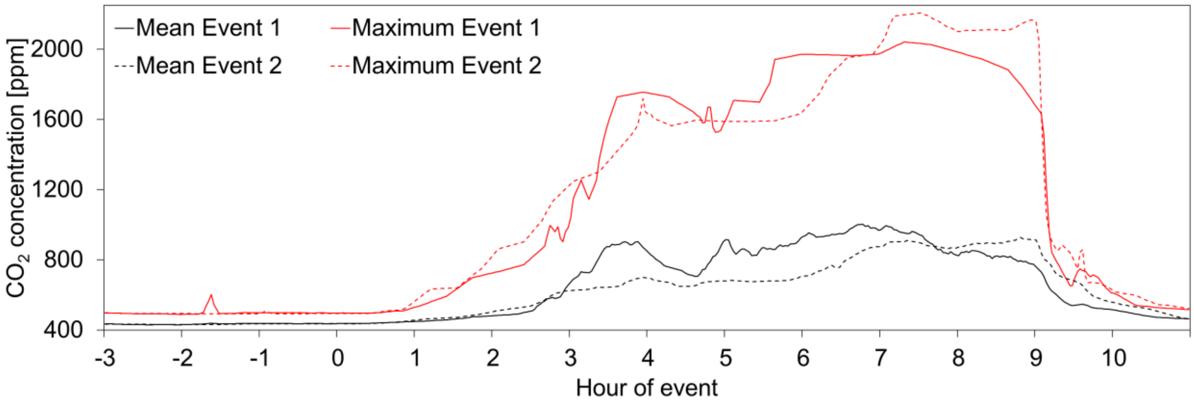

Figure S4.2: Mean and maximum CO_2 concentration measured before, during, and after the Circus Nightclub events at Event 1 and Event 2. Time "0" is when the venue opened to attendees.

Digital and social media analysis

367 media articles from 15 April to 15 June 2021 were examined. These included government and Liverpool City Council media (23 articles), national news media (44 articles), local news media (11 articles) and news media articles specifically about the nightclub events (289 articles). A computational sentiment analysis and a qualitative content analysis showed that the ERP in Liverpool was endorsed and promoted through official channels. The content of articles focused mostly on the entertainment aspects of the events in terms of both narrative and images (e.g. attendees, the crowd, bright lights and the band), with generally high positive sentiment scores. However, an analysis of 4,282 comments posted in response to the media articles found that public responses to the news media about the nightclub events were not always positive. Public reactions were highly polarized, as revealed by computational sentiment scores which ranged from extremely positive to negative values, averaging out at an overall neutral score, as displayed in Figure S4.3). While some people welcomed ERP, others felt it was too early for the government to hold such test events and it was unethical to put people in potential danger. Similarly, an analysis of 2,144 public Tweets including 831 retweets showed a diverse range of responses to the live events taking place and the accompanying publicity, but in this case the average sentiment score was positive, as displayed in Figure S4.4. Tweets expressed positive feelings about the future openings of mass cultural events and following health measures to assist research. Tweets also expressed positive feelings about the artists and music at the events and about Liverpool itself. However, other Tweets expressed concerns about neglecting small venues, complications in the booking processes, the behaviour of fellow event participants, the government, noise, ethical concerns, lack of scientific rigour, and annoyance about having to take tests.

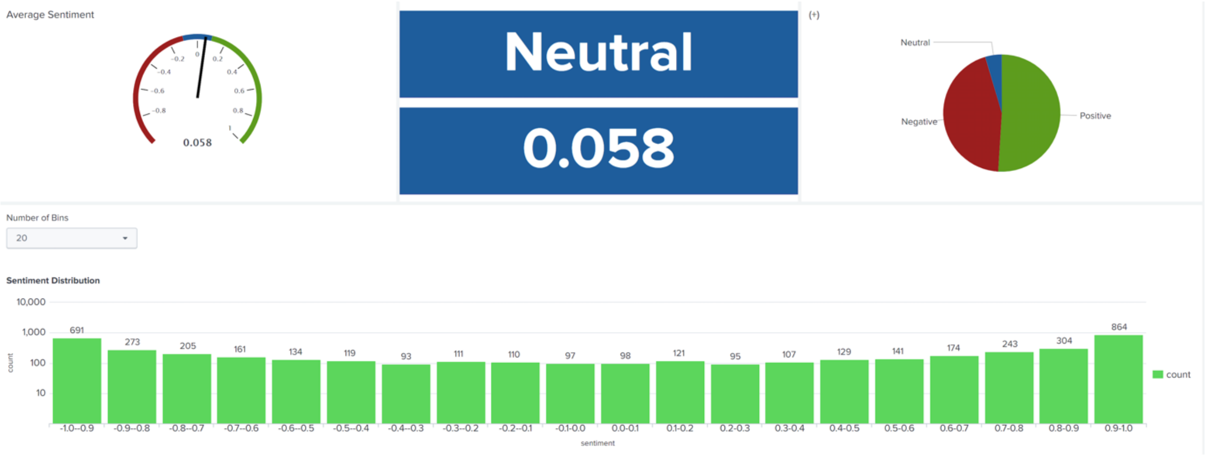

Figure S4.3 Sentiment Scores for Public Comments about the night club event

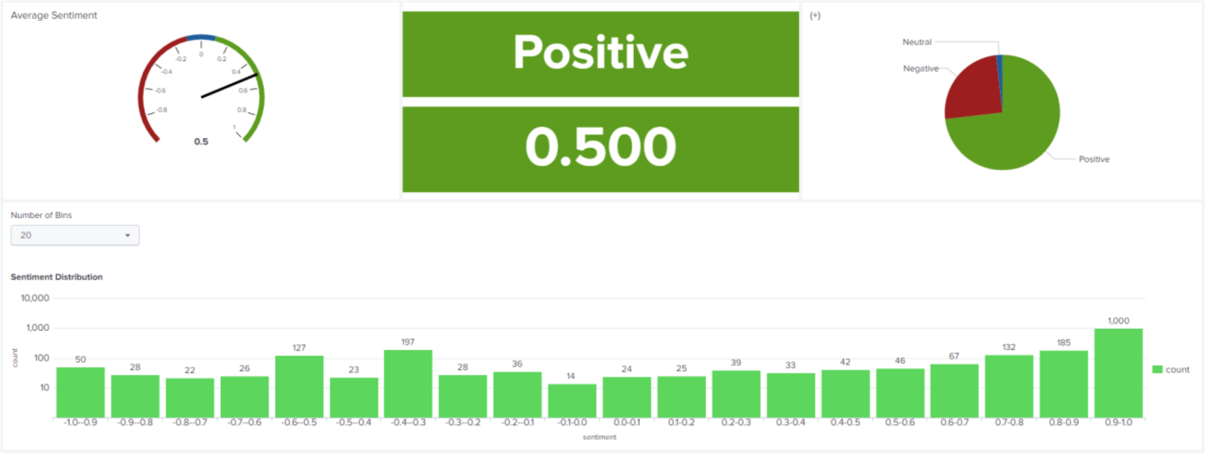

Figure S4.4 Sentiment Scores for Public Tweets

Discussions about falsifying LFTs were found in a small number of Tweets (38), with a negative sentiment about the practice, indicating that people do not approve of this behaviour. In addition, public comments (1320) on six videos about ‘testing the test kit’ to get negative results on TikTok were condemned (regarding wastage of kits). By contrast, 2500 comments on 50 TikTok videos that showed how to fake positive results ranged from amusement on the one hand to condemnation on the other, again condemning waste of scarce kits needed for legitimate public health purposes.
