# Supplementary material for: COVID-19 risk mitigation in reopening mass cultural events: population-based observational study for the UK Events Research Programme in Liverpool City Region
Source: J R Soc Med. 2023 Jun 23;117(1):11–23. doi: 10.1177/01410768231182389 (PMC10858718; doi:10.1177/01410768231182389)
Supplement: sj-pdf-5-jrs-10.1177_01410768231182389 - Supplemental material for COVID-19 risk mitigation in reopening mass cultural events: population-based observational study for the UK Events Research Programme in Liverpool City Region [file sj-pdf-5-jrs-10.1177_01410768231182389.pdf]

Appendix 5: Communications

Emails sent to attendees of the Good Business Festival

Pre-show email 1

Important information about what happens next...

View email in browser

The Good Business Festival presents 'Change Business for Good'

THE GOOD BUSINESS FESTIVAL.

Wednesday 28 April 2021, 14:30
ACC Liverpool

Dear Customer,

Thank you for booking your ticket for The Good Business Festival presents 'Change Business for Good'. You should have received your e-ticket by now in a separate email.

Now all you need to do is plan to take your Lateral Flow Test between 07:00 and 21:00 on Tuesday 27 April, the day before the event. Your test must be taken at one of the below designated community test centres. Click here to check how busy or quiet each test site is.

- Exhibition Centre Liverpool, King's Dock, L3 4FP
- Liverpool One, Unit 73, L1 8BU
- Liverpool FC Anfield Stadium, Anfield Road, L4 0TH

• Liverpool Tennis Centre, Wavertree Sports Park, L15 4LE

Please see below information to help you plan your test:

A quick reminder of the testing process:

- Your Lateral Flow Test must be taken between 07:00 and 21:00 on Tuesday 27 April, the day before the event
- Please ensure that the information you give when taking your Lateral Flow Test matches the booking data you provided on our website.
- Once you have taken your test and received a negative result, you do not need to do anything further, we will contact you a few days before the event to let you know what to expect on arrival.
- You do not need to show your negative test result upon entry; your e-ticket is all you will need on arrival. You may be asked to show photo ID that matches the name on your ticket.
- If you return a positive, void or inconclusive Lateral Flow Test result, or fail to take a test within the pre-event testing window, we will contact you to cancel your tickets.
- Although the only compulsory test needed to attend the event is the Lateral Flow Test the day before the event, you will also be asked to take a free at-home PCR test on the day of the event and also five days after the event. This is non-mandatory but is critical for the event research data required by the scientists.
- You'll be given your two at-home PCR tests when you attend your Lateral Flow Test at one of the designated community test centres.

For more information regarding the government's Events Research Project (ERP) take a look at our detailed FAQs.

Still have questions? No problem. Please email erp@ticketquarter.co.uk and our team will be happy to help.

Kind Regards,

The Ticket Quarter Team.

Pre-show email 2

We can't wait to see you...

View email in browser

The Good Business Festival presents 'Change Business for Good'

THE GOOD BUSINESS FESTIVAL.

Wednesday 28 April 2021, 14:30-19:30
ACC Liverpool

Dear Customer,

We are looking forward to welcoming you to The Good Business Festival presents 'Change Business for Good' and 'Comics Released' on Wednesday 28 April 2021. Please note that doors open at 13:00 with refreshments provided before the event starts at 14:30.

There's only a few days to go before you will be able to enjoy your first non-socially distanced event in over a year and we can’t wait to see you!

In typical TGBF style, the event will bring together big thinkers from across politics, culture and business with a stellar line up, including Tanya Beckett, Sharmadean Reid MBE and Jeremy Schwartz. Make sure you stay for an evening of stand up comedy after the business event because it's 2021 and we all need a laugh.

We'll be sending you full event details, including timings and arrival instructions, on the morning of the event - so please keep your eyes peeled.

There's still time to register, so spread the word and bring along a friend or colleague!

Now all you need to do is plan to take your Lateral Flow Test between 07:00 and 21:00 tomorrow, Tuesday 27 April, the day before the event.

Your test should be taken at one of the below designated community test centres. Click here to check how busy or quiet each test site is.

- Exhibition Centre Liverpool, King's Dock, L3 4FP
- Liverpool One, Unit 73, L1 8BU
- Liverpool FC Anfield Stadium, Anfield Road, L4 0TH
- Liverpool Tennis Centre, Wavertree Sports Park, L15 4LE

Technically you can go to any test centre in the Liverpool City Region to take this test, however the test centres above are getting extra support specifically for this event and as such will have more staffing and longer opening hours.

Your test is matched to your ticket through the NHS testing system, so it is crucial that you fill in the personal details required - name, date of birth and postcode - exactly as you filled them out when you bought your ticket. We've included the info you provided us below, so please make sure you use these same details at your test:
Anna Moscardini
L3 4FP
26/10/1990

A quick reminder of the testing process:

- Your Lateral Flow Test must be taken between 07:00 and 21:00 tomorrow, Tuesday 27 April, the day before the event.
- Your test is matched to your ticket through the NHS testing system, so it is crucial that you fill in the personal details required - name, date of birth and address - exactly as you filled them out when you bought your ticket. If you want to know more about the use of data then check out the privacy statement here.
- To give the best chance of the match happening, adding your NHS number will help, which can be found here
- Once you have taken your test and received a negative result, you do not need to do anything further, we will contact you on the morning of the event to let you know what to expect on arrival.

- You do not need to show your negative test result upon entry; your e-ticket is all you will need on arrival. You may also be asked to show photo ID that matches the name on your ticket.
- If you return a positive, void or inconclusive Lateral Flow Test result, or fail to take a test within the pre-event testing window, we will contact you to cancel your tickets.
- You will also be asked to take a free at-home PCR test on the day of the event and also five days after the event. This is non-mandatory but is critical for the event research data required by the scientists, and the results will help re-open the events sector.
- You'll be given your two at-home PCR tests when you attend your Lateral Flow Test at one of the designated community test centres.
- If you have had a "confirmed positive PCR test" in the last 30 days prior to the event, you should not request or use the PCR tests supplied for the Events Research Programme. You must still attend an Asymptomatic Testing Site to take a Lateral Flow Device test prior to attending the event.
- If you have travelled abroad in the last 10 days or been in contact with someone who has or are experiencing any symptoms of illness, then please click here for additional guidance on event entry.

For more information regarding the government's Events Research Project (ERP) take a look at our detailed FAQs.

Still have questions? No problem. Please email erp@ticketquarter.co.uk and our team will be happy to help.

Kind Regards,

The Ticket Quarter Team.

Pre-show email 3

We can't wait to see you...

View email in browser

The Good Business Festival presents 'Change Business for Good'

THE GOOD BUSINESS FESTIVAL.

Wednesday 28 April 2021, Doors open at 13:00
ACC Liverpool

Dear Customer,

You're all set to attend The Good Business Festival presents 'Change Business for Good' on Wednesday 28 April 2021 at ACC Liverpool. Please show your e-ticket upon arrival at the venue.

Please do take time to read all the information below before you arrive at the venue.

What time should I arrive?
Doors open: 13:00, with refreshments available upon arrival
Event starts: 14:30

What to expect when you arrive...
Information about how to find the venue, and parking options, can be found here.

Upon arrival, please make your way to ACC Liverpool's Galleria entrance, which can be found on the map at the bottom of this email. You will be asked to show your e-ticket, and may also be asked to show photo ID that matches the name on your ticket. Please remember that until you enter the venue, you must follow existing Covid-19 government guidelines.

Once inside the event there will be no requirement to wear a face covering or practice social distancing. The purpose of this is to test specific settings to collect evidence and best practice. If you would like to wear a face covering anyway, please do so.

Event Overview
13:00 - Doors open and refreshments served
14:30-17:30 - The Good Business Festival Presents Change Business For Good
Including contributions from:

- Nigel Huddleston MP – Tourism and Sport Minister
- Paul Mason – Award Winning British Journalist, Broadcaster & Author
- Sharmadean Reid MBE - entrepreneur and founder of WAH Nails and The Stack World
- Simone Roche MBE – Founder, Northern Power Women
- Jeremy Schwartz – Former CEO of The Body Shop
- Felicia Odamtten - Founder & Director of Black Economists Network
- Tony Reeves - Chief Executive of Liverpool City Council
- Mark Stevenson – 'Futurist' and author

17:30-18:30 - Drinks reception with catering by Homebaked
18:30 - The Good Business Festival presents 'Comics Released' with Comedy Stop
Live at the Apollo style comedy show with;

- Mick Ferry
- Rachel Fairburn
- Kiri Pritchard-Mclean

• **19:30** - Event ends

A little reminder…
We also ask that you please take a free at-home PCR test on the day of the event. This is a non-mandatory but important part of the event research data required by the scientists. You should have received your two PCR testing kits when you attended your Lateral Flow Test - if not, you can order them here.

It's so important to the Events Research Programme that you take your second free at home event PCR test 5 days after the event on Monday 3 May. We thank you for the important role you are playing in helping to get live events and venues back open this summer!

You must not attend this event if you or anyone you live with are experiencing any symptoms of Covid-19, including fever, cough, loss or change in sense of smell or taste, you feel generally unwell or have been abroad in the last 10 days.

For more information regarding the government's Events Research Project (ERP) take a look at our detailed FAQs.

Still have questions? No problem. Please email erp@ticketquarter.co.uk and our team will be happy to help.

Kind Regards,

The Ticket Quarter Team.

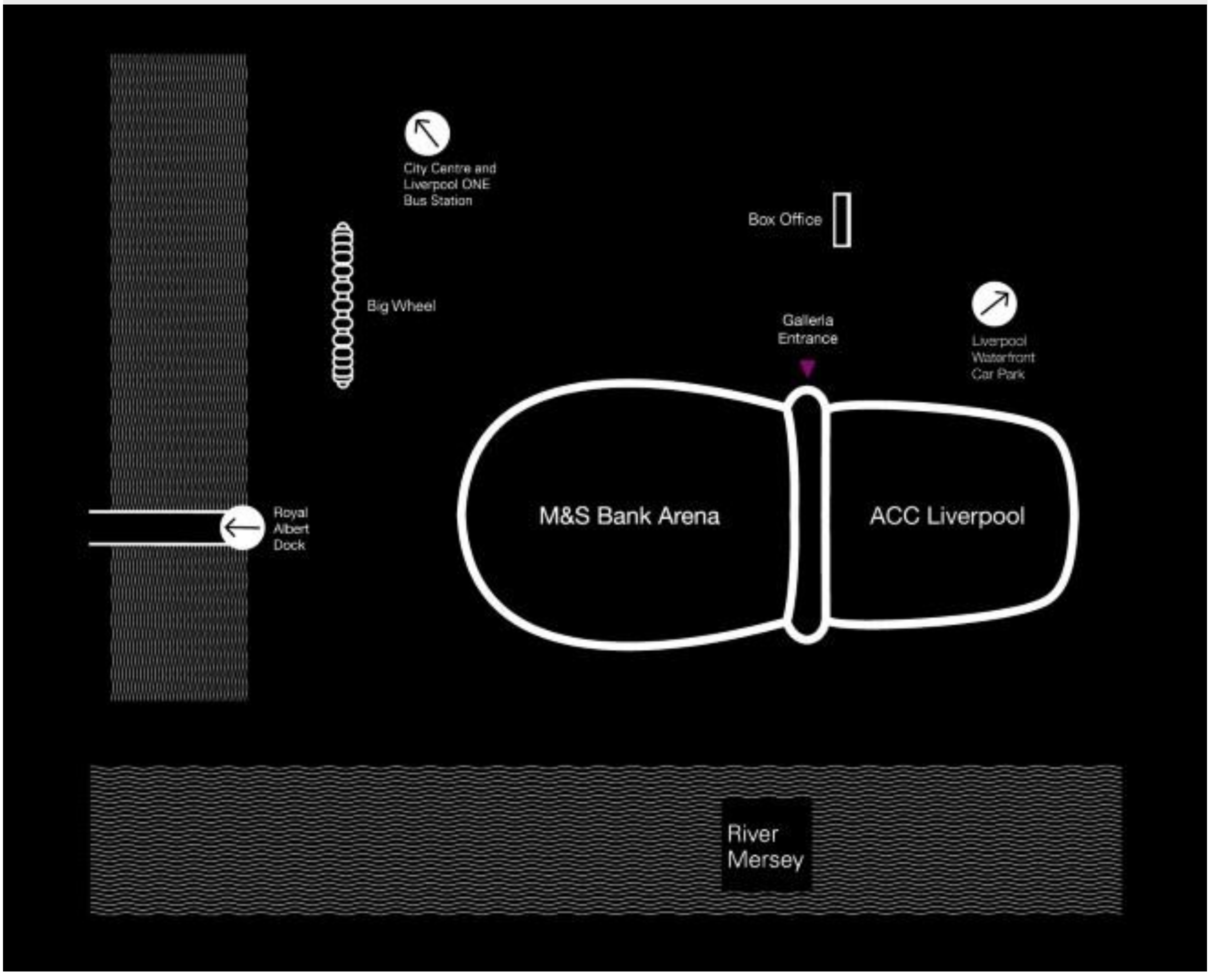

Post show email

Make sure you take your post-event PCR test...

View email in browser

The Good Business Festival presents 'Change Business for Good'

THE GOOD BUSINESS FESTIVAL.

Dear Customer,

Thank you for registering to attend The Good Business Festival presents 'Change Business for Good' yesterday. If you were able to make it to the event we hope you enjoyed it and had a great time socialising with other people!

If you attended

Please make sure that you take extra care over the next few days - stick to Government guidance on masks and social distancing and try to avoid mixing outside of your household group. Please note that if you experience any symptoms of COVID-19 (a high temperature, a new, continuous cough or a loss or change to your sense of smell or taste) you will be required to isolate in line with the latest government guidelines.

Next steps in the Event Research Programme

If you attended the event we have two things to ask you to do:

1. Below is a unique link to a post event questionnaire which is being managed by University of Liverpool as part of the scientific evaluation of the pilot events. The survey will take less than 5 minutes to complete and is an opportunity to provide feedback on the event and share details about your experience – both of which are essential to better inform future activity.

2. Don't forget to take your at home PCR Covid-19 test on **Tuesday 4 May.** You should have received your free PCR testing kit when you attended your Lateral Flow Test - if not, you can order one here. Please note that you need to post your completed test kit on the same day you take the test and no later than 1 hour before last collection. You can find a list of priority post boxes nearest to you and their collection times here.

The results of these tests are critical to the research study, so please do make time to take the questionnaire now and the test in a few days. We will make sure to text you a reminder.

TAKE THE QUESTIONNAIRE

Once again, thanks for the important role you are playing in helping to get live events and venues back open this summer!

Have questions? No problem. Please email erp@ticketquarter.co.uk and our team will be happy to help.

Kind Regards,

The Ticket Quarter Team.

Emails sent to attendees of the Circus nightclub

Pre-show email 1

Important information about what happens next...

View email in browser

Circus Presents The First Dance

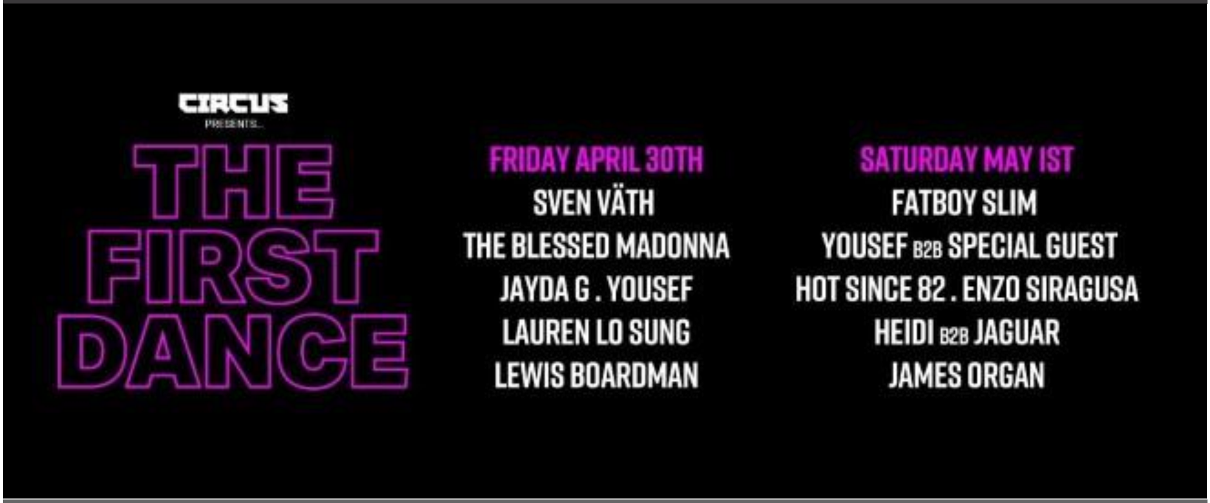

Friday 30 April 2021, 14:00-23:00
Bramley Moore Dock Warehouse

Dear Customer,

Thank you for booking your ticket for Circus Presents The First Dance, on Friday 30 April 2021. You should have received your e-ticket by now in a separate email.

Now all you need to do is plan to take your Lateral Flow Test between 07:00 and 21:00 on Thursday 29 April, which is the day before the event. Your test must be taken at one of the below designated community test centres. Click here to check how busy or quiet each test site is.

- Exhibition Centre Liverpool, King's Dock, L3 4FP
- Liverpool One, Unit 73, L1 8BU
- Liverpool FC Anfield Stadium, Anfield Road, L4 0TH
- Liverpool Tennis Centre, Wavertree Sports Park, L15 4LE

Please see below information to help you plan your test:

A quick reminder of the testing process:

- Your Lateral Flow Test must be taken between 07:00 and 21:00 on Thursday 29 April, which is the day before the event.
- Please ensure that the information you give when taking your Lateral Flow Test matches the booking data you provided on our website.
- Once you have taken your test and received a negative result, you do not need to do anything further, we will contact you a few days before the event to let you know what to expect on arrival.
- You do not need to show your negative test result upon entry; your e-ticket is all you will need on arrival. You may also be asked to present photo ID that matches the name on your ticket.
- Anyone who registers to attend and then returns a positive, inconclusive or void Lateral Flow Test result for Covid-19 will be unable to attend. In this case your ticket price will be refunded automatically. If your test is positive then you will be required to quarantine in line with the latest Government guidelines.
- Anyone who fails to take their Lateral Flow test within the specified window will also be unable to attend but will not receive a refund.
- Although the only compulsory test needed to attend the event is the Lateral Flow Test the day before the event, you will also be asked to take a free at-home PCR test on the day of the event and also five days after the event. This is non-mandatory but is critical for the event research data required by the scientists.
- You'll be given your two at-home PCR tests when you attend your Lateral Flow Test at one of the designated community test centres.

For more information take a look at our detailed FAQs

Make sure you've purchased your Cashless Vouchers for the event **here.** By buying in advance you'll receive additional free credit on all vouchers of £30 or more.

Still have questions? No problem. Please email **erp@ticketquarter.co.uk** and our team will be happy to help.

Kind Regards,

The Ticket Quarter Team.

Pre-show email 2

It's vital that you take your pre-event test..

View email in browser

Circus Presents The First Dance

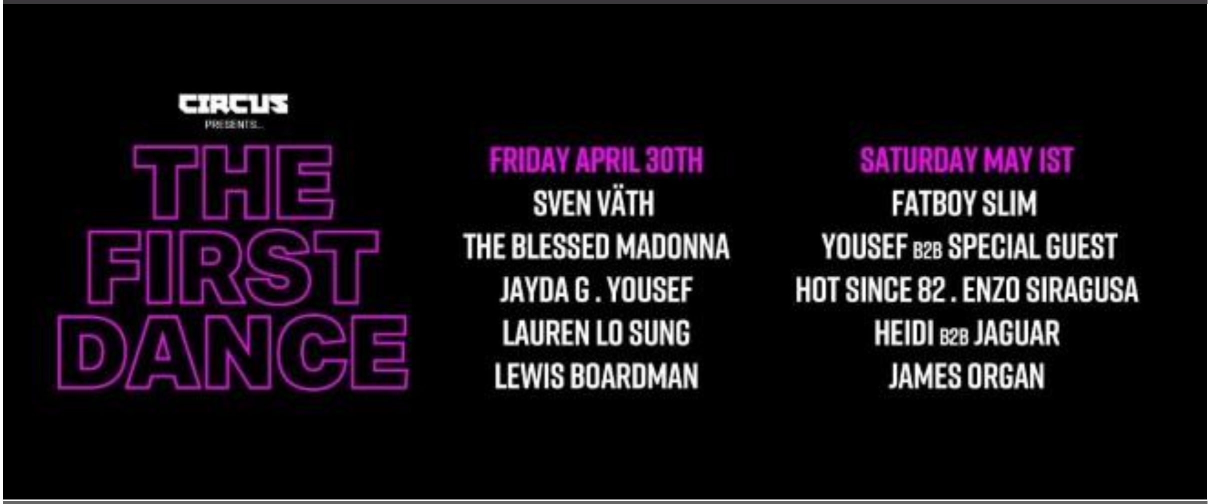

Friday 30 April 2021, 14:00-23:00
Bramley Moore Dock

Dear Customer,

We are looking forward to welcoming you to Circus Presents The First Dance on Friday 30 April 2021. Please note that doors are at 2pm and last entry is 6pm. The event will finish at 11pm.

There are only a few days to go before you will be able to enjoy your first non-socially distanced event in over a year and we can’t wait to see you!

Please remember that you need to plan to take your Covid-19 test between 07:00 and 22:00 on Thursday 29 April, which is the day before the event .

Your test should be taken at one of the following designated sites. Click here to check how busy or quiet each test site is.

- Exhibition Centre Liverpool, King's Dock, L3 4FP
- Liverpool One, Unit 73, L1 8BU
- Liverpool FC Anfield Stadium, Anfield Road, L4 0TH
- Liverpool Tennis Centre, Wavertree Sports Park, L15 4LE

Technically you can go to any test centre in the Liverpool City Region to take this test, however the test centres above are getting extra support specifically for this event and as such will have more staffing and longer opening hours.

Your test is matched to your ticket through the NHS testing system, so it is crucial that you fill in the personal details required - name, date of birth and postcode - exactly as you filled them out when you bought your ticket. We've included the info you provided us below, so please make sure you use these same details at your test:
Anna
Moscardini
L3 4FP
26/10/1990

A quick reminder of the testing process:

- Your Lateral Flow Test must be taken between 07:00 and 22:00 on Thursday 29 April, the day before the event.
- Your test is matched to your ticket through the NHS testing system, so it is crucial that you fill in the personal details required - name, date of birth and address - exactly as you filled them out when you bought your ticket. If you want to know more about the use of data then check out the privacy statement here.
- To give the best chance of the match happening, adding your NHS number will help, which can be found here
- Once you have taken your test and received a negative result, you do not need to do anything further, we will contact you on the morning of the event to let you know what to expect on arrival.
- You do not need to show your negative test result upon entry; your e-ticket is all you will need on arrival. You may also be asked to show photo ID that matches the name on your ticket.
- If you return a positive, void or inconclusive Lateral Flow Test result, or fail to take a test within the pre-event testing window, we will contact you to cancel your tickets.
- You will also be asked to take a free at-home PCR test on the day of the event and also five days after the event. This is non-mandatory but is critical for the event research data required by the scientists.
- You'll be given your two at-home PCR tests when you attend your Lateral Flow Test at one of the designated community test centres.
- If you have had a "confirmed positive PCR test" in the last 30 days prior to the event, you should not request or use the PCR tests supplied for the Events Research Programme. You must still attend an Asymptomatic Testing Site to take a Lateral Flow Device test prior to attending the event.

- If you have travelled abroad in the last 10 days or been in contact with someone who has or are experiencing any symptoms of illness, then please click here for additional guidance on event entry.

Please take the time to read the full terms and conditions of entry for this event, including information about the dress code, food and drink, and prohibited items.

For more information regarding the government's Events Research Project (ERP) take a look at our detailed FAQs.

Make sure you've purchased your Cashless Vouchers for the event **here.** By buying in advance you'll receive additional free credit on all vouchers of £30 or more.

Still have questions? No problem. Please email erp@ticketquarter.co.uk and our team will be happy to help.

Kind Regards,

The Ticket Quarter Team.

Pre-show email 3

You'll need your test result, e-ticket and ID...

View email in browser

Circus Presents The First Dance

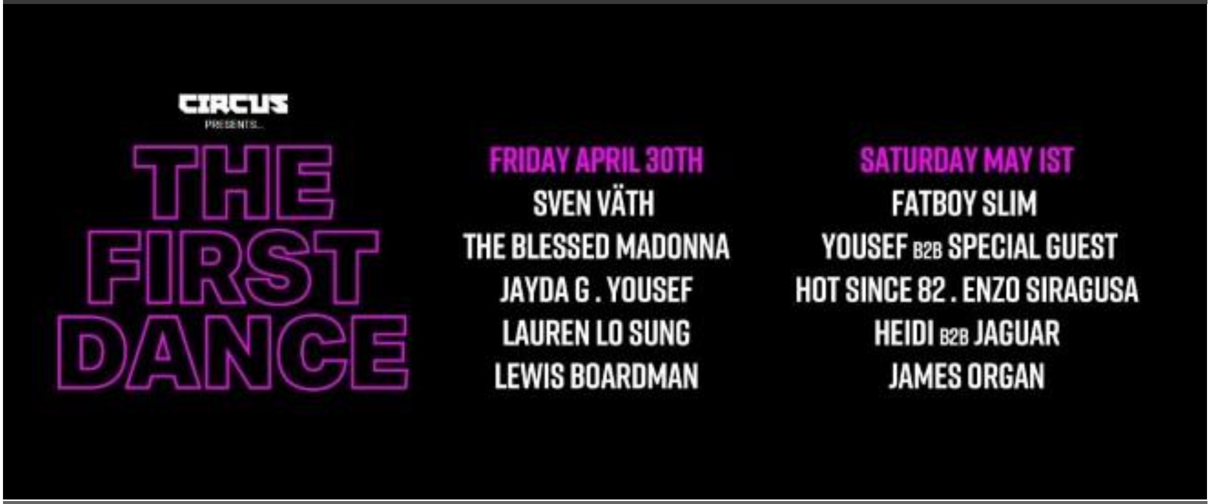

Friday 30 April, 14:00-23:00
Bramley Moore Dock

Dear Customer,

You're all set to attend Circus Presents The First Dance **today** at Bramley Moore Dock Warehouse.
When you arrive, you'll be asked to show:

- **A negative Lateral Flow Test - the original email or text result you received from the NHS after visiting a test centre yesterday.**
- **Your e-ticket for the event**
- **Photo ID that proves your Liverpool City Region address and that you are over 18. If you are a student your University ID Card can be used to prove that you live in the Liverpool City Region, but you will also need a passport or driver's license as proof of age.**

Please note that this email is not an e-ticket. Your e-ticket is attached to your original confirmation email, sent by tickets@ticketquarter.co.uk with the subject line 'Confirmation of Order Number XXXX'. This will have been sent to you immediately after purchasing your tickets. Please have your e-ticket downloaded to your e-wallet or ready on your phone for entry, and have the brightness turned up on your phone.

Please remember that until you enter the venue, you must follow existing Covid-19 government guidelines. This means that you will need to socially distance and wear a face covering before you enter the venue and once you leave. Anyone not socially distancing will be instructed to do so by the stewarding team, non-compliance could result in you being refused entry to the event.

When should I arrive?
Doors are at 2pm, and we recommend that you arrive as early as possible to avoid any queues. Last entry is at 6pm, and the event will finish at 11pm.

What to expect when you arrive...
Once inside the event there will be no requirement to wear a face covering or practice social distancing. The purpose of this is to test specific settings to collect evidence and best practice. If you would like to wear a face covering anyway, please do so.

Please take the time to read the entry requirements for the show, this includes key information about catering, dress code, prohibited items and more.

A little reminder…
We also ask that you please take a free at-home PCR test on the day of the event. This is a non-mandatory but important part of the event research data required by the scientists. You should have received your two free PCR testing kits when you attended your Lateral Flow Test - if not, you can order them here.

It's so important to the Events Research Programme that you take your second free post event PCR test 5 days after the event on Monday 3 May. We thank you for the important role you are playing in helping to get live events and venues back open this summer!

You must not attend this event if you or anyone you live with are experiencing any symptoms of Covid-19, including fever, cough, loss or change in sense of smell or taste, you feel generally unwell or have been abroad in the last 10 days.

Still have questions? No problem. Please email erp@ticketquarter.co.uk and our team will be happy to help.

Kind Regards,

The Ticket Quarter Team.

Post-show email 1

If you attended make sure you take a post-event PCR test...

View email in browser

Circus Presents The First Dance

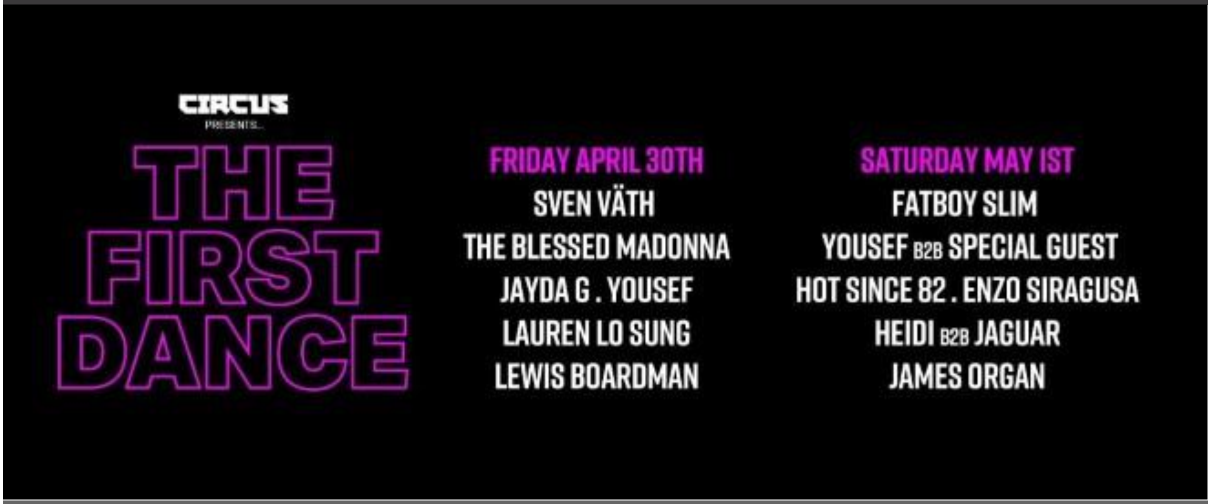

Friday 30 April 2021
Bramley Moore Dock Warehouse

Dear Customer,

Thank you for buying tickets and supporting Circus Presents The First Dance. If you were able to attend the show we hope you enjoyed your night and had a great time socialising with other people!

If you attended

Please make sure that you take extra care over the next few days - stick to Government guidance on masks and social distancing and try to avoid mixing outside of your household group. Please note that if you experience any symptoms of COVID-19 (a high temperature, a new, continuous cough or a loss or change to your sense of smell or taste) you will be required to isolate in line with the latest government guidelines.

Next steps in the Event Research Programme

If you attended the event we have two things to ask you to do:

1. Below is a unique link to a post event questionnaire which is being managed by University of Liverpool as part of the scientific evaluation of the pilot events. The survey will take less

than 5 minutes to complete and is an opportunity to provide feedback on the event and share details about your experience – both of which are essential to better inform future activity.

2. Don't forget to take your at home PCR Covid-19 test on **Wednesday 5 May.** You should have received your free PCR testing kit when you attended your Lateral Flow Test - if not, you can order one here. Please note that you need to post your completed test kit on the same day you take the test and no later than 1 hour before last collection. You can find a list of priority post boxes nearest to you and their collection times here.

The results of these tests are critical to the research study, so please do make time to take the questionnaire now and the test in a few days. We will make sure to text you a reminder.

TAKE THE QUESTIONNAIRE

Once again, thanks for the important role you are playing in helping to get live events and venues back open this summer!

Have questions? No problem. Please email erp@ticketquarter.co.uk and our team will be happy to help.

Kind Regards,

The Ticket Quarter Team.

Post-show email 2

Take your PCR test today...

View email in browser

Circus Presents The First Dance

Friday 30 April 2021
Bramley Moore Dock Warehouse

Dear Customer,

Thank you for supporting Circus Presents The First Dance. If you were able to attend the show we hope you enjoyed your night and had a great time socialising with other people!

Don't forget to take your at home PCR Covid-19 test today, Wednesday 5 May.

You should have received your free PCR testing kit when you attended your Lateral Flow Test. Please note that you need to post your completed test kit on the same day you take the test and no later than 1 hour before last collection. You can find a list of priority post boxes nearest to you and their collection times here.

The results of these tests are critical to the research study, so please do make time to take it today.

Once again, thanks for the important role you are playing in helping to get live events and venues back open this summer!

Kind Regards,

The Ticket Quarter Team.

Emails sent to attendees of the Sefton Park pilot

Pre-show email 1

Important information about what happens next...

View email in browser

Sefton Park Pilot

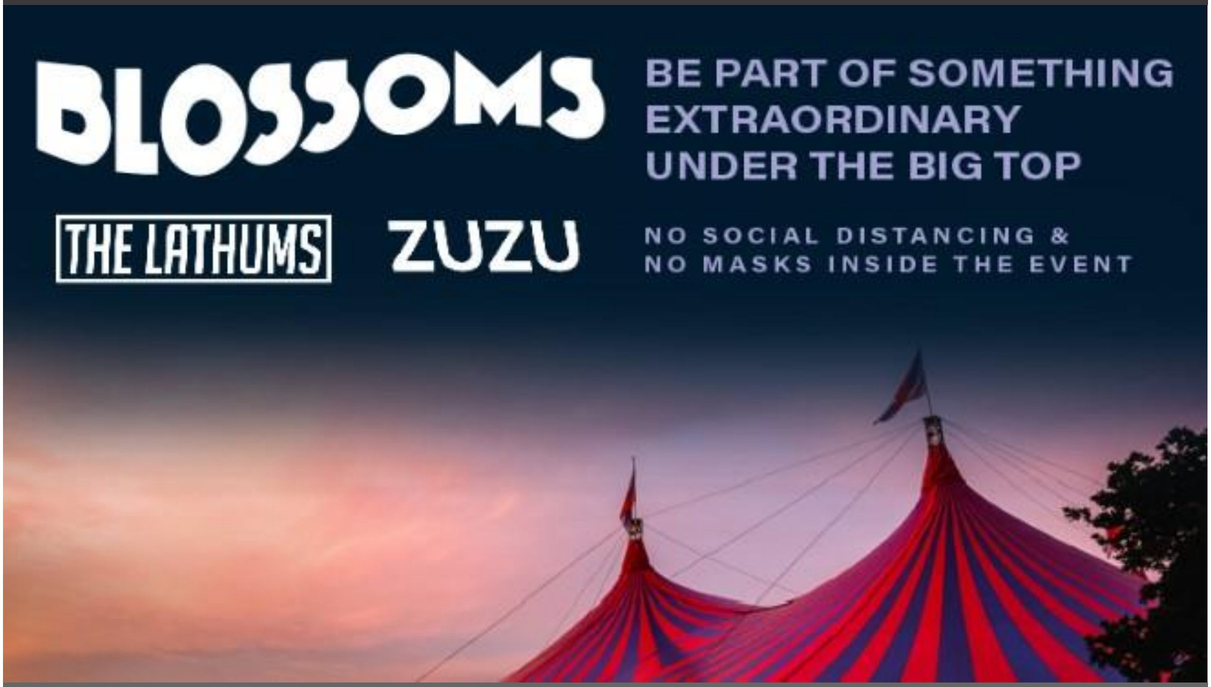

Sunday 2 May 2021, Gates at 16:30
Sefton Park

Dear Customer,

Thank you for booking your ticket for the Sefton Park Pilot. You should have received your e-ticket by now in a separate email.

Now all you need to do is plan to take your Lateral Flow Test between 07:00 and 21:00 on Saturday 1st May, which is the day before the event. Your test must be taken at one of the below designated community test centres. Click here to check how busy or quiet each test site is.

- Exhibition Centre Liverpool, King's Dock, L3 4FP
- Liverpool One, Unit 73, L1 8BU
- Liverpool FC Anfield Stadium, Anfield Road, L4 0TH
- Liverpool Tennis Centre, Wavertree Sports Park, L15 4LE

Please see below information to help you plan your test:

A quick reminder of the testing process:

- Your Lateral Flow Test must be taken between 07:00 and 21:00 on Saturday 1 May, which is the day before the event.
- Please ensure that the information you give when taking your Lateral Flow Test matches the booking data you provided on our website.
- Once you have taken your test and received a negative result, you do not need to do anything further, we will contact you a few days before the event to let you know what to expect on arrival.
- You do not need to show your negative test result upon entry; your e-ticket is all you will need on arrival. You may also be asked to present photo ID that matches the name on your ticket.
- Anyone who registers to attend and then returns a positive, inconclusive or void Lateral Flow Test result for Covid-19 will be unable to attend. In this case your ticket price will be refunded automatically. If your test is positive then you will be required to quarantine in line with the latest Government guidelines.
- Anyone who fails to take their Lateral Flow test within the specified window will also be unable to attend but will not receive a refund.
- Although the only compulsory test needed to attend the event is the Lateral Flow Test the day before the event, you will also be asked to take a free at-home PCR test on the day of the event and also five days after the event. This is non-mandatory but is critical for the event research data required by the scientists.
- You'll be given your two at-home PCR tests when you attend your Lateral Flow Test at one of the designated community test centres.

For your safety we ask that you do not bring a bag any larger than an A4 piece of paper.

For more information take a look at our detailed FAQs and the terms and conditions of entry for this event.

Still have questions? No problem. Please email erp@ticketquarter.co.uk and our team will be happy to help.

Kind Regards,

The Ticket Quarter Team.

Pre-show email 2

It's vital that you take your Lateral Flow Test...

View email in browser

Sefton Park Pilot

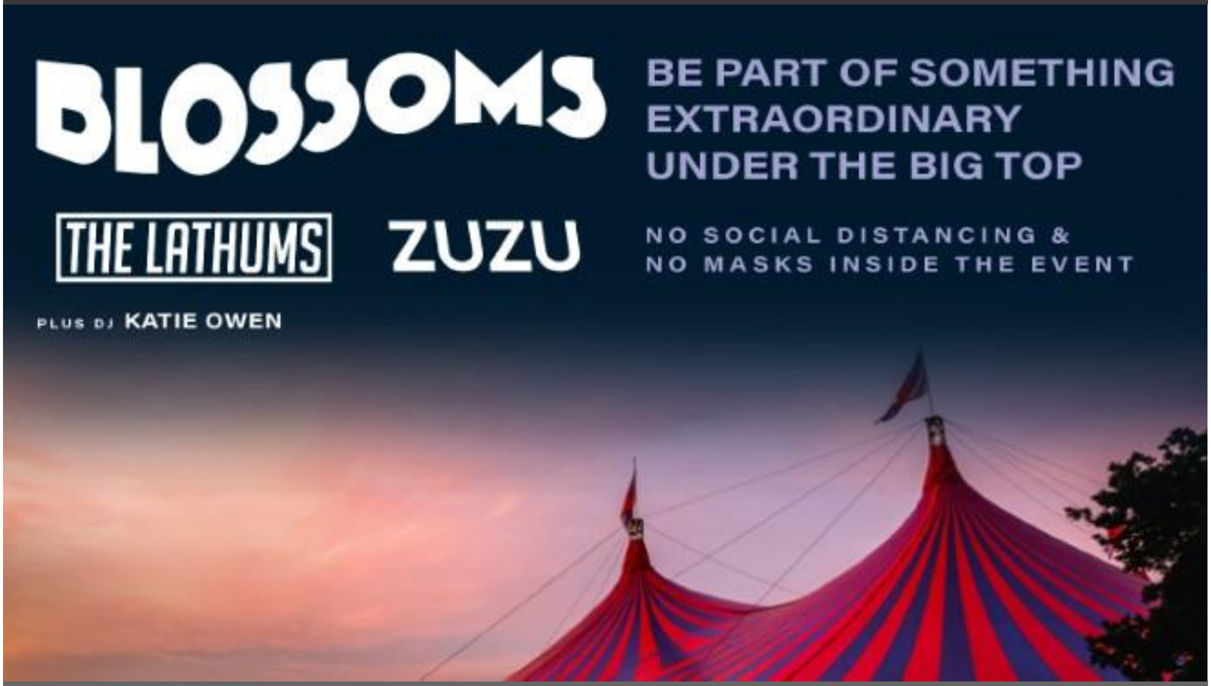

Sunday 2 May 2021, Gates at 16:30
Sefton Park

Dear Customer,

We are looking forward to welcoming you to the Sefton Park Pilot on Sunday 2 May 2021.

There are only a few days to go before you will be able to enjoy your first non-socially distanced event in over a year and we can't wait to see you!

Please remember that you need to plan to take your Covid-19 test between 07:00 and 22:00 on Saturday 1 May, which is the day before the event. Your test must be taken at one of the following designated sites. Click here to check how busy or quiet each test site is.

- Exhibition Centre Liverpool, King's Dock, L3 4FP
- Liverpool One, Unit 73, L1 8BU
- Liverpool FC Anfield Stadium, Anfield Road, L4 0TH
- Liverpool Tennis Centre, Wavertree Sports Park, L15 4LE

Technically you can go to any test centre in the Liverpool City Region to take this test, however the test centres above are getting extra support specifically for this event and as such will have more staffing and longer opening hours.

Your test is matched to your ticket through the NHS testing system, so it is crucial that you fill in the personal details required - name, date of birth and postcode - exactly as you filled them out when you bought your ticket. As a reminder, these are the details you provided when you booked your ticket:
Anna Moscardini
L3 4FP
26/10/1990

A quick reminder of the testing process:

- Your Lateral Flow Test must be taken between 07:00 and 22:00 on Saturday 1 May, which is the day before the event
- Your test is matched to your ticket through the NHS testing system, so it is crucial that you fill in the personal details required - name, date of birth and address - exactly as you filled them out when you bought your ticket. If you want to know more about the use of data then check out the privacy statement here.
- To give the best chance of the match happening, adding your NHS number will help, which can be found here
- Once you have taken your test and received a negative result, you do not need to do anything further, we will contact you a few days before the event to let you know what to expect on arrival.
- If you return a positive, void or inconclusive Lateral Flow Test result, or fail to take a test within the pre-event testing window, we will contact you to cancel your tickets.
- You will also be asked to take a free at-home PCR test on the day of the event and also five days after the event. This is non-mandatory but is critical for the event research data required by the scientists.
- You'll be given your two at-home PCR tests when you attend your Lateral Flow Test at one of the designated community test centres.
- If you have had a "confirmed positive PCR test" in the last 30 days prior to the event, you should not request or use the PCR tests supplied for the Events Research Programme. You must still attend an Asymptomatic Testing Site to take a Lateral Flow Device test prior to attending the event.
- If you have travelled abroad in the last 10 days or been in contact with someone who has or are experiencing any symptoms of illness, then please click here for additional guidance on event entry.

When Travelling to the event…
Remember that government guidance should be followed until you are inside the event. This means wearing a face covering whilst on public transport unless you are exempt, sanitizing your hands regularly and keeping your distance where possible. If you can walk or cycle to the event, even better! The same goes for your journey home – you will need to abide by current government rules.

What to expect when you arrive...
Once inside the event there will be no requirement to wear a face covering or practice social distancing. The purpose of this is to test specific settings to collect evidence and best practice. If you would like to wear a face covering anyway, please do so.

For more information regarding the government's Events Research Project (ERP) take a look at our detailed FAQs and the terms and conditions of entry for this event.

Still have questions? No problem. Please email erp@ticketquarter.co.uk and our team will be happy to help.

Kind Regards,

The Ticket Quarter Team.

Pre-show email 3

You'll need your test result, e-ticket and ID...

View email in browser

Sefton Park Pilot

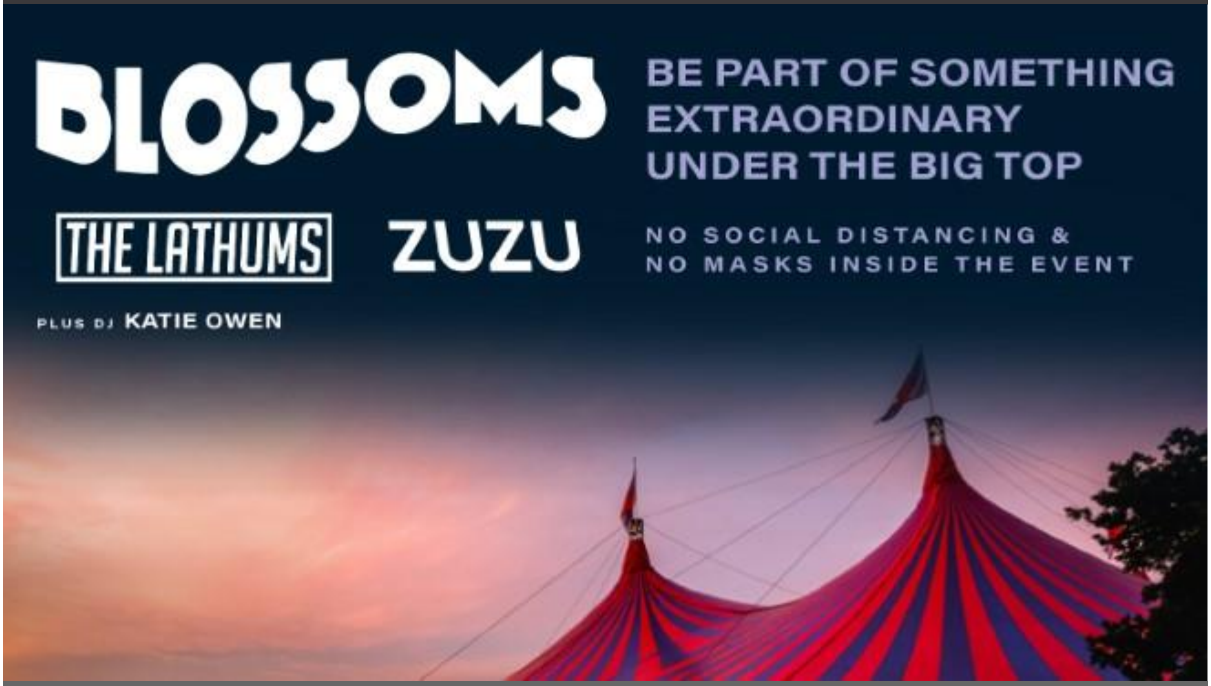

Sunday 2 May 2021, Gates at 16:30
Sefton Park

Dear Customer,

You're all set to attend the Sefton Park Pilot **tomorrow, Sunday 2 May.**

When you arrive, you'll be asked to show:

- **A negative Lateral Flow Test - the original email or text result you will receive from the NHS after visiting a test centre today.**
- **Your e-ticket for the event**
- **Photo ID in the form of a passport or drivers licence that proves you are over 18 and that you live in Liverpool City Region. If your photo ID does not display a Liverpool City Region address, then you must also bring a phone bill, utility bill or other documentation with this on. If you are a student, you may use your Student ID Card to prove your address.**

Please note that this email is not an e-ticket. Your e-ticket is attached to your original confirmation email, sent by tickets@ticketquarter.co.uk with the subject line 'Confirmation of

Order Number XXXX'. This will have been sent to you immediately after purchasing your tickets. Please have your e-ticket downloaded to your e-wallet or ready on your phone for entry, and have the brightness turned up on your phone. You can also print off your e-ticket and be scanned in that way.

When should I arrive?
Gates open at 16:30 and last entry is at 20:30. The event will finish at 22:00.

When Travelling to the event…
Please remember that until you enter the venue, you must follow existing Covid-19 government guidelines. This means that you will need to socially distance and wear a face covering before you enter the venue and once you leave. Anyone not socially distancing will be instructed to do so by the stewarding team, non-compliance could result in you being refused entry to the event.

Please see the map at the bottom of the email for details of the entrance point for this event.

What to expect when you arrive...
Once inside the event there will be no requirement to wear a face covering or practise social distancing. The purpose of this is to test specific settings to collect evidence and best practice. If you would like to wear a face covering anyway, please do so.

We ask that you do not bring bags larger than A4 in size, cans, alcohol of any kind, glass, audio recorders, umbrellas, camping chairs and inflatable loungers. You can bring a sealed and untampered soft drink of 500ml or less, and you can bring your own empty reusable bottle to fill from the water points in the event for free. Please note that the event is cashless, so bars and food traders will only accept cards and contactless payments.

Please take the time to read the full terms and conditions of entry for the event.

A little reminder…
We also ask that you please take a free at-home PCR test on the day of the event. This is a non-mandatory but important part of the event research data required by the scientists. You should have received your two free PCR testing kits when you attended your Lateral Flow Test - if not, you can order them here.

It's so important to the Events Research Programme that you take your second free post event PCR test 5 days after the event on Friday 7 May. We thank you for the important role you are playing in helping to get live events and venues back open this summer!

You must not attend this event if you or anyone you live with are experiencing any symptoms of Covid-19, including fever, cough, loss or change in sense of smell or taste, you feel generally unwell or have been abroad in the last 10 days.

Still have questions? No problem. Please email erp@ticketquarter.co.uk and our team will be happy to help.

Kind Regards,
The Ticket Quarter Team.

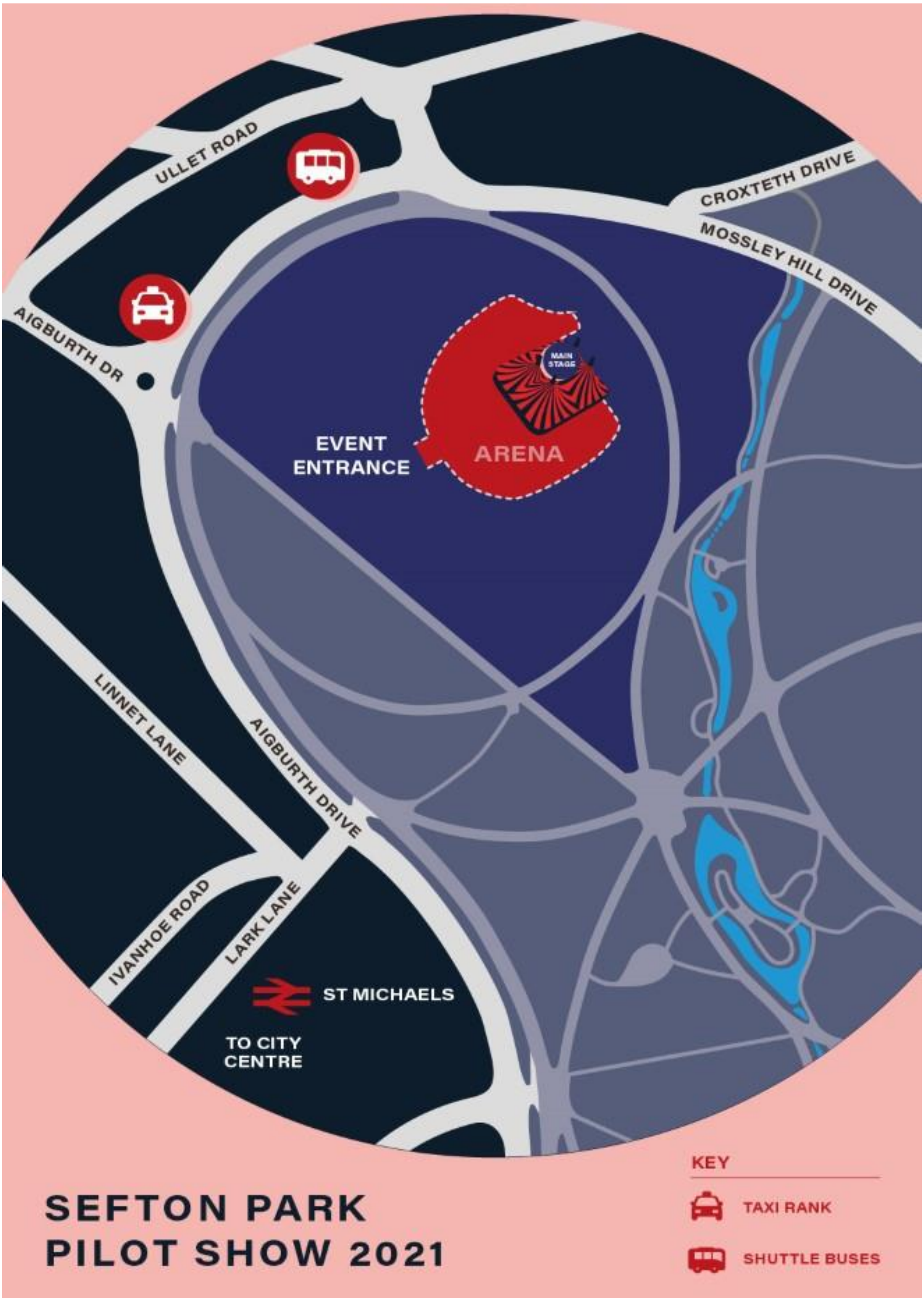
ULLET ROAD
CROXTETH DRIVE
MOSSLEY HILL DRIVE
AIGBURTH DR
MAIN STAGE
EVENT ENTRANCE
ARENA
LINNET LANE
AIGBURTH DRIVE
IVANHOE ROAD
LARK LANE
ST MICHAELS
TO CITY CENTRE
SEFTON PARK
PILOT SHOW 2021
KEY
TAXI RANK
SHUTTLE BUSES

Post-show email 1

If you attended make sure you take a post-event PCR test...

View email in browser

Sefton Park Pilot

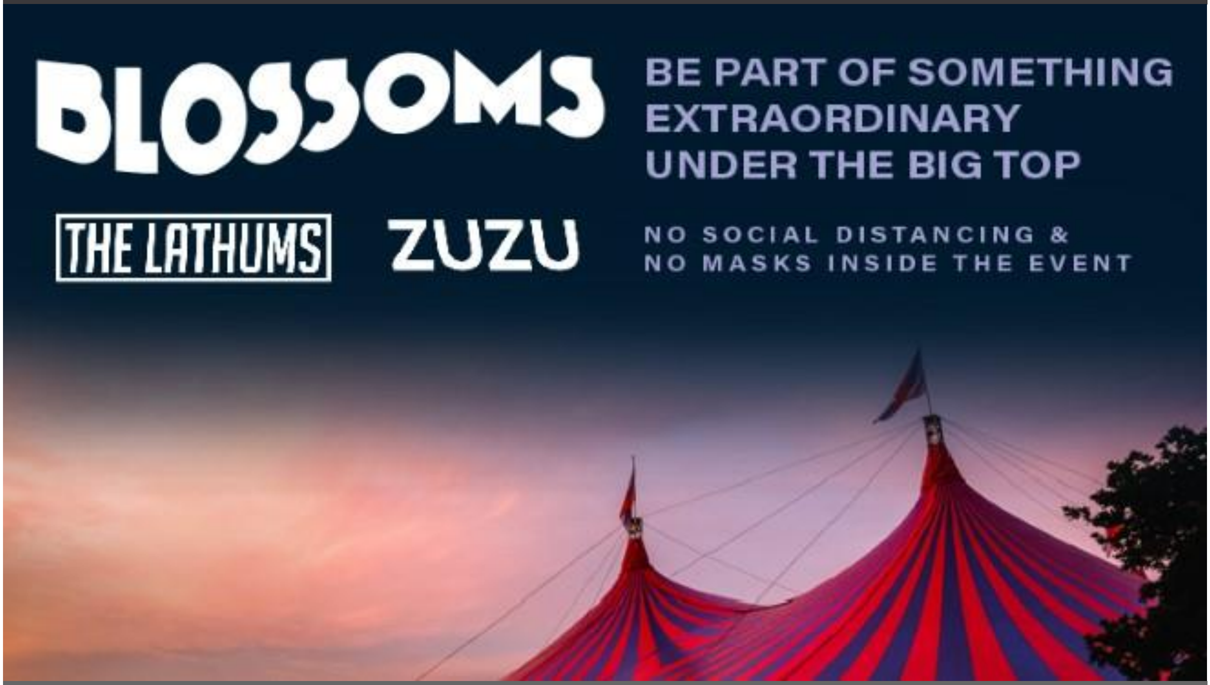

Sunday 2 May 2021
Sefton Park

Dear Customer,

Thank you for buying tickets and supporting the Sefton Park Pilot. If you were able to attend the show we hope you enjoyed your night and had a great time socialising with other people!

If you attended

Please make sure that you take extra care over the next few days - stick to Government guidance on masks and social distancing and try to avoid mixing outside of your household group. Please note that if you experience any symptoms of COVID-19 (a high temperature, a new, continuous cough or a loss or change to your sense of smell or taste) you will be required to isolate in line with the latest government guidelines.

Next steps in the Event Research Programme

If you attended the event we have two things to ask you to do:

1. Below is a unique link to a post event questionnaire which is being managed by University of Liverpool as part of the scientific evaluation of the pilot events. The survey will take less than 5 minutes to complete and is an opportunity to provide feedback on the event and share details about your experience – both of which are essential to better inform future activity.

2. Don't forget to take your at home PCR Covid-19 test on **Friday 7 May.** You should have received your free PCR testing kit when you attended your Lateral Flow Test - if not, you can order one here. Please note that you need to post your completed test kit on the same day you take the test and no later than 1 hour before last collection. You can find a list of priority post boxes nearest to you and their collection times here.

The results of these tests are critical to the research study, so please do make time to take the questionnaire now and the test in a few days. We will make sure to text you a reminder.

TAKE THE QUESTIONNAIRE

Once again, thanks for the important role you are playing in helping to get live events and venues back open this summer!

Have questions? No problem. Please email erp@ticketquarter.co.uk and our team will be happy to help.

Kind Regards,

The Ticket Quarter Team.

Post-show email 2

All you have to do is take your PCR test...

View email in browser

Want to win festival and gig tickets?

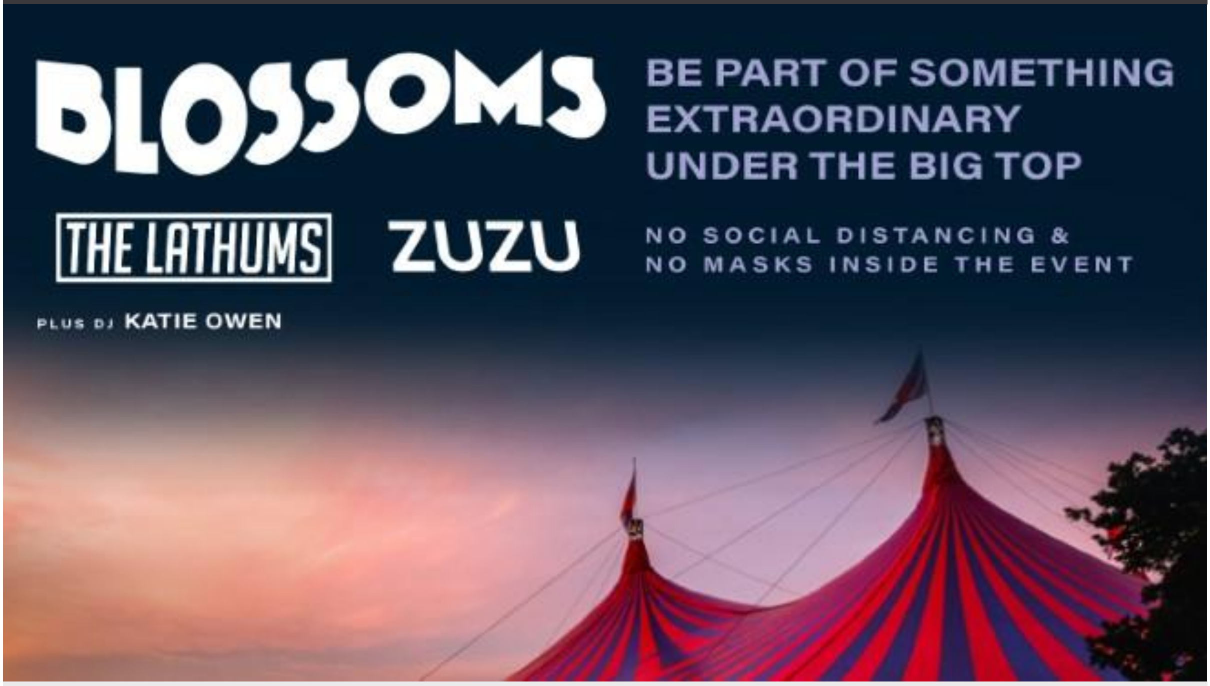

Dear Customer,

Thank you for buying tickets and supporting the Sefton Park Pilot. If you were able to attend the show we hope you enjoyed your night and had a great time socialising with other people!

Who wants to win festival and gig tickets?

Complete and return your post-event PCR test by the end of the day **tomorrow, Friday 7 May,** to be in with a chance of winning one of 450 pairs of tickets to the best shows across the UK, from Leeds Festival and Boardmasters, to any gig at the O2 Academy Liverpool!

To enter, simply take your PCR test, return by post using the instructions on the testing kit, and fill in the below application form.

The first 450 applicants will be entered into the raffle so get in there quickly to be in with the chance to attend some of the best events of the summer - many of which are already sold out! For a full list of tickets on offer, T&Cs and entry details, please click here.

Good Luck!

ENTER THE DRAW

You should have received your free PCR testing kit when you attended your Lateral Flow Test - if not, you can order one here.

Please note that you need to post your completed test kit on the same day you take the test and no later than 1 hour before last collection. You can find a list of priority post boxes nearest to you and their collection times here. If you have had a "confirmed positive PCR test" in the last 30 days prior to the event, you should not request or use the PCR tests supplied for the Events Research Programme.

How did you find the event?

As part of the Events Research Programme, the Department for Digital, Culture Media and Sport and the University of Edinburgh are conducting research on attendee experience of crowd events and the Covid-19 guidance.

In order to help us improve events in the future, we'd be grateful if you could tell them about your experience by taking their short survey below.

They are also running short online interviews to hear about experiences of events during COVID-19 - ideally within 1 week of your visit. If you want to take part, please email Anne Templeton at a.templeton@ed.ac.uk

TAKE THE QUESTIONNAIRE

Once again, thanks for the important role you are playing in helping to get live events and venues back open this summer!

Kind Regards,

The Ticket Quarter Team.

Post-show email 3

...and win festival and gig tickets!

View email in browser

Want to win festival and gig tickets?

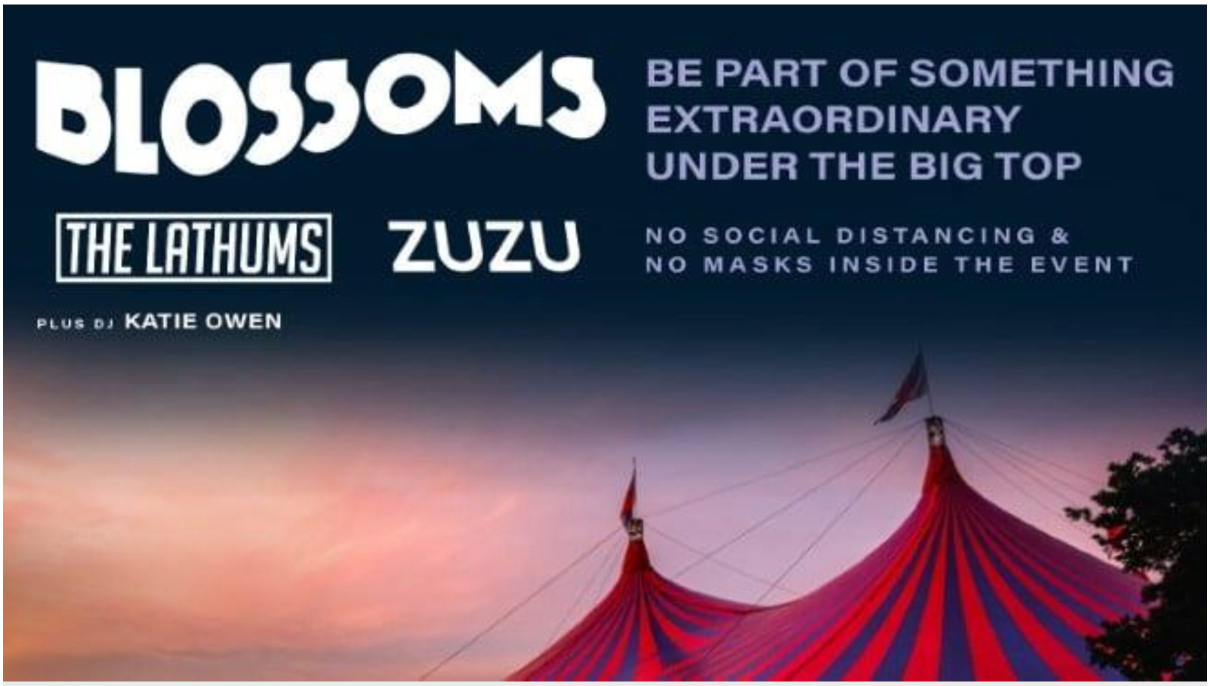

Dear Customer,

Thank you for buying tickets and supporting the Sefton Park Pilot. If you were able to attend the show we hope you enjoyed your night and had a great time socialising with other people!

Who wants to win festival and gig tickets?

Complete and return your post-event PCR test by the end of the day **today, Friday 7 May,** to be in with a chance of winning one of 450 pairs of tickets to the best shows across the UK, from Leeds Festival and Boardmasters, to any gig at the O2 Academy Liverpool!

To enter, simply take your PCR test, return by post using the instructions on the testing kit, and fill in the below application form.

The first 450 applicants will be entered into the raffle so get in there quickly to be in with the chance to attend some of the best events of the summer - many of which are already sold out! For a full list of tickets on offer, T&Cs and entry details, please click here.

Good Luck!

ENTER THE DRAW

You should have received your free PCR testing kit when you attended your Lateral Flow Test.

Please note that you need to post your completed test kit on the same day you take the test and no later than 1 hour before last collection. You can find a list of priority post boxes nearest to you and their collection times here. If you have had a "confirmed positive PCR test" in the last 30 days prior to the event, you should not request or use the PCR tests supplied for the Events Research Programme.

We need your feedback

Below is a unique link to a post event questionnaire which is being managed by University of Liverpool as part of the scientific evaluation of the pilot events. The survey will take less than 5 minutes to complete and is an opportunity to provide feedback on the event and share details about your experience – both of which are essential to better inform future activity.

TAKE THE QUESTIONNAIRE

Once again, thanks for the important role you are playing in helping to get live events and venues back open this summer!

Kind Regards,

The Ticket Quarter Team.
