# Supplementary material for: COVID-19 risk mitigation in reopening mass cultural events: population-based observational study for the UK Events Research Programme in Liverpool City Region
Source: J R Soc Med. 2023 Jun 23;117(1):11–23. doi: 10.1177/01410768231182389 (PMC10858718; doi:10.1177/01410768231182389)
Supplement: sj-pdf-6-jrs-10.1177_01410768231182389 - Supplemental material for COVID-19 risk mitigation in reopening mass cultural events: population-based observational study for the UK Events Research Programme in Liverpool City Region [file sj-pdf-6-jrs-10.1177_01410768231182389.pdf]

Appendix 6: Questionnaires from Events Research Programme Liverpool

Pre-event screening questionnaire

Pre-event screening questionnaire variables by ticket purchase (whether or not a pre-event questionnaire could be linked to a ticket) from Good Business Festival

	Variable	Summary		
		Purchased a ticket	Not purchased a ticket	Overall
1	Number of participants (with completed pre-event screening questionnaire)	373	207	580
2 (Q2)	Are you concerned about attending the event?			
	Missing, n (%)	138 (37.0%)	25 (12.1%)	163 (28.1%)
	Responders, n (%)	235 (63.0%)	182 (87.9%)	417 (71.9%)
	Not at all concerned, n (%)	*136 (57.9%)*	*105 (57.7%)*	*241 (57.8%)*
	Slightly concerned, n (%)	*72 (30.6%)*	*57 (31.3%)*	*129 (30.9%)*
	Moderately concerned, n (%)	*25 (10.6%)*	*17 (9.3%)*	*42 (10.1%)*
	Very concerned, n (%)	*2 (0.9%)*	*3 (1.6%)*	*5 (1.2%)*
3 (Q3)	Are there any factors that might contribute to some of your concern about attending the event?			
	Missing, n (%)	275(73.7%)	133 (64.3%)	408 (70.3%)
	Responders, n (%)	98 (26.3%)	74 (35.7%)	172 (29.7%)
	Possibly catching Covid-19, n (%)	*70 (71.4%)*	*45 (60.8%)*	*115(66.9%)*
	Impact of having to self-isolate if I test positive for Covid-19, n (%)	*38 (38.8%)*	*26 (35.1%)*	*64 (37.2%)*
	Social anxiety, n (%)	*33 (33.7%)*	*23 (31.1%)*	*56 (32.6%)*
	Others thinking I'm reckless, n (%)	*20 (20.4%)*	*22 (29.7%)*	*42 (24.4%)*
	Other, n (%)	*29 (29.6%)*	*25 (33.8%)*	*54 (31.4%)*
4 (Q4)	How likely do you think you are to catch coronavirus at the event?			
	Missing, n (%)	136 (36.5%)	25 (12.1%)	161 (27.8%)
	Responders, n (%)	237 (63.5%)	182 (87.9%)	419 (72.2%)
	Very unlikely, n (%)	*94 (39.7%)*	*59 (32.4%)*	*153 (36.5%)*
	Fairly unlikely, n (%)	*109 (46.0%)*	*88 (48.4%)*	*197 (47.0%)*
	Neither unlikely nor likely, n (%)	*31 (13.1%)*	*32 (17.6%)*	*63 (15.0%)*
	Fairly likely, n (%)	*3 (1.2%)*	*2 (1.1%)*	*5 (1.2%)*
	Very likely, n (%)	*0 (0.0%)*	*1 (0.5%)*	*1 (0.2%)*
5 (Q7)	How concerned are you about potentially infecting others after attending the event?			
	Missing, n (%)	137 (36.7%)	27 (13.0%)	164 (28.3%)
	Responders, n (%)	236 (63.3%)	180 (87.0%)	416 (71.7%)
	Not at all concerned, n (%)	*102 (43.2%)*	*91 (50.6%)*	*193 (46.4%)*
	Slightly concerned, n (%)	*75 (31.8%)*	*51 (28.3%)*	*126 (30.3%)*
	Neither concerned nor unconcerned, n (%)	*24 (10.2%)*	*12 (6.7%)*	*36 (8.7%)*
	Moderately concerned, n (%)	*30 (12.7%)*	*21 (11.6%)*	*51(12.2%)*
	Very concerned, n (%)	*5 (2.1%)*	*5 (2.8%)*	*10 (2.4%)*
6 (Q8)	If 'not at all concerned' on last question, having stated that you are not concerned, share reasons why			
	Missing, n (%)	272(72.9%)	116(56.0%)	388 (66.9%)

	Responders, n (%)	101 (27.1%)	91 (44.0%)	192 (33.1%)
	Because I've previously had Covid-19, n (%)	*19 (18.8%)*	*17 (18.7%)*	*36(18.8%)*
	Because I've been tested, n (%)	*59 (58.4%)*	*48 (52.7%)*	*107 (55.7%)*
	Because I've been vaccinated, n (%)	*58 (57.4%)*	*57 (62.6%)*	*115 (59.9%)*
	Because I've followed guidance on reducing the spread of Covid-19, n (%)	*72 (71.3%)*	*62 (68.1%)*	*134 (69.8%)*
	Other, n (%)	*5 (5.0%)*	*6 (6.6%)*	*11 (5.7%)*
7 (Q9)	How important do you think it is to resume these kinds of public events as soon as possible?			
	Missing, n (%)	136 (36.5%)	28 (13.5%)	164 (28.3%)
	Responders, n (%)	237 (63.5%)	179 (86.5%)	416 (71.7%)
	Not at all important, n (%)	*0 (0.0%)*	*4 (2.2%)*	*4 (1.0%)*
	Slightly important, n (%)	*4 (1.7%)*	*9 (5.0%)*	*13 (3.1%)*
	Neither important nor unimportant, n (%)	*3 (1.3%)*	*5 (2.8%)*	*8 (1.9%)*
	Moderately important, n (%)	*63 (26.6%)*	*44 (24.9%)*	*107 (25.7%)*
	Very important, n (%)	*167 (70.4%)*	*117 (65.4%)*	*284 (68.3%)*
8 (Q10)	In the past seven days, how often did you wash your hands with soap and water straight away after returning home from a public place?			
	Missing, n (%)	136 (36.5%)	27 (13.0%)	163 (28.1%)
	Responders, n (%)	237 (63.5%)	180 (87.0%)	417 (71.9%)
	Always, n (%)	*161 (67.9%)*	*136 (75.6%)*	*297 (71.2%)*
	Often, n (%)	*62 (26.2%)*	*39 (21.7%)*	*101 (24.2%)*
	Sometimes, n (%)	*9 (3.8%)*	*2 (1.1%)*	*11 (2.6%)*
	Not very often, n (%)	*4 (1.7%)*	*2 (1.1%)*	*6 (1.4%)*
	Never, n (%)	*1 (0.4%)*	*1 (0.5%)*	*2 (0.5%)*
9 (Q11)	In the past seven days, have you used a face covering when outside your home to help slow the spread of the coronavirus (COVID-19)?			
	Missing, n (%)	137 (36.7%)	29 (14.0%)	166 (28.6%)
	Responders, n (%)	236 (63.3%)	178 (86.0%)	414 (71.4%)
	Yes, n (%)	*225 (95.3%)*	*170 (95.5%)*	*395 (95.4%)*
	No, n (%)	*10 (4.2%)*	*5 (2.8%)*	*15 (3.6%)*
	Not applicable, n (%)	*1 (0.4%)*	*3 (1.7%)*	*4 (1.0%)*
10 (Q12)	While you were inside a public space (e.g., shop, public transport) in the last seven days, how often did you wear a protective face covering to help slow the spread of the coronavirus (COVID-19)?			
	Missing, n (%)	137 (36.7%)	28 (13.5%)	165 (28.4%)
	Responders, n (%)	236 (63.3%)	179 (86.5%)	415 (71.6%)
	Always, n (%)	*225 (95.4%)*	*167 (93.3%)*	*392 (94.5%)*
	Often, n (%)	*9 (3.8%)*	*6 (3.4%)*	*15 (3.6%)*
	Sometimes, n (%)	*0 (0.0%)*	*0 (0.0%)*	*0 (0.0%)*
	Not very often, n (%)	*1 (0.4%)*	*1 (0.5%)*	*2 (0.5%)*
	Never, n (%)	*1 (0.4%)*	*5 (2.8%)*	*6 (1.4%)*
11 (Q13)	On average, how often do you follow the guidance on social distance when outside of support/childcare bubbles, maintaining 1-2 metres between yourself and other people?	137 (36.7%)	27 (13.0%)	164 (28.3%)
	Missing, n (%)	236 (63.3%)	180 (87.0%)	416 (71.7%)
	Responders, n (%)	*137 (58.1%)*	*119 (66.1%)*	*256 (61.5%)*

	Always, n (%)	*85 (36.0%)*	*44 (24.4%)*	*129 (31.0%)*
	Often, n (%)	*11 (4.7%)*	*13 (7.2%)*	*24 (5.8%)*
	Sometimes, n (%)	*3 (1.3%)*	*2 (1.1%)*	*5 (1.2%)*
	Not very often, n (%)	*0 (0.0%)*	*2 (1.1%)*	*2 (0.5%)*
	Never, n (%)			
12 (Q14)	In the past seven days, have you had any visitors inside your home from outside your support/childcare bubbles, including trades people, carers or medical staff?	138 (37.0%)	31 (15.0%)	169 (29.1%)
	Missing, n (%)	235 (63.0%)	176 (85.0%)	411 (70.9%)
	Responders, n (%)	*45 (19.1%)*	*35 (19.9%)*	*80 (19.5%)*
	Yes, n (%)	*190 (80.9%)*	*141 (80.1%)*	*331 (80.5%)*
	No, n (%)			
13 (Q15)	When you have had a visitor inside your home, which of the following actions did you take to reduce the spread of the coronavirus (COVID-19)?			
	Missing, n (%)	190 (50.9%)	50 (24.2%)	240 (41.4%)
	Responders, n (%)	183 (49.1%)	157 (75.8%)	340 (58.6%)
	Worn a face mask, n (%)	*67 (36.6%)*	*59 (37.6%)*	*126 (37.1%)*
	Asked the visitor to wear a mask, n (%)	*71 (38.8%)*	*52 (33.1%)*	*123 (36.2%)*
	Opened windows or doors, n (%)	*100 (54.6%)*	*90 (57.3%)*	*190 (55.9%)*
	Cleaned touch points, n (%)	*80 (43.7%)*	*65 (41.4%)*	*145 (42.6%)*
	Maintained social distancing, n (%)	*122 (66.7%)*	*108 (68.8%)*	*230 (67.6%)*
	Washed hands regularly, n (%)	*108 (59.0%)*	*87 (55.4%)*	*195 (57.4%)*
	Other, n (%)	*22 (12.0%)*	*8 (5.1%)*	*30 (8.8%)*
	None of the above, n (%)	*27 (14.8%)*	*28 (17.8%)*	*55 (16.2%)*
14 (Q16)	As part of your condition for attendance to this event, you will be required to complete a test. In case that this test results comes back negative, which statement below best describes what it means to you?			
	Missing, n (%)	136 (36.5%)	30 (14.5%)	166 (28.6%)
	Responders, n (%)	237 (63.5%)	177 (85.5%)	414 (71.4%)
	I am definitely not infectious, n (%)	*51 (21.5%)*	*55 (31.1%)*	*106 (25.6%)*
	I am probably not infectious, n (%)	*181 (76.4%)*	*115 (65.0%)*	*296 (71.5%)*
	I am probably infectious, n (%)	*0 (0.0%)*	*3 (1.7%)*	*3 (0.7%)*
	I am definitely infectious, n (%)	*2 (0.8%)*	*1 (0.5%)*	*3 (0.7%)*
	Don't know, n (%)	*3 (1.3%)*	*3 (1.7%)*	*6 (1.4%)*
15 (Q18)	What gender do you most identify with?			
	Missing, n (%)	138 (37.0%)	30 (14.5%)	168 (29.0%)
	Responders, n (%)	235 (63.0%)	177 (85.5%)	412 (71.0%)
	Man, n (%)	*113 (48.1%)*	*77 (43.5%)*	*190 (46.1%)*
	Woman, n (%)	*121 (51.5%)*	*97 (54.8%)*	*218 (52.9%)*
	Non-binary, n (%)	*0 (0.0%)*	*0 (0.0%)*	*0 (0.0%)*
	Other, n (%)	*0 (0.0%)*	*1 (0.6%)*	*1 (0.2%)*
	Prefer not to say, n (%)	*1 (0.4%)*	*2(1.1%)*	*3 (0.7%)*
16 (Q19)	Ethnicity			
	Missing, n (%)	136 (36.5%)	30 (14.5%)	166 (28.6%)
	Responders, n (%)	237 (63.5%)	177 (85.5%)	414 (71.4%)
	Asian/Asian-British- Indian, Pakistani, Bangladeshi, other, n (%)	*3 (1.3%)*	*4 (2.3%)*	*7 (1.7%)*
	Black/Black British- Caribbean. African, other, n (%)	*6 (2.5%)*	*4 (2.3%)*	*10 (2.4%)*

	Mixed race- White and Black/Black British, n (%)	*3 (1.3%)*	*5 (2.8%)*	*8 (1.9%)*
	Mixed race- other, n (%)	*2 (0.8%)*	*5 (2.8%)*	*7 (1.7%)*
	White- British, Irish, other, n (%)	*218 (92.0%)*	*151 (85.3%)*	*369 (89.1%)*
	Chinese/Chinese British, n (%)	*1 (0.4%)*	*0 (0.0%)*	*1 (0.2%)*
	Middle Eastern/Middle Eastern British- Arab, Turkish, other, n (%)	*0 (0.0%)*	*0 (0.0%)*	*0 (0.0%)*
	Other ethnic group, n (%)	*1 (0.4%)*	*1 (0.6%)*	*2 (0.5%)*
	Prefer not to say, n (%)	*3 (1.3%)*	*7 (4.0%)*	*10 (2.4%)*
17 (Q21)	Have you previously been diagnosed with Covid?			
	Missing, n (%)	137 (36.7%)	30 (14.5%)	167 (28.8%)
	Responders, n (%)	236 (63.3%)	177 (85.5%)	413 (71.2%)
	Yes, I've had a positive test (antibody or swab), n (%)	*31 (13.1%)*	*19 (10.7%)*	*50 (12.1%)*
	Yes, most likely but I've not had a test to confirm, n (%)	*25 (10.6%)*	*17 (9.6%)*	*42 (10.2%)*
	No, don't think I've had Covid-19, n (%)	*159 (67.4%)*	*125 (70.6%)*	*284 (68.8%)*
	Don't know, n (%)	*21 (8.9%)*	*16 (9.1%)*	*37 (8.9%)*
18 (Q23)	Have you received your vaccination?			
	Missing, n (%)	136 (36.5%)	29 (14.0%)	165 (28.4%)
	Responders, n (%)	237 (63.5%)	178 (86.0%)	415 (71.6%)
	Yes, I have received my first vaccine dose, n (%)	*125 (52.7%)*	*100 (56.2%)*	*225 (54.2%)*
	Yes, I have received both vaccine doses, n (%)	*27 (11.4%)*	*23 (12.9%)*	*50 (12.0%)*
	No, n (%)	*85 (35.9%)*	*51 (28.7%)*	*136 (32.8%)*
	Don't know, n (%)	*0 (0.0%)*	*4 (2.2%)*	*4 (1.0%)*

Pre-event screening questionnaire variables by attendance (whether the linked ticket was scanned or not) from Good Business Festival

	Variable	Summary		
		Tickets scanned	Tickets not scanned	Overall
1	Number of participants (with completed pre-event screening questionnaire)	149	224	373
2 (Q2)	Are you concerned about attending the event?			
	Missing, n (%)	1 (0.7%)	137 (61.2%)	138 (37.0%)
	Responders, n (%)	148 (99.3%)	87 (38.8%)	235 (63.0%)
	Not at all concerned, n (%)	*80 (54.0%)*	*56 (64.4%)*	*136 (57.9%)*
	Slightly concerned, n (%)	*51 (34.5%)*	*21 (24.1%)*	*72 (30.6%)*
	Moderately concerned, n (%)	*16 (10.8%)*	*9 (10.3%)*	*25 (10.6%)*
	Very concerned, n (%)	*1 (0.7%)*	*1 (1.2%)*	*2 (0.9%)*
3 (Q3)	Are there any factors that might contribute to some of your concern about attending the event?			
	Missing, n (%)	82 (55.0%)	193 (86.2%)	275 (73.7%)
	Responders, n (%)	67 (45.0%)	31 (13.8%)	98 (26.3%)
	Possibly catching Covid-19, n (%)	*47 (70.1%)*	*23 (74.2%)*	*70(71.4%)*
	Impact of having to self-isolate if I test positive for Covid-19, n (%)	*26 (38.8%)*	*12 (38.7%)*	*38 (38.8%)*
	Social anxiety, n (%)	*22 (32.8%)*	*11 (35.5%)*	*33 (33.7%)*
	Others thinking I'm reckless, n (%)	*13 (19.4%)*	*7 (22.6%)*	*20 (20.4%)*
	Other, n (%)	*21 (31.3%)*	*8 (25.8%)*	*29 (29.6%)*
4 (Q4)	How likely do you think you are to catch coronavirus at the event?			
	Missing, n (%)	1 (0.7%)	135 (60.3%)	136 (36.5%)

	Responders, n (%)	148 (99.3%)	89 (39.7%)	237 (63.5%)
	Very unlikely, n (%)	*63 (42.6%)*	*31 (34.8%)*	*94 (39.7%)*
	Fairly unlikely, n (%)	*64 (43.2%)*	*45 (50.6%)*	*109 (46.0%)*
	Neither unlikely nor likely, n (%)	*20 (13.5%)*	*11 (12.4%)*	*31 (13.1%)*
	Fairly likely, n (%)	*1 (0.7%)*	*2 (2.2%)*	*3 (1.3%)*
	Very likely, n (%)	*0 (0.0%)*	*0 (0.0%)*	*0 (0.0%)*
5 (Q7)	How concerned are you about potentially infecting others after attending the event?			
	Missing, n (%)	1 (0.7%)	136 (60.7%)	137 (36.7%)
	Responders, n (%)	148 (99.3%)	88 (39.3%)	236 (63.3%)
	Not at all concerned, n (%)	*63 (42.6%)*	*39 (44.3%)*	*102 (43.2%)*
	Slightly concerned, n (%)	*51 (34.5%)*	*24 (27.3%)*	*75 (31.8%)*
	Neither concerned nor unconcerned, n (%)	*9 (6.1%)*	*15 (17.0%)*	*24 (10.2%)*
	Moderately concerned, n (%)	*23 (15.5%)*	*7 (8.0%)*	*30(12.7%)*
	Very concerned, n (%)	*2 (1.4%)*	*3 (3.4%)*	*5 (2.1%)*
6 (Q8)	If 'not at all concerned' on last question, having stated that you are not concerned, share reasons why			
	Missing, n (%)	87 (58.4%)	185(82.6%)	272 (72.9%)
	Responders, n (%)	62 (41.6%)	39 (17.4%)	101 (27.1%)
	Because I've previously had Covid-19, n (%)	*9 (14.5%)*	*10 (25.6%)*	*19(18.8%)*
	Because I've been tested, n (%)	*39 (62.9%)*	*20 (51.3%)*	*59 (58.4%)*
	Because I've been vaccinated, n (%)	*37 (59.7%)*	*21 (53.8%)*	*58 (57.5%)*
	Because I've followed guidance on reducing the spread of Covid-19, n (%)	*43 (69.4%)*	*29 (74.4%)*	*72 (71.3%)*
	Other, n (%)	*4 (6.5%)*	*1 (2.6%)*	*5 (5.0%)*
7 (Q9)	How important do you think it is to resume these kinds of public events as soon as possible?			
	Missing, n (%)	1(0.7%)	135 (60.3%)	136 (36.5%)
	Responders, n (%)	148 (99.3%)	89 (39.7%)	237 (63.5%)
	Not at all important, n (%)	*0 (0.0%)*	*0 (0.0%)*	*0 (0.0%)*
	Slightly important, n (%)	*3 (2.0%)*	*1(1.1%)*	*4 (1.7%)*
	Neither important nor unimportant, n (%)	*2 (1.4%)*	*1 (1.1%)*	*3 (1.3%)*
	Moderately important, n (%)	*41 (27.7%)*	*22 (24.7%)*	*63 (26.6%)*
	Very important, n (%)	*102 (68.9%)*	*65 (73.0%)*	*167 (70.4%)*
8 (Q10)	In the past seven days, how often did you wash your hands with soap and water straight away after returning home from a public place?			
	Missing, n (%)	1 (0.7%)	135(60.3%)	136 (32.3%)
	Responders, n (%)	148 (99.3%)	89 (39.7%)	237 (67.7%)
	Always, n (%)	*94 (63.5%)*	*67 (75.3%)*	*161 (67.9%)*
	Often, n (%)	*44 (29.7%)*	*18 (20.2%)*	*62 (26.2%)*
	Sometimes, n (%)	*7 (4.7%)*	*2 (2.2%)*	*9 (3.8%)*
	Not very often, n (%)	*2 (1.4%)*	*2 (2.2%)*	*4 (1.7%)*
	Never, n (%)	*1 (0.7%)*	*0 (0.0%)*	*1 (0.4%)*
9 (Q11)	In the past seven days, have you used a face covering when outside your home to help slow the spread of the coronavirus (COVID-19)?			
	Missing, n (%)	2 (1.3%)	135 (60.3%)	137 (36.7%)
	Responders, n (%)	147 (98.7%)	89 (39.7%)	236 (63.3%)
	Yes, n (%)	*141 (95.3%)*	*84 (94.4%)*	*225 (95.3%)*
	No, n (%)	*5 (3.4%)*	*5 (5.6%)*	*10 (4.2%)*

	Not applicable, n (%)	*1 (0.7%)*	*0 (0.0%)*	*1 (0.4%)*
10 (Q12)	While you were inside a public space (e.g., shop, public transport) in the last seven days, how often did you wear a protective face covering to help slow the spread of the coronavirus (COVID-19)?			
	Missing, n (%)	2 (1.3%)	135 (60.3%)	137 (36.7%)
	Responders, n (%)	147 (98.7%)	89 (39.7%)	236 (63.3%)
	Always, n (%)	*140 (95.2%)*	*85 (95.5%)*	*225 (95.3%)*
	Often, n (%)	*5 (3.4%)*	*4 (4.5%)*	*9 (3.8%)*
	Sometimes, n (%)	*0 (0.0%)*	*0 (0.0%)*	*0 (0.0%)*
	Not very often, n (%)	*1 (0.7%)*	*0 (0.0%)*	*1 (0.4%)*
	Never, n (%)	*1 (0.7%)*	*0 (0.0%)*	*1 (0.4%)*
11 (Q13)	On average, how often do you follow the guidance on social distance when outside of support/childcare bubbles, maintaining 1-2 metres between yourself and other people?	2 (1.3%)	135 (60.3%)	137 (36.7%)
	Missing, n (%)	147 (98.7%)	89 (39.7%)	236 (63.3%)
	Responders, n (%)	*86 (58.5%)*	*51 (57.3%)*	*137 (58.1%)*
	Always, n (%)	*53 (36.0%)*	*32 (36.0%)*	*85 (36.0%)*
	Often, n (%)	*6 (4.1%)*	*5 (5.6%)*	*11 (4.7%)*
	Sometimes, n (%)	*2 (1.4%)*	*1 (1.1%)*	*3 (1.3%)*
	Not very often, n (%)	*0 (0.0%)*	*0 (0.0%)*	*0 (0.0%)*
	Never, n (%)			
12 (Q14)	In the past seven days, have you had any visitors inside your home from outside your support/childcare bubbles, including trades people, carers or medical staff?	138 (37.0%)	136 (60.7%)	138 (37.0%)
	Missing, n (%)	235 (63.0%)	88 (39.3%)	235 (63.0%)
	Responders, n (%)	*25 (17.0%)*	*20 (22.7%)*	*45 (19.1%)*
	Yes, n (%)	*122 (83.0%)*	*68 (77.3%)*	*190 (80.9%)*
	No, n (%)			
13 (Q15)	When you have had a visitor inside your home, which of the following actions did you take to reduce the spread of the coronavirus (COVID-19)?			
	Missing, n (%)	39 (26.2%)	151 (67.4%)	190 (50.9%)
	Responders, n (%)	110 (73.8%)	73 (32.6%)	183 (49.1%)
	Worn a face mask, n (%)	*42 (38.2%)*	*25 (34.2%)*	*67 (36.6%)*
	Asked the visitor to wear a mask, n (%)	*47 (42.7%)*	*24 (32.9%)*	*71 (38.8%)*
	Opened windows or doors, n (%)	*53 (48.2%)*	*47 (64.4%)*	*100 (54.6%)*
	Cleaned touch points, n (%)	*43 (39.1%)*	*37 (50.7%)*	*80 (43.7%)*
	Maintained social distancing, n (%)	*70 (63.6%)*	*52 (71.2%)*	*122 (66.7%)*
	Washed hands regularly, n (%)	*62 (56.4%)*	*46 (63.0%)*	*108 (59.0%)*
	Other, n (%)	*16 (14.5%)*	*6 (8.2%)*	*22 (12.0%)*
	None of the above, n (%)	*18 (16.4%)*	*9 (12.3%)*	*27 (14.8%)*
14 (Q16)	As part of your condition for attendance to this event, you will be required to complete a test. In case that this test results comes back negative, which statement below best describes what it means to you?			
	Missing, n (%)	1 (0.7%)	135 (60.3%)	136 (36.5%)
	Responders, n (%)	148 (99.3%)	89 (39.7%)	237 (63.5%)
	I am definitely not infectious, n (%)	*33 (22.3%)*	*18 (20.2%)*	*51 (21.5%)*

	I am probably not infectious, n (%)	*113 (76.4%)*	*68 (76.4%)*	*181 (76.4%)*
	I am probably infectious, n (%)	*0 (0.0%)*	*0 (0.0%)*	*0 (0.0%)*
	I am definitely infectious, n (%)	*1 (0.7%)*	*1 (1.1%)*	*2 (0.8%)*
	Don't know, n (%)	*1 (0.7%)*	*2 (2.2%)*	*3 (1.3%)*
15 (Q18)	What gender do you most identify with?			
	Missing, n (%)	2 (1.3%)	136 (60.7%)	138 (37.0%)
	Responders, n (%)	147 (98.7%)	88 (39.3%)	235 (63.0%)
	Man, n (%)	*69 (46.9%)*	*44 (50.0%)*	*113 (48.1%)*
	Woman, n (%)	*78 (53.1%)*	*43 (48.9%)*	*121 (51.5%)*
	Non-binary, n (%)	*0 (0.0%)*	*0 (0.0%)*	*0 (0.0%)*
	Other, n (%)	*0 (0.0%)*	*0 (0.0%)*	*0 (0.0%)*
	Prefer not to say, n (%)	*0 (0.0%)*	*1(1.1%)*	*1 (0.4%)*
16 (Q19)	Ethnicity			
	Missing, n (%)	1 (0.7%)	135 (60.3%)	136 (36.5%)
	Responders, n (%)	148 (99.3%)	89 (39.7%)	237 (63.5%)
	Asian/Asian-British- Indian, Pakistani, Bangladeshi, other, n (%)			
	Black/Black British- Caribbean. African, other, n (%)	*2 (1.4%)*	*1 (1.1%)*	*3 (1.3%)*
	Mixed race- White and Black/Black British, n (%)	*3 (2.0%)*	*3 (3.4%)*	*6 (2.5%)*
	Mixed race- other, n (%)	*2 (1.4%)*	*1 (1.1%)*	*3 (1.3%)*
	White- British, Irish, other, n (%)	*2 (1.4%)*	*0 (0.0%)*	*2 (0.8%)*
	Chinese/Chinese British, n (%)	*134 (90.5%)*	*84 (94.4%)*	*218 (92.0%)*
	Middle Eastern/Middle Eastern British- Arab, Turkish, other, n (%)	*1 (0.7%)*	*0 (0.0%)*	*1 (0.4%)*
	Other ethnic group, n (%)	*0 (0.0%)*	*0 (0.0%)*	*0 (0.0%)*
	Prefer not to say, n (%)	*1 (0.7%)*	*0 (0.0%)*	*1 (0.4%)*
		3 (2.0%)	*0 (0.0%)*	*3 (1.3%)*
17 (Q21)	Have you previously been diagnosed with Covid?			
	Missing, n (%)	2 (1.3%)	135 (60.3%)	137 (36.7%)
	Responders, n (%)	147 (98.7%)	89 (39.7%)	236 (63.3%)
	Yes, I've had a positive test (antibody or swab), n (%)	*15 (10.2%)*	*16 (18.0%)*	*31 (13.1%)*
	Yes, most likely but I've not had a test to confirm, n (%)	*15 (10.2%)*	*10 (11.2%)*	*25 (10.6%)*
	No, don't think I've had Covid-19, n (%)	*106 (72.1%)*	*53 (59.6%)*	*159 (67.4%)*
	Don't know, n (%)	*11 (7.5%)*	*10 (11.2%)*	*21 (8.9%)*
18 (Q23)	Have you received your vaccination?			
	Missing, n (%)	1 (0.7%)	135 (60.03)	136 (36.5%)
	Responders, n (%)	148 (99.3%)	89 (39.7%)	237 (63.5%)
	Yes, I have received my first vaccine dose, n (%)	*84 (56.8%)*	*41(46.1%)*	*125 (52.7%)*
	Yes, I have received both vaccine doses, n (%)	*17 (11.5%)*	*10 (11.2%)*	*27 (11.4%)*
	No, n (%)	*47 (31.7%)*	*38 (42.7%)*	*85 (35.9%)*
	Don't know, n (%)	*0 (0.0%)*	*0 (0.0%)*	*0 (0.0%)*

Pre-event screening questionnaire variables by ticket purchase (whether or not a pre-event questionnaire could be linked to a ticket) from Circus Nightclub

	Variable	Summary		
		Purchased a ticket	Not purchased a ticket	Overall
1	Number of participants (with completed pre-event screening questionnaire) n	7782	10822	18604

2 (Q2)	Are you concerned about attending the event?			
	Missing, n (%)	1135 (14.6%)	1376 (12.7%)	2511 (13.5%)
	Responders, n (%)	6647 (85.4%)	9446 (87.3%)	16093 (86.5%)
	Not at all concerned, n (%)	*5466(82.2%)*	*7567(80.1%)*	*13033(81.0%)*
	Slightly concerned, n (%)	*834 (12.6%)*	*1172 (12.4%)*	*2006 (12.5%)*
	Moderately concerned, n (%)	*260 (3.9%)*	*453 (4.8%)*	*713 (4.4%)*
	Very concerned, n (%)	*87 (1.3%)*	*254 (2.7%)*	*341 (2.1%)*
3 (Q3)	Are there any factors that might contribute to some of your concern about attending the event?			
	Missing, n (%)	6643(85.4%)	9031 (83.5%)	15674 (84.2%)
	Responders, n (%)	1139 (14.6%)	1791 (13.6%)	2930 (15.8%)
	Possibly catching Covid-19, n (%)	*632 (55.5%)*	*1006 (56.2%)*	*1638(55.9%)*
	Impact of having to self-isolate if I test positive for Covid-19, n (%)	*424 (37.2%)*	*667 (37.2%)*	*1091 (37.2%)*
	Social anxiety, n (%)	*297 (26.1%)*	*466 (26.0%)*	*763 (26.0%)*
	Others thinking I'm reckless, n (%)	*396 (34.8%)*	*562 (31.4%)*	*958 (32.7%)*
	Other, n (%)	*424 (37.2%)*	*621 (34.7%)*	*1045 (35.7%)*
4 (Q4)	How likely do you think you are to catch coronavirus at the event?			
	Missing, n (%)	1155 (14.8%)	1414 (13.1%)	2569 (13.8%)
	Responders, n (%)	6627 (85.2%)	9408 (86.9%)	16035 (86.2%)
	Very unlikely, n (%)	*3180 (48.0%)*	*4572 (48.6%)*	*7752 (48.3%)*
	Fairly unlikely, n (%)	*2402 (36.3%)*	*2944 (31.3%)*	*5346 (33.3%)*
	Neither unlikely nor likely, n (%)	*861 (13.0%)*	*1471 (15.6%)*	*2332 (14.5%)*
	Fairly likely, n (%)	*149 (2.2%)*	*303 (3.2%)*	*452 (2.8%)*
	Very likely, n (%)	*35 (0.5%)*	*118 (1.3%)*	*153 (1.0%)*
5 (Q7)	How concerned are you about potentially infecting others after attending the event?			
	Missing, n (%)	1160 (14.9%)	1468 (13.6%)	2628 (14.1%)
	Responders, n (%)	6622 (85.1%)	9354 (86.4%)	15976 (85.9%)
	Not at all concerned, n (%)	*3437 (51.9%)*	*4889 (52.3%)*	*8326 (52.1%)*
	Slightly concerned, n (%)	*1464 (22.1%)*	*1732 (18.5%)*	*3196 (20.0%)*
	Neither concerned nor unconcerned, n (%)	*1059 (16.0%)*	*1550 (16.6%)*	*2609 (16.3%)*
	Moderately concerned, n (%)	*537 (8.1%)*	*884 (9.5%)*	*1421 (8.9%)*
	Very concerned, n (%)	*125 (1.9%)*	*299 (3.2%)*	*424 (2.7%)*
6 (Q8)	If 'not at all concerned' on last question, having stated that you are not concerned, share reasons why			
	Missing, n (%)	4397 (56.5%)	6061 (56.0%)	10458 (56.2%)
	Responders, n (%)	3385 (43.5%)	4761 (44.0%)	8146 (43.8%)
	Because I've previously had Covid-19, n (%)	*1252 (37.0%)*	*1495 (31.4%)*	*2747 (33.7%)*
	Because I've been tested, n (%)	*1582 (46.7%)*	*2039 (42.8%)*	*3621 (44.5%)*
	Because I've been vaccinated, n (%)	*797 (23.5%)*	*1202 (25.2%)*	*1999 (24.5%)*
	Because I've followed guidance on reducing the spread of Covid-19, n (%)			
	Other, n (%)	*1966 (58.1%)*	*2580 (54.2%)*	*4546 (55.8%)*
		86 (2.5%)	*215 (4.5%)*	*301 (3.7%)*
7 (Q9)	How important do you think it is to resume these kinds of public events as soon as possible?			
	Missing, n (%)	1156 (14.9%)	1485 (13.7%)	2641 (14.2%)
	Responders, n (%)	6626 (85.1%)	9337 (86.3%)	15963 (85.8%)
	Not at all important, n (%)	*117 (1.8%)*	*308 (3.3%)*	*425 (2.7%)*

	Slightly important, n (%)	*132 (2.0%)*	*207 (2.2%)*	*339 (2.1%)*
	Neither important nor unimportant, n (%)	*238 (3.6%)*	*482 (5.2%)*	*720 (4.5%)*
	Moderately important, n (%)	*1207 (18.2%)*	*1608 (17.2%)*	*2815 (17.6%)*
	Very important, n (%)	*4932 (74.4%)*	*6732 (72.1%)*	*11664 (73.1%)*
8 (Q10)	In the past seven days, how often did you wash your hands with soap and water straight away after returning home from a public place?			
	Missing, n (%)	1149 (14.8%)	1569 (14.5%)	2718 (14.6%)
	Responders, n (%)	6633 (85.2%)	9253 (85.5%)	15886 (85.4%)
	Always, n (%)	*5217 (78.7%)*	*7167 (77.5%)*	*12384 (78.0%)*
	Often, n (%)	*1107 (16.7%)*	*1511 (16.3%)*	*2618 (16.5%)*
	Sometimes, n (%)	*207 (3.1%)*	*359 (3.9%)*	*566 (3.6%)*
	Not very often, n (%)	*50 (0.7%)*	*97 (1.0%)*	*147 (0.9%)*
	Never, n (%)	*52 (0.8%)*	*119 (1.3%)*	*171 (1.1%)*
9 (Q11)	In the past seven days, have you used a face covering when outside your home to help slow the spread of the coronavirus (COVID-19)?			
	Missing, n (%)	1161 (14.9%)	1607 (14.8%)	2768 (14.9%)
	Responders, n (%)	6621 (85.1%)	9215 (85.2%)	15836 (85.2%)
	Yes, n (%)	*6346 (95.8%)*	*8491 (92.1%)*	*14837 (93.7%)*
	No, n (%)	*166 (2.5%)*	*422 (4.6%)*	*588 (3.7%)*
	Not applicable, n (%)	*109 (1.7%)*	*302 (3.3%)*	*411 (2.6%)*
10 (Q12)	While you were inside a public space (e.g., shop, public transport) in the last seven days, how often did you wear a protective face covering to help slow the spread of the coronavirus (COVID-19)?			
	Missing, n (%)	1161 (14.9%)	1605 (14.8%)	2766 (14.9%)
	Responders, n (%)	6621 (85.1%)	9217 (85.2%)	15838 (85.1%)
	Always, n (%)	*6026 (91.0%)*	*8033 (87.2%)*	*14059 (88.8%)*
	Often, n (%)	*368 (5.6%)*	*627 (6.8%)*	*995 (6.3%)*
	Sometimes, n (%)	*132 (2.0%)*	*284 (3.1%)*	*416 (2.6%)*
	Not very often, n (%)	*33 (0.5%)*	*93 (1.0%)*	*126 (0.8%)*
	Never, n (%)	*62 (0.9%)*	*180 (1.9%)*	*242 (1.5%)*
11 (Q13)	On average, how often do you follow the guidance on social distance when outside of support/childcare bubbles, maintaining 1-2 metres between yourself and other people?			
	Missing, n (%)	1153 (14.8%)	1606 (14.8%)	2759 (14.8%)
	Responders, n (%)	6629 (85.2%)	9216 (85.2%)	15845 (85.2%)
	Always, n (%)	*4526 (68.3%)*	*6148 (66.7%)*	*10674 (67.4%)*
	Often, n (%)	*1586 (23.9%)*	*2127 (23.1%)*	*3713 (23.4%)*
	Sometimes, n (%)	*370 (5.6%)*	*599 (6.5%)*	*969 (6.1%)*
	Not very often, n (%)	*95 (1.4%)*	*192 (2.1%)*	*287 (1.8%)*
	Never, n (%)	*52 (0.8%)*	*150 (1.6%)*	*202 (1.3%)*
12 (Q14)	In the past seven days, have you had any visitors inside your home from outside your support/childcare bubbles, including trades people, carers or medical staff?			
	Missing, n (%)	1207 (15.5%)	1710 (15.8%)	2917 (15.7%)
	Responders, n (%)	6575 (84.5%)	9112 (84.2%)	15687 (84.3%)
	Yes, n (%)	*861 (13.1%)*	*1316 (14.4%)*	*2177 (13.9%)*

	No, n (%)	*5714 (86.9%)*	*7796 (85.6%)*	*13510 (86.1%)*
13 (Q15)	When you have had a visitor inside your home, which of the following actions did you take to reduce the spread of the coronavirus (COVID-19)?			
	Missing, n (%)	1837 (23.6%)	2661 (24.6%)	4498 (24.2%)
	Responders, n (%)	5945 (76.4%)	8161 (62.0%)	14106 (75.8%)
	Worn a face mask, n (%)	*3120 (52.5%)*	*3901 (47.8%)*	*7021 (49.8%)*
	Asked the visitor to wear a mask, n (%)	*2376 (40.0%)*	*2827 (34.6%)*	*5203 (36.9%)*
	Opened windows or doors, n (%)	*3037 (51.1%)*	*3685 (45.2%)*	*6722 (47.7%)*
	Cleaned touch points, n (%)	*2162 (36.4%)*	*2705 (33.1%)*	*4867 (34.5%)*
	Maintained social distancing, n (%)	*3840 (64.6%)*	*4789 (58.7%)*	*8629 (61.2%)*
	Washed hands regularly, n (%)	*3227 (54.3%)*	*4014 (49.2%)*	*7241 (51.3%)*
	Other, n (%)	*144 (2.4%)*	*232 (2.8%)*	*376 (2.7%)*
	None of the above, n (%)	*1018 (17.1%)*	*1649 (20.2%)*	*2667 (18.9%)*
14 (Q16)	As part of your condition for attendance to this event, you will be required to complete a test. In case that this test results comes back negative, which statement below best describes what it means to you?			
	Missing, n (%)	1153 (14.8%)	1641 (15.2%)	2794 (15.0%)
	Responders, n (%)	6629 (85.2%)	9181 (84.8%)	15810 (85.0%)
	I am definitely not infectious, n (%)	*3480 (52.5%)*	*5102 (55.5%)*	*8582 (54.3%)*
	I am probably not infectious, n (%)	*2803 (42.3%)*	*3394 (37.0%)*	*6197 (39.2%)*
	I am probably infectious, n (%)	*86 (1.3%)*	*137 (1.5%)*	*223 (1.4%)*
	I am definitely infectious, n (%)	*68 (1.0%)*	*174 (1.9%)*	*242 (1.5%)*
	Don't know, n (%)	*192 (2.9%)*	*374 (4.1%)*	*566 (3.6%)*
15 (Q18)	What gender do you most identify with?			
	Missing, n (%)	1147 (14.7%)	1696 (15.7%)	2843 (15.3%)
	Responders, n (%)	6635 (85.3%)	9126 (84.3%)	15761 (84.7%)
	Man, n (%)	*3307 (49.9%)*	*4512 (49.4%)*	*7819 (49.6%)*
	Woman, n (%)	*3246 (48.9%)*	*4339 (47.6%)*	*7585 (48.1%)*
	Non-binary, n (%)	*31 (0.47%)*	*111 (1.2%)*	*142 (0.9%)*
	Other, n (%)	*2 (0.03%)*	*18 (0.2%)*	*20 (0.1%)*
	Prefer not to say, n (%)	*49 (0.7%)*	*146 (1.6%)*	*195 (1.2%)*
16 (Q19)	Ethnicity			
	Missing, n (%)	1153 (14.8%)	1728 (16.0%)	2881 (15.5%)
	Responders, n (%)	6629 (85.2%)	9094 (84.0%)	15723 (84.5%)
	Asian/Asian-British- Indian, Pakistani, Bangladeshi, other,	*127 (1.9%)*	*188 (2.1%)*	*315 (2.0%)*
	n (%)	*54 (0.8%)*	*114 (1.3%)*	*168 (1.1%)*
	Black/Black British- Caribbean. African, other, n (%)	*146 (2.2%)*	*183 (2.0%)*	*329 (2.1%)*
	Mixed race- White and Black/Black British, n (%)	*103 (1.6%)*	*130 (1.4%)*	*233 (1.5%)*
	Mixed race- other, n (%)	*6047 (91.2%)*	*8131 (89.4%)*	*14178 (90.2%)*
	White- British, Irish, other, n (%)	*25 (0.4%)*	*72 (0.8%)*	*97 (0.6%)*
	Chinese/Chinese British, n (%)			
	Middle Eastern/Middle Eastern British- Arab, Turkish,	*30 (0.5%)*	*46 (0.5%)*	*76 (0.5%)*
	other, n (%)	*20 (0.3%)*	*19 (0.2%)*	*39 (0.2%)*
	Other ethnic group, n (%)	*77 (1.2%)*	*211 (2.3%)*	*288 (1.8%)*
	Prefer not to say, n (%)			
17 (Q21)	Have you previously been diagnosed with Covid?			
	Missing, n (%)	1149 (14.8%)	1729 (16.0%)	2878 (15.5%)
	Responders, n (%)	6633 (85.2%)	9093 (84.0%)	15726 (84.5%)

	Yes, I've had a positive test (antibody or swab), n (%)	*1982 (29.8%)*	*2364 (26.0%)*	*4346 (27.7%)*
	Yes, most likely but I've not had a test to confirm, n (%)	*1185 (17.9%)*	*1319 (14.5%)*	*2504 (15.9%)*
	No, don't think I've had Covid-19, n (%)	*3016 (45.5%)*	*4599 (50.6%)*	*7615 (48.4%)*
	Don't know, n (%)	*450 (6.8%)*	*811 (8.9%)*	*1261 (8.0%)*
18 (Q23)	Have you received your vaccination? Missing, n (%)	1172 (15.1%)	1779 (16.4%)	2951 (15.9%)
	Responders, n (%)	6610 (84.9%)	9043 (83.6%)	15653 (84.2%)
	Yes, I have received my first vaccine dose, n (%)	*996 (15.0%)*	*1407 (15.6%)*	*2403 (15.4%)*
	Yes, I have received both vaccine doses, n (%)	*562 (8.5%)*	*797 (8.8%)*	*1359 (8.7%)*
	No, n (%)	*4957 (75.0%)*	*6583 (72.8%)*	*11540 (73.7%)*
	Don't know, n (%)	*95 (1.5%)*	*256 (2.8%)*	*351 (2.2%)*

Pre-event screening questionnaire variables by attendance (whether the linked ticket was scanned or not) from Circus Nightclub

	Variable	Summary		
		Tickets scanned	Tickets not scanned	Overall
1	Number of participants (with completed pre-event screening questionnaire)	6802	980	7782
2 (Q2)	Are you concerned about attending the event?			
	Missing, n (%)	692 (10.2%)	443 (45.2%)	1135 (14.6%)
	Responders, n (%)	6110 (89.8%)	537 (54.8%)	6647 (85.4%)
	Not at all concerned, n (%)	5047 (82.6%)	419 (78.0%)	5466 (82.2%)
	Slightly concerned, n (%)	748 (12.2%)	86 (16.0%)	834 (12.6%)
	Moderately concerned, n (%)	237 (3.9%)	23 (4.3%)	260 (3.9%)
	Very concerned, n (%)	78 (1.3%)	9 (1.7%)	87 (1.3%)
3 (Q3)	Are there any factors that might contribute to some of your concern about attending the event?			
	Missing, n (%)	5777(84.9%)	866(88.4%)	6643(85.4%)
	Responders, n (%)	1025 (15.1%)	114 (11.6%)	1139 (14.6%)
	Possibly catching Covid-19, n (%)	*583 (56.9%)*	*49 (43.0%)*	*632 (55.5%)*
	Impact of having to self-isolate if I test positive for Covid-19, n (%)	*387 (37.8%)*	*37 (32.5%)*	*424 (37.2%)*
	Social anxiety, n (%)	*260 (25.4%)*	*37 (32.5%)*	*297 (26.1%)*
	Others thinking I'm reckless, n (%)	*340 (33.2%)*	*56 (49.1%)*	*396 (34.8%)*
	Other, n (%)	*366 (35.7%)*	*58 (50.9%)*	*424 (37.2%)*
4 (Q4)	How likely do you think you are to catch coronavirus at the event?			
	Missing, n (%)	712 (10.5%)	443 (45.2%)	1155 (14.8%)
	Responders, n (%)	6090 (89.5%)	537 (54.8%)	6627 (85.2%)
	Very unlikely, n (%)	*2927 (48.1%)*	*253 (47.1%)*	*3180 (48.0%)*
	Fairly unlikely, n (%)	*2201 (36.1%)*	*201 (37.4%)*	*2402 (36.3%)*
	Neither unlikely nor likely, n (%)	*787 (12.9%)*	*74 (13.8%)*	*861 (13.0%)*
	Fairly likely, n (%)	*140 (2.3%)*	*9 (1.7%)*	*149 (2.2%)*
	Very likely, n (%)	*35 (0.6%)*	*0 (0.0%)*	*35 (0.5%)*
5 (Q7)	How concerned are you about potentially infecting others after attending the event?			
	Missing, n (%)	716 (10.5%)	444 (45.3%)	1160 (14.9%)
	Responders, n (%)	6086 (89.5%)	536 (54.7%)	6622 (85.1%)

	Not at all concerned, n (%)	*3145 (51.7%)*	*292 (54.5%)*	*3437 (51.9%)*
	Slightly concerned, n (%)	*1355 (22.3%)*	*109 (20.3%)*	*1464 (22.1%)*
	Neither concerned nor unconcerned, n (%)	*971 (15.9%)*	*88 (16.4%)*	*1059 (16.0%)*
	Moderately concerned, n (%)	*498 (8.2%)*	*39 (7.3%)*	*537 (8.1%)*
	Very concerned, n (%)	*117 (1.9%)*	*8 (1.5%)*	*125 (1.9%)*
6 (Q8)	If 'not at all concerned' on last question, having stated that you are not concerned, share reasons why			
	Missing, n (%)	3705 (54.5%)	692 (70.6%)	4397 (56.5%)
	Responders, n (%)	3097 (45.5%)	288 (29.4%)	3385 (43.5%)
	Because I've previously had Covid-19, n (%)	*1160 (37.5%)*	*92 (31.9%)*	*1252 (37.0%)*
	Because I've been tested, n (%)	*1450 (46.8%)*	*132 (45.8%)*	*1582 (46.7%)*
	Because I've been vaccinated, n (%)	*718 (23.2%)*	*79 (27.4%)*	*797 (23.5%)*
	Because I've followed guidance on reducing the spread of Covid-19, n (%)	*1796 (58.0%)*	*170 (59.0%)*	*1966 (58.1%)*
	Other, n (%)	*79(2.6%)*	*7 (2.4%)*	*86 (2.5%)*
7 (Q9)	How important do you think it is to resume these kinds of public events as soon as possible?			
	Missing, n (%)	712 (10.5%)	444 (45.3%)	1156 (14.9%)
	Responders, n (%)	6090 (89.5%)	536 (54.7%)	6626 (85.1%)
	Not at all important, n (%)	*105 (1.7%)*	*12 (2.2%)*	*117 (1.8%)*
	Slightly important, n (%)	*122 (2.0%)*	*10 (1.9%)*	*132 (2.0%)*
	Neither important nor unimportant, n (%)	*218 (3.6%)*	*20 (3.7%)*	*238 (3.6%)*
	Moderately important, n (%)	*1096 (18.0%)*	*111 (20.7%)*	*1207 (18.2%)*
	Very important, n (%)	*4549 (74.7%)*	*383 (71.5%)*	*4932 (74.4%)*
8 (Q10)	In the past seven days, how often did you wash your hands with soap and water straight away after returning home from a public place?			
	Missing, n (%)	707 (10.4%)	442 (45.1%)	1149 (14.8%)
	Responders, n (%)	6095 (89.6%)	538 (54.9%)	6633 (85.2%)
	Always, n (%)	*4803 (78.8%)*	*414 (77.0%)*	*5217 (78.7%)*
	Often, n (%)	*1004 (16.5%)*	*103 (19.1%)*	*1107 (16.7%)*
	Sometimes, n (%)	*195 (3.2%)*	*12 (2.2%)*	*207 (3.1%)*
	Not very often, n (%)	*45 (0.7%)*	*5 (0.9%)*	*50 (0.7%)*
	Never, n (%)	*48 (0.8%)*	*4 (0.7%)*	*52 (0.8%)*
9 (Q11)	In the past seven days, have you used a face covering when outside your home to help slow the spread of the coronavirus (COVID-19)?			
	Missing, n (%)	716 (10.5%)	445 (45.4%)	1161 (14.9%)
	Responders, n (%)	6086 (89.5%)	535 (54.6%)	6621 (85.1%)
	Yes, n (%)	*5839 (95.9%)*	*507 (94.8%)*	*6346 (95.9%)*
	No, n (%)	*150 (2.5%)*	*16 (3.0%)*	*166 (2.5%)*
	Not applicable, n (%)	*97 (1.6%)*	*12 (2.2%)*	*109 (1.6%)*
10 (Q12)	While you were inside a public space (e.g., shop, public transport) in the last seven days, how often did you wear a protective face covering to help slow the spread of the coronavirus (COVID-19)?			
	Missing, n (%)	715 (10.5%)	446 (45.5%)	1161 (14.9%)
	Responders, n (%)	6087 (89.5%)	534 (54.5%)	6621 (85.1%)
	Always, n (%)	*5552 (91.2%)*	*474 (88.8%)*	*6026 (91.0%)*
	Often, n (%)	*329 (5.4%)*	*39 (7.3%)*	*368 (5.6%)*
	Sometimes, n (%)	*121 (2.0%)*	*11 (2.0%)*	*132 (2.0%)*

	Not very often, n (%)	*30 (0.5%)*	*3 (0.6%)*	*33 (0.5%)*
	Never, n (%)	*55 (0.9%)*	*7 (1.3%)*	*62 (0.9%)*
11 (Q13)	On average, how often do you follow the guidance on social distance when outside of support/childcare bubbles, maintaining 1-2 metres between yourself and other people?	710 (10.4%)	443 (45.2%)	1153 (14.8%)
	Missing, n (%)	6092 (89.6%)	537 (54.8%)	6629 (85.2%)
	Responders, n (%)	*4152(68.2%)*	*374 (69.7%)*	*4526 (68.3%)*
	Always, n (%)	*1458 (23.9%)*	*128 (23.8%)*	*1586 (23.9%)*
	Often, n (%)	*346 (5.7%)*	*24 (4.5%)*	*370 (5.6%)*
	Sometimes, n (%)	*89 (1.4%)*	*6 (1.1%)*	*95 (1.4%)*
	Not very often, n (%)	*47 (0.8%)*	*5 (0.9%)*	*52 (0.8%)*
	Never, n (%)			
12 (Q14)	In the past seven days, have you had any visitors inside your home from outside your support/childcare bubbles, including trades people, carers or medical staff? Missing,	760 (11.2%)	447 (45.6%)	1207 (15.5%)
	n (%)	6042 (88.8%)	533 (54.4%)	6575 (84.5%)
	Responders, n (%)	*787 (13.0%)*	*74 (13.9%)*	*861 (13.1%)*
	Yes, n (%)	*5255 (87.0%)*	*459 (86.1%)*	*5714 (86.9%)*
	No, n (%)			
13 (Q15)	When you have had a visitor inside your home, which of the following actions did you take to reduce the spread of the coronavirus (COVID-19)?			
	Missing, n (%)	1356 (19.9%)	481 (49.1%)	1837 (23.6%)
	Responders, n (%)	5446 (80.1%)	499 (50.9%)	5945 (76.4%)
	Worn a face mask, n (%)	*2856 (52.4%)*	*264 (52.9%)*	*3120 (52.5%)*
	Asked the visitor to wear a mask, n (%)	*2165 (39.8%)*	*211 (42.3%)*	*2376 (40.0%)*
	Opened windows or doors, n (%)	*2786 (51.2%)*	*251 (50.3%)*	*3037 (51.1%)*
	Cleaned touch points, n (%)	*1985 (36.4%)*	*177 (35.5%)*	*2162 (36.4%)*
	Maintained social distancing, n (%)	*3524 (64.7%)*	*316 (63.3%)*	*3840 (64.6%)*
	Washed hands regularly, n (%)	*2960 (54.4%)*	*267 (53.5%)*	*3227 (54.3%)*
	Other, n (%)	*131 (2.4%)*	*13 (2.6%)*	*144 (2.4%)*
	None of the above, n (%)	*924 (17.0%)*	*94 (18.8%)*	*1018 (17.1%)*
14 (Q16)	As part of your condition for attendance to this event, you will be required to complete a test. In case that this test results comes back negative, which statement below best describes what it means to you?			
	Missing, n (%)	711 (10.5%)	442 (45.1%)	1153 (14.8%)
	Responders, n (%)	6091 (89.5%)	538 (54.9%)	6629 (85.2%)
	I am definitely not infectious, n (%)	*3209 (52.7%)*	*271 (50.4%)*	*3480 (52.5%)*
	I am probably not infectious, n (%)	*2561 (42.1%)*	*242 (45.0%)*	*2803 (42.3%)*
	I am probably infectious, n (%)	*80 (1.3%)*	*6 (1.1%)*	*86 (1.3%)*
	I am definitely infectious, n (%)	*64 (1.0%)*	*4 (0.7%)*	*68 (1.0%)*
	Don't know, n (%)	*177 (2.9%)*	*15 (2.8%)*	*192 (2.9%)*
15 (Q18)	What gender do you most identify with?			
	Missing, n (%)	706 (10.4%)	441 (45.0%)	1147 (14.7%)
	Responders, n (%)	6096 (89.6%)	539 (55.0%)	6635 (85.3%)
	Man, n (%)	*3049 (50.0%)*	*258 (47.9%)*	*3307 (49.9%)*
	Woman, n (%)	*2970 (48.7%)*	*276 (51.2%)*	*3246 (48.9%)*
	Non-binary, n (%)	*29 (0.47%)*	*2 (0.4%)*	*31 (0.47%)*

	Other, n (%)	*2 (0.03%)*	*0 (0.0%)*	*2 (0.03%)*
	Prefer not to say, n (%)	*46 (0.8%)*	*3 (0.5%)*	*49 (0.7%)*
16 (Q19)	Ethnicity			
	Missing, n (%)	711 (10.5%)	442 (45.1%)	1153 (14.8%)
	Responders, n (%)	6091 (89.5%)	538 (55.0%)	6629 (85.2%)
	Asian/Asian-British- Indian, Pakistani, Bangladeshi, other, n (%)			
	Black/Black British- Caribbean. African, other, n (%)	*117 (1.9%)*	*10 (1.9%)*	*127 (1.9%)*
	Mixed race- White and Black/Black British, n (%)	*47 (0.8%)*	*7 (1.3%)*	*54 (0.8%)*
	Mixed race- other, n (%)	*132 (2.2%)*	*14 (2.6%)*	*146 (2.2%)*
	White- British, Irish, other, n (%)	*97 (1.6%)*	*6 (1.1%)*	*103 (1.6%)*
	Chinese/Chinese British, n (%)	*5556 (91.2%)*	*491 (91.3%)*	*6047 (91.2%)*
	Middle Eastern/Middle Eastern British- Arab, Turkish, other, n (%)	*23 (0.4%)*	*2 (0.4%)*	*25 (0.4%)*
	Other ethnic group, n (%)	*28 (0.5%)*	*2 (0.4%)*	*30 (0.5%)*
	Prefer not to say, n (%)	*18 (0.3%)*	*2 (0.4%)*	*20 (0.3%)*
		73 (1.2%)	*4 (0.7%)*	*77 (1.2%)*
17 (Q21)	Have you previously been diagnosed with Covid?			
	Missing, n (%)	707 (10.4%)	442 (45.1%)	1149 (14.8%)
	Responders, n (%)	6095 (89.6%)	538 (54.9%)	6633 (85.2%)
	Yes, I've had a positive test (antibody or swab), n (%)	*1823 (29.9%)*	*159 (29.5%)*	*1982 (29.8%)*
	Yes, most likely but I've not had a test to confirm, n (%)	*1092 (17.9%)*	*93 (17.3%)*	*1185 (17.9%)*
	No, don't think I've had Covid-19, n (%)	*2765 (45.4%)*	*251 (46.7%)*	*3016 (45.5%)*
	Don't know, n (%)	*415 (6.8%)*	*35 (6.5%)*	*450 (6.8%)*
18 (Q23)	Have you received your vaccination?			
	Missing, n (%)	728 (10.7%)	444 (45.3%)	1172 (15.1%)
	Responders, n (%)	6074 (89.3%)	536 (54.7%)	6610 (84.9%)
	Yes, I have received my first vaccine dose, n (%)	*911 (15.0%)*	*85 (15.8%)*	*996 (15.1%)*
	Yes, I have received both vaccine doses, n (%)	*517 (8.5%)*	*45 (8.4%)*	*562 (8.5%)*
	No, n (%)	*4559 (75.1%)*	*398 (74.3%)*	*4957 (75.0%)*
	Don't know, n (%)	*87 (1.4%)*	*8 (1.5%)*	*95 (1.4%)*

Pre-event screening questionnaire variables by ticket purchase (whether or not a pre-event questionnaire could be linked to a ticket) from Sefton Park

	Variable	Summary		
		Purchased a ticket	Not purchased a ticket	Overall
1	Number of participants (with completed pre-event screening questionnaire)	7011	10990	18001
2 (Q2)	Are you concerned about attending the event?			
	Missing, n (%)	580 (8.3%)	1667 (15.2%)	2247 (12.5%)
	Responders, n (%)	6431 (91.7%)	9323 (84.8%)	15754(87.5%)
	Not at all concerned, n (%)	*4766 (74.1%)*	*6682 (71.7%)*	*11448 (72.7%)*
	Slightly concerned, n (%)	*1328 (20.6%)*	*1917 (20.6%)*	*3245 (20.6%)*
	Moderately concerned, n (%)	*300 (4.7%)*	*601 (6.4%)*	*901 (5.7%)*
	Very concerned, n (%)	*37 (0.6%)*	*123 (1.3%)*	*160 (1.0%)*

3 (Q3)	Are there any factors that might contribute to some of your concern about attending the event?			
	Missing, n (%)	5387(76.8%)	8462 (77.0%)	13849 (76.9%)
	Responders, n (%)	1624 (23.2%)	2528 (23.0%)	4152 (23.1%)
	Possibly catching Covid-19, n (%)	*1036 (63.8%)*	*1598 (63.2%)*	*2634 (63.4%)*
	Impact of having to self-isolate if I test positive for Covid-19, n (%)	*708 (43.6%)*	*1021 (40.4%)*	*1729 (41.6%)*
	Social anxiety, n (%)	*444 (27.3%)*	*722 (28.6%)*	*1166 (28.1%)*
	Others thinking I'm reckless, n (%)	*643 (39.6%)*	*1039 (41.1%)*	*1682 (40.5%)*
	Other, n (%)	*681 (41.9%)*	*1112 (44.0%)*	*1793 (43.2%)*
4 (Q4)	How likely do you think you are to catch coronavirus at the event?			
	Missing, n (%)	589 (8.4%)	1691 (15.4%)	2280 (12.7%)
	Responders, n (%)	6422 (91.6%)	9299 (84.6%)	15721 (87.3%)
	Very unlikely, n (%)	*2421 (37.7%)*	*3573 (38.4%)*	*5994 (38.1%)*
	Fairly unlikely, n (%)	*2907 (45.3%)*	*3978 (42.8%)*	*6885 (43.8%)*
	Neither unlikely nor likely, n (%)	*922 (14.4%)*	*1354 (14.6%)*	*2276 (14.5%)*
	Fairly likely, n (%)	*156 (2.4%)*	*317 (3.4%)*	*473 (3.0%)*
	Very likely, n (%)	*16 (2.5%)*	*77 (0.8%)*	*93 (0.6%)*
5 (Q7)	How concerned are you about potentially infecting others after attending the event?			
	Missing, n (%)	595 (8.5%)	1729 (15.7%)	2324 (12.9%)
	Responders, n (%)	6416 (91.5%)	9261 (84.3%)	15677 (87.1%)
	Not at all concerned, n (%)	*2942 (45.9%)*	*4286 (46.3%)*	*7228 (46.1%)*
	Slightly concerned, n (%)	*1776 (27.7%)*	*2467 (26.6%)*	*4243 (27.1%)*
	Neither concerned nor unconcerned, n (%)	*914 (14.2%)*	*1277 (13.8%)*	*2191 (14.0%)*
	Moderately concerned, n (%)	*694 (10.8%)*	*995 (10.7%)*	*1689 (10.8%)*
	Very concerned, n (%)	*90 (1.4%)*	*236 (2.5%)*	*326 (2.1%)*
6 (Q8)	If 'not at all concerned' on last question, having stated that you are not concerned, share reasons why			
	Missing, n (%)	4090 (58.3%)	6790 (61.8%)	10880 (60.4%)
	Responders, n (%)	2921 (41.7%)	4200 (38.2%)	7121 (39.6%)
	Because I've previously had Covid-19, n (%)	*1064 (36.4%)*	*1354 (32.2%)*	*2418 (34.0%)*
	Because I've been tested, n (%)	*1527 (52.3%)*	*2226 (53.0%)*	*3753 (52.7%)*
	Because I've been vaccinated, n (%)	*972 (33.3%)*	*1482 (35.3%)*	*2454 (34.5%)*
	Because I've followed guidance on reducing the spread of Covid-19, n (%)	*1899 (65.0%)*	*2629 (62.6%)*	*4528 (63.6%)*
	Other, n (%)	*110 (3.8%)*	*156 (3.7%)*	*266 (3.7%)*
7 (Q9)	How important do you think it is to resume these kinds of public events as soon as possible?			
	Missing, n (%)	591 (8.4%)	1726 (15.7%)	2317 (12.9%)
	Responders, n (%)	6420 (91.6%)	9264 (84.3%)	15684 (87.1%)
	Not at all important, n (%)	*46 (0.7%)*	*164 (1.8%)*	*210 (1.3%)*
	Slightly important, n (%)	*146 (2.3%)*	*271 (2.9%)*	*417 (2.7%)*
	Neither important nor unimportant, n (%)	*185 (2.9%)*	*312 (3.4%)*	*497 (3.2%)*
	Moderately important, n (%)	*1498 (23.3%)*	*2014 (21.7%)*	*3512 (22.4%)*
	Very important, n (%)	*4545 (70.8%)*	*6503 (70.2%)*	*11048 (70.4%)*
8 (Q10)	In the past seven days, how often did you wash your hands with soap and water straight away after returning home from a public place?			
	Missing, n (%)	586 (8.4%)	1837 (16.7%)	2423 (13.5%)

	Responders, n (%)	6425 (91.6%)	9153 (83.3%)	15578 (86.5%)
	Always, n (%)	*4725 (73.5%)*	*6763 (73.9%)*	*11488 (73.7%)*
	Often, n (%)	*1388 (21.6%)*	*1895 (20.7%)*	*3283 (21.1%)*
	Sometimes, n (%)	*212 (3.3%)*	*313 (3.4%)*	*525 (3.4%)*
	Not very often, n (%)	*64 (1.0%)*	*109 (1.2%)*	*173 (1.1%)*
	Never, n (%)	*36 (0.6%)*	*73 (0.8%)*	*109 (0.7%)*
9 (Q11)	In the past seven days, have you used a face covering when outside your home to help slow the spread of the coronavirus (COVID-19)?			
	Missing, n (%)	597 (8.5%)	1866 (17.0%)	2463 (13.7%)
	Responders, n (%)	6414 (91.5%)	9124 (83.0%)	15538 (86.3%)
	Yes, n (%)	*6238 (97.3%)*	8728 (95.7%)	*14966 (96.3%)*
	No, n (%)	*140 (2.2%)*	294 (3.2%)	*434 (2.8%)*
	Not applicable, n (%)	*36 (0.6%)*	102 (1.1%)	*138 (0.9%)*
10 (Q12)	While you were inside a public space (e.g., shop, public transport) in the last seven days, how often did you wear a protective face covering to help slow the spread of the coronavirus (COVID-19)?			
	Missing, n (%)	599 (8.5%)	1860 (17.0%)	2459 (13.7%)
	Responders, n (%)	6412 (91.5%)	9130 (83.0%)	15542 (86.3%)
	Always, n (%)	*6091 (95.0%)*	*8523 (93.4%)*	*14614 (94.0%)*
	Often, n (%)	*250 (3.9%)*	*395 (4.3%)*	*645 (4.1%)*
	Sometimes, n (%)	*37 (0.6%)*	*111 (1.2%)*	*148 (1.0%)*
	Not very often, n (%)	*16 (0.2%)*	*40 (0.4%)*	*56 (0.4%)*
	Never, n (%)	*18 (0.3%)*	*61 (0.7%)*	*79 (0.5%)*
11 (Q13)	On average, how often do you follow the guidance on social distance when outside of support/childcare bubbles, maintaining 1-2 metres between yourself and other people?	592 (8.4%)	1857 (16.9%)	2449 (13.6%)
	Missing, n (%)	6419 (91.6%)	9133 (83.1%)	15552 (86.4%)
	Responders, n (%)	*4196 (65.4%)*	*5966 (65.3%)*	*10162 (65.3%)*
	Always, n (%)	*1790 (27.9%)*	*2496 (27.3%)*	*4286 (27.6%)*
	Often, n (%)	*324 (5.0%)*	*475 (5.2%)*	*799 (5.1%)*
	Sometimes, n (%)	*87 (1.4%)*	*136 (1.5%)*	*223 (1.4%)*
	Not very often, n (%)	*22 (0.3%)*	*60 (0.7%)*	*82 (0.5%)*
	Never, n (%)			
12 (Q14)	In the past seven days, have you had any visitors inside your home from outside your support/childcare bubbles, including trades people, carers or medical staff?	635 (9.1%)	1977 (18.0%)	2612 (14.5%)
	Missing, n (%)	6376 (90.9%)	9013 (82.0%)	15389 (85.5%)
	Responders, n (%)	*931 (14.6%)*	1466 (16.3%)	*2397 (15.6%)*
	Yes, n (%)	*5445 (85.4%)*	7547 (83.7%)	*12992 (84.4%)*
	No, n (%)			
13 (Q15)	When you have had a visitor inside your home, which of the following actions did you take to reduce the spread of the coronavirus (COVID-19)?			
	Missing, n (%)	1499 (21.4%)	3192 (29.0%)	4691 (26.1%)
	Responders, n (%)	5512 (78.6%)	7798 (71.0%)	13310 (73.9%)
	Worn a face mask, n (%)	*2518 (45.7%)*	*3805 (48.8%)*	*6323 47.5%)*
	Asked the visitor to wear a mask, n (%)	*2209 (40.1%)*	*3177 (40.7%)*	*5386 (40.5%)*

	Opened windows or doors, n (%)	*3090 (56.1%)*	*4288 (55.0%)*	*7378 (55.4%)*
	Cleaned touch points, n (%)	*2158 (39.2%)*	*3189 (40.9%)*	*5347 40.2%)*
	Maintained social distancing, n (%)	*3751 (68.1%)*	*5162 (66.2%)*	*8913 (67.0%)*
	Washed hands regularly, n (%)	*3181 (57.7%)*	*4420 (56.7%)*	*7601 (57.1%)*
	Other, n (%)	*211 (3.8%)*	*257 (3.3%)*	*468 (3.5%)*
	None of the above, n (%)	*974 (17.7%)*	*1358 (17.4%)*	*2332 (17.5%)*
14 (Q16)	As part of your condition for attendance to this event, you will be required to complete a test. In case that this test results comes back negative, which statement below best describes what it means to you?			
	Missing, n (%)	593 (8.5%)	1924 (17.5%)	2517 (14.0%)
	Responders, n (%)	6418 (91.5%)	9066 (82.5%)	15484 (86.0%)
	I am definitely not infectious, n (%)	*2138 (33.3%)*	*3401 (37.5%)*	*5539 (35.8%)*
	I am probably not infectious, n (%)	*4004 (62.4%)*	*5213 (57.5%)*	*9217 (59.5%)*
	I am probably infectious, n (%)	*101 (1.6%)*	*148 (1.6%)*	*249 (1.6%)*
	I am definitely infectious, n (%)	*65 (1.0%)*	*90 (1.0%)*	*155 (1.0%)*
	Don't know, n (%)	*110 (1.7%)*	*214 (2.4%)*	*324 (2.1%)*
15 (Q18)	What gender do you most identify with?			
	Missing, n (%)	593 (8.5%)	1965 (17.9%)	2558 (14.2%)
	Responders, n (%)	6418 (91.5%)	9025 (82.1%)	15443 (85.8%)
	Man, n (%)	*3037 (47.3%)*	*4056 (44.9%)*	*7093 (45.9%)*
	Woman, n (%)	*3304 (51.5%)*	*4771 (52.9%)*	*8075 (52.3%)*
	Non-binary, n (%)	*30 (0.47%)*	*89 (1.0%)*	*119 (0.8%)*
	Other, n (%)	*1 (0.02%)*	*13 (0.1%)*	*14 (0.1%)*
	Prefer not to say, n (%)	*46 (0.7%)*	*96 (1.1%)*	*142 (0.9%)*
16 (Q19)	Ethnicity			
	Missing, n (%)	596 (8.5%)	1978 (18.0%)	2574 (14.3%)
	Responders, n (%)	6415 (91.5%)	9012 (82.0%)	15427 (85.7%)
	Asian/Asian-British- Indian, Pakistani, Bangladeshi, other, n (%)	*89(1.4%)*	*155 (1.7%)*	*244 (1.6%)*
	Black/Black British- Caribbean. African, other, n (%)	*24 (0.4%)*	*44 (0.5%)*	*68 (0.4%)*
	Mixed race- White and Black/Black British, n (%)	*71 (1.1%)*	*162 (1.8%)*	*2331.5%)*
	Mixed race- other, n (%)	*93 (1.4%)*	*128 (1.4%)*	*221 (1.4%)*
	White- British, Irish, other, n (%)	*6026 (93.9%)*	*8274 (91.8%)*	*14300 (92.7%)*
	Chinese/Chinese British, n (%)	*15 (0.2%)*	*45 (0.5%)*	*60 (0.4%)*
	Middle Eastern/Middle Eastern British- Arab, Turkish, other, n (%)	*15 (0.2%)*	*27 (0.3%)*	*42 (0.3%)*
	Other ethnic group, n (%)	*17 (0.3%)*	*13 (0.1%)*	*30 (0.2%)*
	Prefer not to say, n (%)	*65 (1.0%)*	*164 (1.8%)*	*229 (1.5%)*
17 (Q21)	Have you previously been diagnosed with Covid?			
	Missing, n (%)	591 (8.4%)	2008 (18.3%)	2599 (14.4%)
	Responders, n (%)	6420 (91.6%)	8982 (81.7%)	15402 (85.6%)
	Yes, I've had a positive test (antibody or swab), n (%)	*1817 (28.3%)*	*2418 (26.9%)*	*4235 (27.5%)*
	Yes, most likely but I've not had a test to confirm, n (%)	*1148 (17.9%)*	*1315 (14.6%)*	*2463 (16.0%)*
	No, don't think I've had Covid-19, n (%)	*3092 (48.2%)*	*4618 (51.4%)*	*7710 (50.0%)*
	Don't know, n (%)	*363 (5.7%)*	*631 (7.0%)*	*994 (6.5%)*
18 (Q23)	Have you received your vaccination?			
	Missing, n (%)	605 (8.6%)	2031 (18.5%)	2636 (14.6%)
	Responders, n (%)	6406 (91.4%)	8959 (81.5%)	15365 (85.4%)
	Yes, I have received my first vaccine dose, n (%)	*1342 (20.9%)*	*1958 (21.8%)*	*3300 (21.4%)*
	Yes, I have received both vaccine doses, n (%)	*766 (11.9%)*	*1072 (12.0%)*	*1838 (12.0%)*

	No, n (%)	*4278 (66.8%)*	*5840 (65.2%)*	*10118 (65.9%)*
	Don't know, n (%)	*20 (0.3%)*	*89 (1.0%)*	*109 (0.7%)*

Pre-event screening questionnaire variables by attendance (whether the linked ticket was scanned or not) from Sefton Park

	Variable	Summary		
		Tickets scanned	Tickets not scanned	Overall
1	Number of participants (with completed pre-event screening questionnaire)	6101	910	7011
2 (Q2)	Are you concerned about attending the event?			
	Missing, n (%)	239 (3.9%)	341 (37.5%)	580 (8.3%)
	Responders, n (%)	5862 (96.1%)	569 (62.5%)	6431 (91.7%)
	Not at all concerned, n (%)	*4353 (74.3%)*	*413 (72.6%)*	*4766 (74.1%)*
	Slightly concerned, n (%)	*1204 (20.5%)*	*124 (21.8%)*	*1328 (20.6%)*
	Moderately concerned, n (%)	*272 (4.6%)*	*28 (4.9%)*	*300 (4.7%)*
	Very concerned, n (%)	*33 (0.6%)*	*4 (0.7%)*	*37 (0.6%)*
3 (Q3)	Are there any factors that might contribute to some of your concern about attending the event?			
	Missing, n (%)	4631(75.9%)	756(83.1%)	5387(76.8%)
	Responders, n (%)	1470 (24.1%)	154 (16.9%)	1624 (23.2%)
	Possibly catching Covid-19, n (%)	*937 (63.7%)*	*99 (64.3%)*	*1036 (63.8%)*
	Impact of having to self-isolate if I test positive for Covid-19, n (%)	*634 (43.1%)*	*74 (48.1%)*	*708 (43.6%)*
	Social anxiety, n (%)	*401 (27.3%)*	*43 (27.9%)*	*444 (27.3%)*
	Others thinking I'm reckless, n (%)	*586 (39.9%)*	*57 (37.0%)*	*643 (39.6%)*
	Other, n (%)	*621 (42.2%)*	*60 (39.0%)*	*681 (41.9%)*
4 (Q4)	How likely do you think you are to catch coronavirus at the event?			
	Missing, n (%)	247 (4.0%)	342 (37.6%)	589 (8.4%)
	Responders, n (%)	5854 (96.0%)	568 (62.4%)	6422 (91.6%)
	Very unlikely, n (%)	*2227 (38.0%)*	*194 (34.2%)*	*2421 (37.7%)*
	Fairly unlikely, n (%)	*2643 (45.1%)*	*264 (46.5%)*	*2907 (45.3%)*
	Neither unlikely nor likely, n (%)	*829 (14.2%)*	*93 (16.4%)*	*922 (14.4%)*
	Fairly likely, n (%)	*140 (2.4%)*	*16 (2.8%)*	*156 (2.4%)*
	Very likely, n (%)	*15 (0.3%)*	*1 (0.2%)*	*16 (2.5%)*
5 (Q7)	How concerned are you about potentially infecting others after attending the event?			
	Missing, n (%)	253 (4.1%)	342 (37.6%)	595 (8.5%)
	Responders, n (%)	5848 (95.9%)	568 (62.4%)	6416 (91.5%)
	Not at all concerned, n (%)	*2706 (46.3%)*	*236 (41.5%)*	*2942 (45.9%)*
	Slightly concerned, n (%)	*1604 (27.4%)*	*172 (30.3%)*	*1776 (27.7%)*
	Neither concerned nor unconcerned, n (%)	*840 (14.4%)*	*74 (13.0%)*	*914 (14.2%)*
	Moderately concerned, n (%)	*617 (10.6%)*	*77 (13.6%)*	*694 (10.8%)*
	Very concerned, n (%)	*81 (1.4%)*	*9 (1.6%)*	*90 (1.4%)*
6 (Q8)	If 'not at all concerned' on last question, having stated that you are not concerned, share reasons why			
	Missing, n (%)	3415 (56.0%)	675 (74.2%)	4090 (58.3%)
	Responders, n (%)	2686 (44.0%)	235 (25.8%)	2921 (41.7%)
	Because I've previously had Covid-19, n (%)	*995 (37.0%)*	*69 (29.4%)*	*1064 (36.4%)*

	Because I've been tested, n (%)	*1411 (52.5%)*	*116 (49.4%)*	*1527 (52.3%)*
	Because I've been vaccinated, n (%)	*898 (33.4%)*	*74 (31.5%)*	*972 (33.3%)*
	Because I've followed guidance on reducing the spread of Covid-19, n (%)	*1749 (65.1%)*	*150 (63.8%)*	*1899 (65.0%)*
	Other, n (%)	*99 (3.7%)*	*11 (4.7%)*	*110 (3.8%)*
7 (Q9)	How important do you think it is to resume these kinds of public events as soon as possible?			
	Missing, n (%)	246 (4.0%)	345 (37.9%)	591 (8.4%)
	Responders, n (%)	5855 (96.0%)	565 (62.1%)	6420 (91.6%)
	Not at all important, n (%)	*40 (0.7%)*	*6 (1.1%)*	*46 (0.7%)*
	Slightly important, n (%)	*129 (2.2%)*	*17 (3.0%)*	*146 (2.3%)*
	Neither important nor unimportant, n (%)	*167 (2.9%)*	*18 (3.2%)*	*185 (2.9%)*
	Moderately important, n (%)	*1364 (23.3%)*	*134 (23.7%)*	*1498 (23.3%)*
	Very important, n (%)	*4155 (70.9%)*	*390 (69.0%)*	*4545 (70.8%)*
8 (Q10)	In the past seven days, how often did you wash your hands with soap and water straight away after returning home from a public place?			
	Missing, n (%)	244 (4.0%)	342 (37.6%)	586 (8.4%)
	Responders, n (%)	5857 (96.0%)	568 (62.4%)	6425 (91.6%)
	Always, n (%)	*4344 (74.2%)*	*381 (67.1%)*	*4725 (73.5%)*
	Often, n (%)	*1240 (21.2%)*	*148 (26.0%)*	*1388 (21.6%)*
	Sometimes, n (%)	*186 (3.2%)*	*26 (4.6%)*	*212 (3.3%)*
	Not very often, n (%)	*54 (0.9%)*	*10 (1.8%)*	*64 (1.0%)*
	Never, n (%)	*33 (0.5%)*	*3 (0.5%)*	*36 (0.6%)*
9 (Q11)	In the past seven days, have you used a face covering when outside your home to help slow the spread of the coronavirus (COVID-19)?			
	Missing, n (%)	252 (4.1%)	345 (37.9%)	597 (8.5%)
	Responders, n (%)	5849 (95.9%)	565 (62.1%)	6414 (91.5%)
	Yes, n (%)	*5688 (97.2%)*	*550 (97.3%)*	*6238 (97.3%)*
	No, n (%)	*128 (2.2%)*	*12 (2.1%)*	*140 (2.2%)*
	Not applicable, n (%)	*33 (0.6%)*	*3 (0.5%)*	*36 (0.6%)*
10 (Q12)	While you were inside a public space (e.g., shop, public transport) in the last seven days, how often did you wear a protective face covering to help slow the spread of the coronavirus (COVID-19)?			
	Missing, n (%)	256 (4.2%)	343 (37.7%)	599 (8.5%)
	Responders, n (%)	5845 (95.8%)	567 (62.3%)	6412 (91.5%)
	Always, n (%)	*5558 (95.1%)*	*533 (94.0%)*	*6091 (95.0%)*
	Often, n (%)	*223 (3.8%)*	*27 (4.8%)*	*250 (3.9%)*
	Sometimes, n (%)	*33 (0.6%)*	*4 (0.7%)*	*37 (0.6%)*
	Not very often, n (%)	*14 (0.2%)*	*2 (0.3%)*	*16 (0.2%)*
	Never, n (%)	*17 (0.3%)*	*1 (0.2%)*	*18 (0.3%)*
11 (Q13)	On average, how often do you follow the guidance on social distance when outside of support/childcare bubbles, maintaining 1-2 metres between yourself and other people?	250 (4.1%)	342 (37.6%)	592 (8.4%)
	Missing, n (%)	5851 (95.9%)	568 (62.4%)	6419 (91.6%)
	Responders, n (%)	*3815 (65.2%)*	*381 (67.1%)*	*4196 (65.4%)*
	Always, n (%)	*1648 (28.2%)*	*142 (25.0%)*	*1790 (27.9%)*
	Often, n (%)	*289 (4.9%)*	*35 (6.1%)*	*324 (5.0%)*

	Sometimes, n (%)	*78 (1.3%)*	*9 (1.6%)*	*87 (1.4%)*
	Not very often, n (%)	*21 (0.4%)*	*1 (0.2%)*	*22 (0.3%)*
	Never, n (%)			
12 (Q14)	In the past seven days, have you had any visitors inside your home from outside your support/childcare bubbles, including trades people, carers or medical staff?	288 (4.7%)	347 (38.1%)	635 (9.1%)
	Missing, n (%)	5813 (95.3%)	563 (61.9%)	6376 (90.9%)
	Responders, n (%)	*844 (14.5%)*	*87 (15.5%)*	*931 (14.6%)*
	Yes, n (%)	*4969 (85.5%)*	*476 (84.5%)*	*5445 (85.4%)*
	No, n (%)			
13 (Q15)	When you have had a visitor inside your home, which of the following actions did you take to reduce the spread of the coronavirus (COVID-19)?			
	Missing, n (%)	1065 (17.5%)	434 (47.7%)	1499 (21.4%)
	Responders, n (%)	5452 (82.5%)	476 (52.3%)	5512 (78.6%)
	Worn a face mask, n (%)	*2293 (45.5%)*	*225 (47.3%)*	*2518 (45.7%)*
	Asked the visitor to wear a mask, n (%)	*2012 (40.0%)*	*197 (41.4%)*	*2209 (40.1%)*
	Opened windows or doors, n (%)	*2846 (56.5%)*	*244 (51.3%)*	*3090 (56.1%)*
	Cleaned touch points, n (%)	*1981 (39.3%)*	*177 (37.2%)*	*2158 (39.2%)*
	Maintained social distancing, n (%)	*3438 (68.3%)*	*313 (65.8%)*	*3751 (68.1%)*
	Washed hands regularly, n (%)	*2912 (57.8%)*	*269 (56.5%)*	*3181 (57.7%)*
	Other, n (%)	*194 (3.9%)*	*17 (3.6%)*	*211 (3.8%)*
	None of the above, n (%)	*889 (17.7%)*	*85 (17.9%)*	*974 (17.7%)*
14 (Q16)	As part of your condition for attendance to this event, you will be required to complete a test. In case that this test results comes back negative, which statement below best describes what it means to you?			
	Missing, n (%)	251 (4.1%)	342 (37.6%)	593 (8.5%)
	Responders, n (%)	5850 (95.9%)	568 (62.4%)	6418 (91.5%)
	I am definitely not infectious, n (%)	*1967 (33.6%)*	*171 (30.1%)*	*2138 (33.3%)*
	I am probably not infectious, n (%)	*3630 (62.1%)*	*374 (65.8%)*	*4004 (62.4%)*
	I am probably infectious, n (%)	*91 (1.6%)*	*10 (1.8%)*	*101 (1.6%)*
	I am definitely infectious, n (%)	*60 (1.0%)*	*5 (0.9%)*	*65 (1.0%)*
	Don't know, n (%)	*102 (1.7%)*	*8 (1.4%)*	*110 (1.7%)*
15 (Q18)	What gender do you most identify with?			
	Missing, n (%)	250 (4.1%)	343 (37.7%)	593 (8.5%)
	Responders, n (%)	5851 (95.9%)	567 (62.3%)	6418 (91.5%)
	Man, n (%)	*2744 (46.9%)*	*293 (51.7%)*	*3037 (47.3%)*
	Woman, n (%)	*3038 (51.9%)*	*266 (46.9%)*	*3304 (51.5%)*
	Non-binary, n (%)	*26 (0.44%)*	*4 (0.7%)*	*30 (0.47%)*
	Other, n (%)	*1 (0.02%)*	*0 (0.0%)*	*1 (0.02%)*
	Prefer not to say, n (%)	*42 (0.7%)*	*4 (0.7%)*	*46 (0.7%)*
16 (Q19)	Ethnicity			
	Missing, n (%)	252 (4.1%)	344 (37.8%)	596 (8.5%)
	Responders, n (%)	5849 (95.9%)	566 (62.2%)	6415 (91.5%)
	Asian/Asian-British- Indian, Pakistani, Bangladeshi, other, n (%)			
	Black/Black British- Caribbean. African, other, n (%)	*81 (1.4%)*	*8 (1.4%)*	*89(1.4%)*
	Mixed race- White and Black/Black British, n (%)	*20 (0.4%)*	*4 (0.7%)*	*24 (0.4%)*
	Mixed race- other, n (%)	*60 (1.0%)*	*11 (1.9%)*	*71 (1.1%)*

	White- British, Irish, other, n (%)	*84 (1.4%)*	*9 (1.6%)*	*93 (1.4%)*
	Chinese/Chinese British, n (%)	*5505 (94.1%)*	*521 (92.0%)*	*6026 (93.9%)*
	Middle Eastern/Middle Eastern British- Arab, Turkish, other, n (%)	*12 (0.2%)*	*3 (0.5%)*	*15 (0.2%)*
	Other ethnic group, n (%)	*13 (0.2%)*	*2 (0.4%)*	*15 (0.2%)*
	Prefer not to say, n (%)	*16 (0.3%)*	*1 (0.2%)*	*17 (0.3%)*
		58 (1.0%)	*7 (1.2%)*	*65 (1.0%)*
17 (Q21)	Have you previously been diagnosed with Covid?			
	Missing, n (%)	249 (4.1%)	342 (37.6%)	591 (8.4%)
	Responders, n (%)	5852 (95.7%)	568 (62.4%)	6420 (91.6%)
	Yes, I've had a positive test (antibody or swab), n (%)	*1683 (28.8%)*	*134 (23.6%)*	*1817 (28.3%)*
	Yes, most likely but I've not had a test to confirm, n (%)	*1046 (17.9%)*	*102 (18.0%)*	*1148 (17.9%)*
	No, don't think I've had Covid-19, n (%)	*2797 (47.8%)*	*295 (51.9%)*	*3092 (48.2%)*
	Don't know, n (%)	*326 (5.6%)*	*37 (6.5%)*	*363 (5.7%)*
18 (Q23)	Have you received your vaccination?			
	Missing, n (%)	262 (4.3%)	343 (37.7%)	605 (8.6%)
	Responders, n (%)	5839 (95.7%)	567 (62.3%)	6406 (91.4%)
	Yes, I have received my first vaccine dose, n (%)	*1212 (20.8%)*	*130 (22.9%)*	*1342 (20.9%)*
	Yes, I have received both vaccine doses, n (%)	*706 (12.1%)*	*60 (10.6%)*	*766 (11.9%)*
	No, n (%)	*3903 (66.8%)*	*375 (66.1%)*	*4278 (66.8%)*
	Don't know, n (%)	*18 (0.3%)*	*2 (0.3%)*	*20 (0.3%)*

Post-event questionnaire results

Post-event questionnaire variables for each event.

	Variable	Good Business Festival	Circus Nightclub	Sefton Park
1	Number of participants (with completed pre-event screening questionnaire)	34	883	1421
2 (Q2)	How satisfied were you with the overall event?			
	Missing, n (%)	0 (0.0%)	91 (10.3%)	142 (10.0%)
	Responders, n (%)	34 (100.0%)	792 (89.7%)	1279 (90.0%)
	Extremely dissatisfied, n (%)	*0 (0.0%)*	*21 (2.6%)*	*30 (2.3%)*
	Somewhat dissatisfied, n (%)	*1 (3.0%)*	*6 (0.8%)*	*3 (0.2%)*
	Neither satisfied nor dissatisfied, n (%)	*0 (0.0%)*	*2 (0.3%)*	*2 (0.2%)*
	Moderately satisfied, n (%)	*8 (23.5%)*	*50 (6.3%)*	*98 (7.7%)*
	Extremely satisfied, n (%)	*25 (73.5%)*	*713 (90.0%)*	*1146 (89.6%)*
3 (Q4)	Thinking back to the event, please rate your experience and observation of physical distancing			
	Missing, n (%)	0 (0.0%)	92 (10.4%)	144 (10.1%)
	Responders, n (%)	34 (100.0%)	791 (89.6%)	1277 (89.9%)
	Maintained throughout the event, n (%)	*0 (0.0%)*	*22 (2.8%)*	*9 (0.7%)*
	Somewhat maintained at the event, n (%)	*10 (29.4%)*	*50 (6.3%)*	*180 (14.1%)*
	Not at all, n (%)	*24 (70.6%)*	*719 (90.9%)*	*1088 (85.2%)*
4 (Q5)	Thinking back to the event, please rate your experience and observation of wearing face-coverings			
	Missing, n (%)	0 (0.0%)	92 (10.4%)	143 (10.1%)
	Responders, n (%)	34 (100.0%)	791 (89.6%)	1278 (89.9%)
	Used throughout the event, n (%)	*0 (0.0%)*	*5 (0.6%)*	*3 (0.2%)*
	Somewhat observed at the event, n (%)	*5 (14.7%)*	*51 (6.4%)*	*175 (13.7%)*
	Not at all, n (%)	*29 (85.3%)*	*611 (77.3%)*	*994 (77.8%)*
	Not applicable, n (%)	*0 (0.0%)*	*124 (15.7%)*	*106 (8.3%)*
5 (Q6)	Thinking back to the event, please rate your experience and observation of hygiene arrangements			
	Missing, n (%)	1 (2.9%)	95 (10.8%)	146 (10.3%)
	Responders, n (%)	33 (97.1%)	788 (89.2%)	1275 (89.7%)
	Maintained throughout the event, n (%)	*17 (51.5%)*	*354 (44.9%)*	*640 (50.2%)*
	Somewhat observed at the event, n (%)	*12 (36.4%)*	*343 (43.5%)*	*556 (43.6%)*
	Not at all, n (%)	*4 (12.1%)*	*91 (11.6%)*	*79 (6.2%)*
6 (Q7)	Thinking back to the event, please rate your experience and observation of singing, chanting, shouting			
	Missing, n (%)	0 (0.0%)	94 (10.6%)	142 (10.0%)
	Responders, n (%)	34 (100.0%)	789 (89.4%)	1279 (90.4%)
	Occurred throughout the event, n (%)	*7 (20.6%)*	*723 (91.6%)*	*1246 (97.4%)*
	Occurred occasionally at the event, n (%)	*10 (29.4%)*	*60 (7.6%)*	*28 (2.2%)*
	Not at all, n (%)	*14 (41.2%)*	*4 (0.5%)*	*5 (0.4%)*
	Not applicable, n (%)	*3 (8.8%)*	*2 (0.3%)*	*0 (0.0%)*
7 (Q9)	Looking back at the event and your experience, how likely is it that you caught the coronavirus at the event?			
	Missing, n (%)	1 (2.9%)	92 (10.4%)	142 (10.0%)
	Responders, n (%)	33 (97.1%)	791 (89.6%)	1279 (90.0%)
	Very likely, n (%)	*0 (0.0%)*	*5 (0.6%)*	*5 (0.4%)*

	Fairly likely, n (%)	*0 (0.0%)*	*18 (2.3%)*	*27 (2.1%)*
	Neither unlikely nor likely, n (%)	*4 (12.1%)*	*155 (19.6%)*	*243 (19.0%)*
	Fairly unlikely, n (%)	*16 (48.5%)*	*319 (40.3%)*	*641 (50.1%)*
	Very unlikely, n (%)	*13 (39.4%)*	*294 (37.2%)*	*363 (28.4%)*
8 (Q12)	How concerned are you about potentially infecting others after attending the event?			
	Missing, n (%)	0 (0.0%)	95 (10.8%)	142 (10.0%)
	Responders, n (%)	34 (100.0%)	788 (89.2%)	1279 (90.0%)
	Not at all concerned, n (%)	*15 (44.1%)*	*498 (63.2%)*	*695 (54.3%)*
	Slightly concerned, n (%)	*15 (44.1%)*	*206 (26.2%)*	*452 (35.4%)*
	Moderately concerned, n (%)	*4 (11.8%)*	*72 (9.1%)*	*115 (9.0%)*
	Very concerned, n (%)	*0 (0.0%)*	*12 (1.5%)*	*17 (1.3%)*
9 (Q13)	If 'not at all concerned' on last question, having stated that you are not concerned, share reasons why			
	Missing, n (%)	19 (55.9%)	390 (44.2%)	729 (51.3%)
	Responders, n (%)	15 (44.1%)	493 (55.8%)	692 (48.7%)
	Because I've previously had Covid-19, n (%)	*1 (6.7%)*	*170 (34.5%)*	*212 (30.6%)*
	Because I've been tested, n (%)	*11 (73.3%)*	*431 (87.4%)*	*592 (85.5%)*
	Because I've been vaccinated, n (%)	*11 (73.3%)*	*136 (27.6%)*	*269 (38.9%)*
	Because I kept my mask on, n (%)	*0 (0.0%)*	*2 (0.4%)*	*3 (0.4%)*
	Because I kept my distance from other people at the event,	*1 (6.7%)*	*8 (1.6%)*	*47 (6.8%)*
	n (%)	*8 (53.3%)*	*224 (45.4%)*	*349 (50.4%)*
	Because I sanitised/washed my hands regularly, n (%)	*1 (6.7%)*	*41 (8.3%)*	*66 (9.5%)*
	Other, n (%)			
10 (Q14)	Looking back at the event and your experience, how confident are you about the prospect of safely resuming these kinds of events as soon as possible?			
	Missing, n (%)	0 (0.0%)	92 (10.4%)	144 (10.1%)
	Responders, n (%)	34 (100.0%)	791 (89.6%)	1277 (87.2%)
	Not at all confident, n (%)	*0 (0.0%)*	*2 (0.3%)*	*1 (0.08%)*
	Slightly confident, n (%)	*3 (8.8%)*	*34 (4.3%)*	*79 (6.19%)*
	Neither confident nor unconfident, n (%)	*0 (0.0%)*	*16 (2.0%)*	*27 (2.11%)*
	Moderately confident, n (%)	*17 (50.0%)*	*150 (18.9%)*	*395 (30.93%)*
	Very confident, n (%)	*14 (41.2%)*	*589 (74.5%)*	*775 (60.69%)*
11 (Q15)	If similar events in the future would require attendees to present a 'Covid passport' in order to enter, how likely are you to join such events?			
	Missing, n (%)	0 (0.0%)	92 (10.4%)	143 (10.1%)
	Responders, n (%)	34 (100.0%)	791 (89.6%)	1278 (89.9%)
	Very likely, n (%)	*20 (58.8%)*	*379 (47.9%)*	*736 (57.6%)*
	Fairly likely, n (%)	*7 (20.6%)*	*161 (20.4%)*	*255 (20.0%)*
	Neither unlikely nor likely, n (%)	*3 (8.8%)*	*91 (11.5%)*	*115 (9.0%)*
	Fairly unlikely, n (%)	*1 (2.9%)*	*61 (7.7%)*	*86 (6.7%)*
	Very unlikely, n (%)	*3 (8.8%)*	*99 (12.5%)*	*86 (6.7%)*
12 (Q16)	How did you travel to the event?			
	Missing, n (%)	0 (0.0%)	99 (11.2%)	144 (10.1%)
	Responders, n (%)	34 (100%)	784 (88.8%)	1277 (89.9%)
	Public transport, n (%)	*8 (23.5%)*	*50 (6.4%)*	*188 (14.7%)*
	Drove own vehicle, n (%)	*13 (38.2%)*	*21 (2.7%)*	*77 (6.0%)*
	Dropped off by friend/family, n (%)	*7 (20.6%)*	*139 (17.7%)*	*153 (12.0%)*
	Taxi, n (%)	*1 (2.9%)*	*542 (69.1%)*	*375 (29.4%)*

	Walk, n (%)	*5 (14.7%)*	*30 (3.8%)*	*476 (37.3%)*
	Cycled, n (%)	*0 (0.0%)*	*2 (0.3%)*	*4 (0.3%)*
	Other, n (%)	*0 (0.0%)*	*0 (0.0%)*	*4(0.3%)*
13 (Q17)	How did you travel from the event?			
	Missing, n (%)	0 (0.0%)	99 (11.2%)	145 (10.2%)
	Responders, n (%)	34 (100.0%)	784 (88.8%)	1276 (89.8%)
	Public transport, n (%)	*5 (14.7%)*	*47 (6.0%)*	*155 (12.1%)*
	Drove own vehicle, n (%)	*13 (38.2%)*	*7 (0.9%)*	*53 (4.2%)*
	Dropped off by friend/family, n (%)	*8 (23.5%)*	*110 (14.0%)*	*165 (12.9%)*
	Taxi, n (%)	*2 (5.9%)*	*459 (58.6%)*	*342 (26.8%)*
	Walk, n (%)	*6 (17.7%)*	*159 (20.3%)*	*544 (42.6%)*
	Cycled, n (%)	*0 (0.0%)*	*1 (0.1%)*	*0 (0.0%)*
	Other, n (%)	*0 (0.0%)*	*1 (0.1%)*	*17 (1.3%)*
14 (Q18)	To the best of your recollection, how much alcohol did you consume prior to and during the event?			
	Missing, n (%)	0 (0.0%)	101 (11.4%)	145 (10.2%)
	Responders, n (%)	34 (100.0%)	782 (88.6%)	1276 (89.8%)
	None at all, n (%)	*6 (17.7%)*	*32 (4.1%)*	*54 (4.2%)*
	1-2, n (%)	*19 (55.9%)*	*61 (7.8%)*	*90 (7.1%)*
	3-4, n (%)	*3 (8.8%)*	*136 (17.4%)*	*185 (14.5%)*
	5-6, n (%)	*5 (14.7%)*	*189 (24.2%)*	*276 (21.6%)*
	7-9, n (%)	*1 (2.9%)*	*164 (21.0%)*	*254 (19.9%)*
	10+, n (%)	*0 (0.0%)*	*200 (25.5%)*	*417 (32.7%)*
15 (Q19)	Did you come as part of a group?			
	Missing, n (%)	0 (0.0%)	105 (11.9%)	147 (10.3%)
	Responders, n (%)	34 (100.0%)	778 (88.1%)	1274 (89.7%)
	Yes, n (%)	*10 (29.4%)*	*739 (95.0%)*	*1132(88.9%)*
	No, n (%)	*24 (70.6%)*	*39 (5.0%)*	*142 (11.1%)*
16 (Q20)	If yes, how many people in your party?			
	Missing, n (%)	24 (70.6%)	145 (16.4%)	289 (20.3%)
	Responders, n (%)	10 (29.4%)	738 (83.6%)	1132 (79.7%)
	2, n (%)	*7 (70.0%)*	*103 (14.0%)*	*224 (19.8%)*
	3, n (%)	*1 (10.0%)*	*83 (11.2%)*	*147 (13.0%)*
	4, n (%)	*1 (10.0%)*	*104 (14.1%)*	*184 (16.3%)*
	5, n (%)	*1 (10.0%)*	*104 (14.1%)*	*134 (11.8%)*
	6 or more, n (%)	*0 (0.0%)*	*344 (46.6%)*	*443 (39.1%)*
17 (Q21)	Did you scan the NHS App at the event?			
	Missing, n (%)	0 (0.0%)	103 (11.7%)	147 (10.3%)
	Responders, n (%)	34 (100.0%)	780 (88.3%)	1274 (89.7%)
	Yes, n (%)	*8 (23.5%)*	*101 (12.9%)*	*124 (9.7%)*
	No, n (%)	*26 (76.5%)*	*679 (87.1%)*	*1150 (90.3%)*
18 (Q23)	What gender do you most identify with?			
	Missing, n (%)	0 (0.0%)	102 (11.6%)	148 (10.4%)
	Responders, n (%)	34 (100.0%)	781 (88.4%)	1273 (89.6%)
	Man, n (%)	*14 (41.2%)*	*269 (34.4%)*	*485 (38.1%)*
	Woman, n (%)	*20 (58.8%)*	*510 (65.3%)*	*778 (61.1%)*
	Non-binary, n (%)	0 (0.0%)	*1 (0.1%)*	*4 (0.3%)*
	Other, n (%)	0 (0.0%)	*0 (0.0%)*	*0 (0.0%)*
	Prefer not to say, n (%)	0 (0.0%)	*1 (0.1%)*	*6 (0.5%)*
19 (Q24)	Ethnicity			
	Missing, n (%)	0 (0.0%)	103 (11.7%)	147 (10.3%)

	Responders, n (%)	34 (100.0%)	780 (88.3%)	1274 (89.7%)
	Asian/Asian-British- Indian, Pakistani, Bangladeshi, other, n	0 (0.0%)	*14 (1.8%)*	*7 (0.5%)*
	(%)	0 (0.0%)	*3 (0.4%)*	*4 (0.3%)*
	Black/Black British- Caribbean. African, other, n (%)	0 (0.0%)	*22 (2.8%)*	*9 (0.7%)*
	Mixed race- White and Black/Black British, n (%)	0 (0.0%)	*12 (1.5%)*	*19 (1.5%)*
	Mixed race- other, n (%)	*33 (97.1%)*	*719 (92.2%)*	*1220 (95.8%)*
	White- British, Irish, other, n (%)	0 (0.0%)	*2 (0.3%)*	*3 (0.2%)*
	Chinese/Chinese British, n (%)	0 (0.0%)	*2 (0.3%)*	*1 (0.1%)*
	Middle Eastern/Middle Eastern British- Arab, Turkish,	*1 (2.9%)*	*2 (0.3%)*	*2 (0.2%)*
	other, n (%)	0 (0.0%)	*4 (0.5%)*	*9 (0.7%)*
	Other ethnic group, n (%)			
	Prefer not to say, n (%)			
20 (Q26)	Have you previously been diagnosed with Covid?			
	Missing, n (%)	0 (0.0%)	105 (11.9%)	149 (10.5%)
	Responders, n (%)	34 (100.0%)	778 (88.1%)	1272 (89.5%)
	Yes, I've had a positive test (antibody or swab), n (%)	*3 (8.8%)*	*233 (29.9%)*	*324 (25.5%)*
	Yes, most likely but I've not had a test to confirm, n (%)	*3 (8.8%)*	*177 (22.8%)*	*231 (18.1%)*
	No, don't think I've had Covid-19, n (%)	*24 (70.6%)*	*327 (42.0%)*	*651 (51.2%)*
	Don't know, n (%)	*4 (11.8%)*	*41 (5.3%)*	*66 (5.2%)*
21 (Q28)	Have you received your vaccination?			
	Missing, n (%)	0 (0.0%)	103 (11.7%)	147 (10.3%)
	Responders, n (%)	34 (100.0%)	780 (88.3%)	1274 (89.7%)
	Yes, I have received my first vaccine dose, n (%)	*23 (67.7%)*	*136 (17.5%)*	*297 (23.3%)*
	Yes, I have received both vaccine doses, n (%)	*3 (8.8%)*	*100 (12.8%)*	*208 (16.3%)*
	No, n (%)	*8 (23.5%)*	*543 (69.6%)*	*769 (60.4%)*
	Don't know, n (%)	*0 (0.0%)*	*1 (0.1%)*	*0 (0.0%)*
